# Reactivity of a model of B_3_P_3_-doped nanographene with up to three CO_2_ molecules

**DOI:** 10.1038/s41598-023-29336-y

**Published:** 2023-02-10

**Authors:** Maxime Ferrer, Ibon Alkorta, Jose Elguero, Josep M. Oliva-Enrich

**Affiliations:** 1 Instituto de Química Médica (CSIC), Juan de la Cierva, 3, 28006 Madrid, Spain; 2 Doctoral School, Universidad Autónoma de Madrid, 28049 Madrid, Spain; 3 Instituto de Química-Física Rocasolano (CSIC), Serrano, 119, 28006 Madrid, Spain

**Keywords:** Reaction mechanisms, Structure prediction

## Abstract

The reactivity of a B_3_P_3_-doped hexa-cata-hexabenzocoronene, as a model of nanographene (B_3_P_3_-NG), towards carbon dioxide was studied at the DFT M06-2X/6-311++G(3df,3pd)//M06-2X/6-31+G* level of theory. This compound can be classified as a poly-cyclic poly-Frustrated Lewis Pair (FLP) system, as it presents more than one Lewis Acid/Lewis Base pair on its surface, making the capture of several carbon dioxide molecules possible. Two scenarios were considered to fully characterize the capture of CO_2_ by this multi-FLP system: (i) fixation of three CO_2_ molecules sequentially one by one; and (ii) simultaneous contact of three CO_2_ molecules with the B_3_P_3_-NG surface. The resulting adducts were analyzed as function of activation barriers and the relative stability of the CO_2_ capture. A cooperativity effect due to the π-delocalization of the hexa-cata-hexabenzocoronene is observed. The fixation of a CO_2_ molecule modifies the electronic properties. It enhances the capture of additional CO_2_ molecules by changing the acidy and basicity of the rest of the boron and phosphorus atoms in the B_3_P_3_-NG system.

## Introduction

Carbon dioxide, CO_2_, is a very stable molecule resulting from the oxidation of mineral carbon or carbon chains in organic molecules. It is the most abundant greenhouse gas emitted by human activities^1–3^. Several small molecules, such as carbenes^4–9^, guanidines^10–12^ and phosphines^13–16^, have been shown to be able to form adducts with CO_2_. Even though CO_2_ has been used as potential building block in organic synthesis^17–20^ mimicking the photosynthesis of plants, more efforts seem necessary to reduce the impact of the surplus human production of this molecule.

Frustrated Lewis Pair (FLP)^21–23^ systems, which are characterized by not being able to form Lewis acid-Lewis base adducts, have shown interesting abilities to activate stable molecules such as CO_2_, N_2_ or H_2_^24–26^. A number of experimental and theoretical studies on the activation and sequestration of CO_2_ by FLP are available in the literature^27–31^.

**Figure 1:**
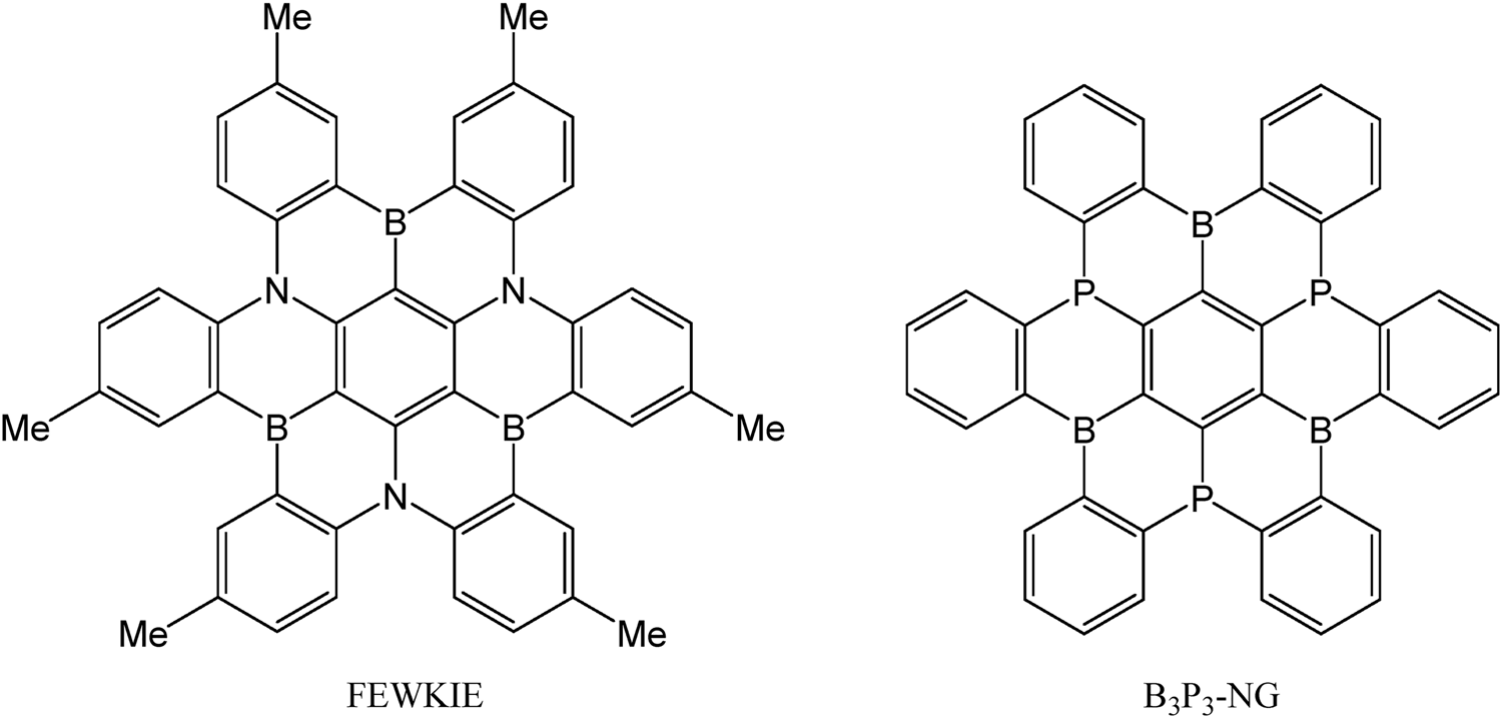
Structure of CSD Refcode FEWKIE and the multi-FLP B_3_P_3_-NG used in this study.

The structure of a derivative of hexabenzo[*a,d,g,j,m,p*]coronene or hexa-*cata*-hexabenzocoronene with N-B atoms in relative *para* positions have been described in the literature^32^ and its X-ray structure is available in the CSD^33^ (Refcode: FEWKIE) (Fig. 1). Several groups have studied the interaction of nanographene and doped nanographene with CO_2_, and they only found the formation of non-covalent complexes^34–38^. In these complexes, the CO_2_ molecule is not activated since it shows geometrical characteristics closed to the ones in the isolated CO_2_ (C–O bonds around 1.17 Å, O–C–O around 179°).

The potential interaction and activation of carbon dioxide by 5,10-disubstituted dibenzoazaborinines and dibenzophosphaborines intramolecular-FLP was investigated by us recently^39^. It was observed that both were able to interact with CO_2_ due to their FLP features. However, the dibenzophosphaborine with a P/B couple was found to be more effective, mainly due to the lower delocalization of the phosphorus lone pair into the aromatic rings, and its higher tendency to be hypervalent. Thus, combining our previous research and the structure of FEWKIE, we decided to study the potential used of triphosphatriborahexabenzo [*a*,*d*,*g*,*j*,*m*,*p*]coronene (B_3_P_3_-NG in Fig. 1) as potential multi-FLP molecule to capture and activate CO_2_.

The structure of B_3_P_3_-NG (NG : nanographene) can be envisioned as the overlap of 6 dibenzophosphaborines sharing a common side phenyl ring (Fig. 1). In other words, the structure presents 6 B/P pairs. Potentially, up to 12 B/P interaction sites can be found in the molecule if its two faces are non-equivalent. Consequently, the possibility that B_3_P_3_-NG interacts and form adducts with up to three CO_2_ molecules has been examined. Also, the potential cooperativity effects on adducts formation have been explored and interpreted.

## Computational methods

Using the scientific software Gaussian16^40^, the structures under study were optimized with the M06-2X DFT functional^41^ and the 6-31+G(d) basis set^42^. In order to validate this model, the monomer and one adduct were also optimized at the M06-2X/6-311+ +G(3df,3pd) level of theory^43^. The calculated root-mean-square deviation (RMSD) between the structures obtained with the M06-2X/6-31+G(d) model and those with the M06-2X/6-311++G(3df,3pd) model was found to be 0.03 Å. In other words, the selected level of theory (M06-2X/6-31+G*) is adequate in order to obtain structures similar to those with the more extended basis set, M06-2X/6-311++G(3df,3pd), at a lower computational cost. The optimized geometries were checked to be minima, no imaginary frequency, or transition states (TS), one imaginary frequency, by means of frequency calculations at M06-2X/6-31+G(d) computational level.

In order to obtain more accurate energies, the electronic energies of all the compounds were recalculated by running single point calculations at the M06-2X/6-311++G(3df,3pd) level of theory using the M06-2X/6-31+G(d) geometries.

The Molecular Electrostatic Potential (MEP) corresponds to the interaction energy between a given molecule and a non-polarizable + 1.0 e positive charge. The MEP enables to localize regions that a priori make a favorable interaction with a positive charge (minima of the MEP) and with a negative one (maxima of the MEP). The MEP of B_3_P_3_-NG has been calculated on the 0.001 a.u. electron density isosurface to predict the most probable positions to fix the CO_2_ molecules. These positions should present a maximum and a minimum, relatively close in space, enabling a double interaction of B_3_P_3_-NG with one of the nucleophilic oxygens and the electrophilic carbon. These calculations were done using the M06-2X/6-311++G(3df,3pd) model and the Multiwfn software^44^.

The topological properties of the electron density for the systems were analyzed by means of the quantum theory of atoms in molecules (QTAIM) model^45,46^, implemented in the scientific software AIMAll^47^. The points where the density gradient vanishes are called electron density critical points (CPs). By diagonalizing the Hessian matrix at those points (second derivative matrix of the electron density with respect to electron position), the CPs can be classified depending on the number of non-zero eigenvalues (rank, w) and the sum of the eigenvalues signs (signature, s): CP(w,s). Usually, chemists are interested in the localization of the attractor (3; − 3), bond (3; − 1), ring (3; + 1) and cage critical points (3; + 3). The covalent character of the interactions associated to the bond critical points can be determined by looking at the values of the electron density, the Laplacian, the potential and the kinetic energy density values^48,49^. In the present study, the molecular graphs were computed and plotted with the AIMAll software at the M06-2X/6-311++G(3df,3pd) level of theory.

The non-covalent interactions are generally characterized by a low electron density between the two atoms or group of atoms interacting. A way to characterize the non-covalent interactions of a system is to use the non-covalent interaction index (NCI)^50^. This index enables to localize interaction regions studying the reduced density gradient. A complementary method to identify NCI is the independent gradient model (IGM), implemented in the IGMPlot program^51,52^. This method is based on the difference between the non interacting density gradient and the real density gradient, δG^51,52^. Regions of gradient attenuation indicate the presence of an interaction. Interaction regions are characterized by a positive δG, and the strength of the interactions can be determined by integrating δG into the interaction surface volumes.

In addition, IGMPlot gives the possibility to characterize the strength of a given interaction by calculating its intrinsic bond strength index^53^ (IBSI, Eq. 1): the larger the IBSI, the stronger the bond. It is necessary to remember that the IBSI is not linked to a bond order, but to the force constant k of the bond or interaction. Thus, it is an intrinsic dynamic property of the bond.

1 $${\text{IBSI}}_{{{\text{AB}}}} = \frac{{\int_{{\text{V}}} {\frac{{{\delta G}_{{{\text{AB}}}} }}{{{\text{d}}_{{{\text{AB}}}}^{2} }}{\text{dV}}} }}{{\int_{{\text{V}}} {\frac{{{\delta G}_{{{\text{H}}_{2} }} }}{{{\text{d}}_{{{\text{H}}_{2} }}^{2} }}{\text{dV}}} }}$$

The binding energy of adducts and complexes has been calculated as the difference of its energy and the sum of the isolated monomers in their minimum configuration (Eq. 2). In order to evaluate the potential cooperative effect when several molecules of CO_2_ interact with B_3_P_3_-NG, the total binding energy of the adducts has been decomposed (Eq. 3) into a deformation energy of the monomers (E_def_) (Eq. 4), the two-body interaction energy [Δ^2^E(ij)] (Eq. 5) and a cooperative energy (C); E(i) is the energy of the isolated monomer in its minimum energy and E^′^(i) the energy in the geometry of the complex. Thus, this treatment is similar to the many body energy analysis^54,55^ but truncating the expansion in the two-body interaction term and including the higher terms in the cooperativity component.

2 $${\text{Eb }} = {\text{ E}}\left( {\text{adduct or complex}} \right) \, {-}{\text{ E}}\left( {{\text{B}}_{{3}} {\text{P}}_{{3}}{\text{-NG}}} \right) \, {-}{\text{ n}}*{\text{E}}\left( {{\text{CO}}_{{2}} } \right)$$

3 $${\text{Eb}} = {\text{ E}}_{{{\text{def}}}} + {\text{E}}_{{\text{i}}} + {\text{C}} = { }\sum {\text{E}}_{{{\text{def}}}} \left( {\text{i}} \right) + \sum \sum \Delta^{2} {\text{E}}\left( {{\text{ij}}} \right) + {\text{C}}$$

4 $${\text{E}}_{{{\text{def}}}} \left( {\text{i}} \right) \, = {\text{ E}}\left( {\text{i}} \right){-}{\text{E}}^{\prime } \left( {\text{i}} \right)$$

5 $$\Delta^{{2}} {\text{E}}\left( {{\text{ij}}} \right) \, = {\text{ E}}\left( {{\text{ij}}} \right){-}{\text{E}}^{\prime } \left( {\text{i}} \right){-}{\text{E}}^{\prime } \left( {\text{j}} \right)$$

Finally, the density changes that take place in the systems due to the fixation of the CO_2_ molecules, have been analyzed using the Electron Density Shift (EDS) method^56,57^. In a XY complex, the EDS is calculated as the difference between the electron density of the complex and the sum of the isolated monomers in the geometry of the complex (Eq. 6). This method enables to localize regions of space where the density increases by fixation of the CO_2_ (EDS > 0) and in the contrary regions where the density decreases (EDS < 0).

6 $${\text{EDS}}\left( {\text{r}} \right) = {\uprho }_{{{\text{XY}}}} \left( {\text{r}} \right) - {\uprho }_{{\text{X}}} \left( {\text{r}} \right) - {\uprho }_{{\text{Y}}} \left( {\text{r}} \right)$$

## Results and discussion

This section has been divided in four subsections: (3.1) the characteristics of the isolated B_3_P_3_-NG will be discussed, (3.2) the sequential interaction and incorporation of CO_2_ to the B_3_P_3_-NG molecule, (3.3) the cooperativity analysis of the process described in "Sequential interaction and capture of co2 by b3p3-ng." Section, and (3.4) where three CO_2_ molecules will be present along the whole reaction process of interaction and incorporation. The complex and adduct formation of the third molecule is common to the two mechanisms discussed in 3.2 and 3.4, and consequently the global results are the same.

The nomenclature used for the stationary points between B_3_P_3_-NG and one or more CO_2_ molecules uses the following formalism: B_3_P_3_-NG:*m*CO_2_ will indicate a non-covalent complex of B_3_P_3_-NG and *m* CO_2_ molecules. *n*CO_2_-(B_3_P_3_-NG):*m*CO_2_ will be used for the non-covalent complex formed between *m* CO_2_ molecules and the B_3_P_3_-NG having already *n* CO_2_ molecules covalently bonded on its surface. *n*CO_2_-(B_3_P_3_-NG)/CO_2_:*m*CO_2_ will specify the TS between the non-covalent complex and the adduct of a molecule of CO_2_ with m additional non-covalent molecules of CO_2_ interacting to B_3_P_3_-NG and having *n* CO_2_ already attached to its surface. Finally *n*CO_2_-(B_3_P_3_-NG) correspond to the adduct formed by the B_3_P_3_-NG and *n* CO_2_ molecules.

### Isolated B_3_P_3_-doped nanographene

**Figure 2:**
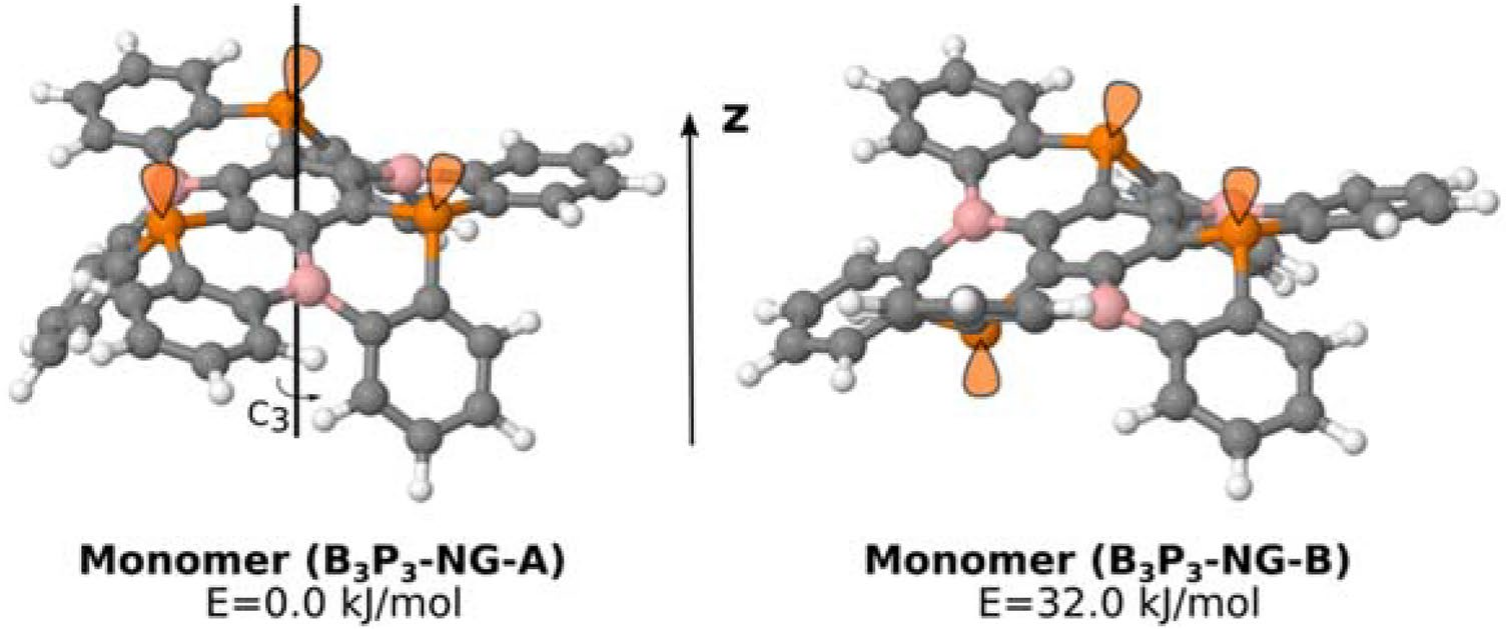
Geometry of the two energy minima found for the B_3_P_3_-NG monomer optimized at M06-2X/6-31+G* computational level. In orange, schematic orientation of the phosphorus lone pairs.

The conformational search of B_3_P_3_-NG provides two minima (Fig. 2). The most stable one, B_3_P_3_-NG-A, shows a *C*_*3*_ symmetry with the three lone pairs of the P atoms pointing towards the same direction (+ z in Fig. 2), while B_3_P_3_-NG-B with *C*_*1*_ symmetry shows two lone pairs of the P atoms in one direction and the other one in the opposite direction. The energy difference between these two structures is 32.0 kJ mol^−1^. The barrier to convert B_3_P_3_-NG-A to B_3_P_3_-NG-B has been calculated to be 72.4 kJ mol^−1^. Based on the energy difference of the two conformers, a Boltzmann distribution indicates that only B_3_P_3_-NG-A will be present at room temperature (n_a_/n_b_ > 10^5^). In the rest of the article only the B_3_P_3_-NG-A conformation will be considered and simply labelled as B_3_P_3_-NG.

**Figure 3:**
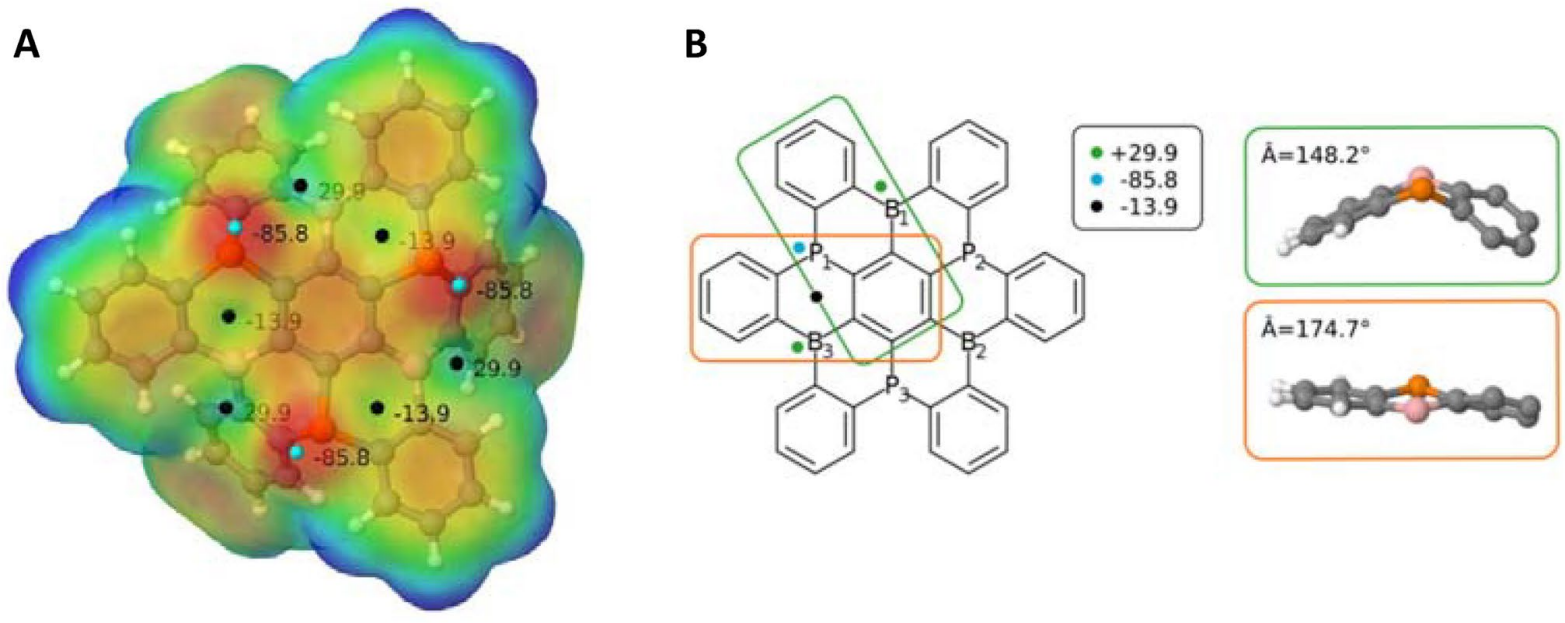
(**A**) MEP of the + z face of monomer B_3_P_3_-NG on the 0.001 a.u. electronic density isosurface. Cyan small points indicate the minima of the MEP, and black points the corresponding maxima. The MEP values are given in kJ mol^–1^, the color range used is [− 79.0; + 66.0] kJ mol^−1^, M06-2X/6-311++G(3df,3pd)//M06-2X/6-31+G* computations. (**B**) Details of the two types of C_4_BP rings in B_3_P_3_-NG.

The molecular electrostatic potential (MEP) of B_3_P_3_-NG has been calculated (Fig. 3) to identify the potential regions were the CO_2_ molecules could interact. We should emphasize that, in general, when a CO_2_ molecule is about to interact with a FLP, it first forms an electrostatic complex. Thus, the MEP can give relevant information about preferential positions in the formation of the complex.

Since the molecule presents a *C*_*3*_ symmetry, the characteristics of the MEP are repeated by a 120º rotation. There are two types of six-membered rings with P/B atom pairs with respect to the MEP:

- Those with a − 13.9 kJ mol^−1^ maximum above the center of the ring in the + z direction. This situation corresponds to those six-membered ring with P/B atoms that are almost coplanar with the two surrounding aromatic rings showing an angle between the centroids of the three rings of 174.7° (Fig. 3b)
- Those that do not present a negative maximum between the phosphorus and the boron atoms. In this case, the six-membered ring with P/B atoms is bent with respect to the two adjacent aromatic rings and the angle between the centroids of the three rings is 148.2° in Fig. 3b.

As expected, the most favorable position for the interaction of the first CO_2_ molecule will be the FLPs that do not have a negative maximum between the Lewis acid and base. This can be at the origin of a repulsive electrostatic interaction with CO_2_, with a less favorable formation of the pre-reactive complex. The − z face of B_3_P_3_-NG (Fig. 2) was not represented as no interesting extrema are located on that face due to the already mentioned orientation of the phosphorus lone pairs pointing towards + z.

### Sequential interaction and capture of CO_2_ by B_3_P_3_-NG

**Figure 4:**
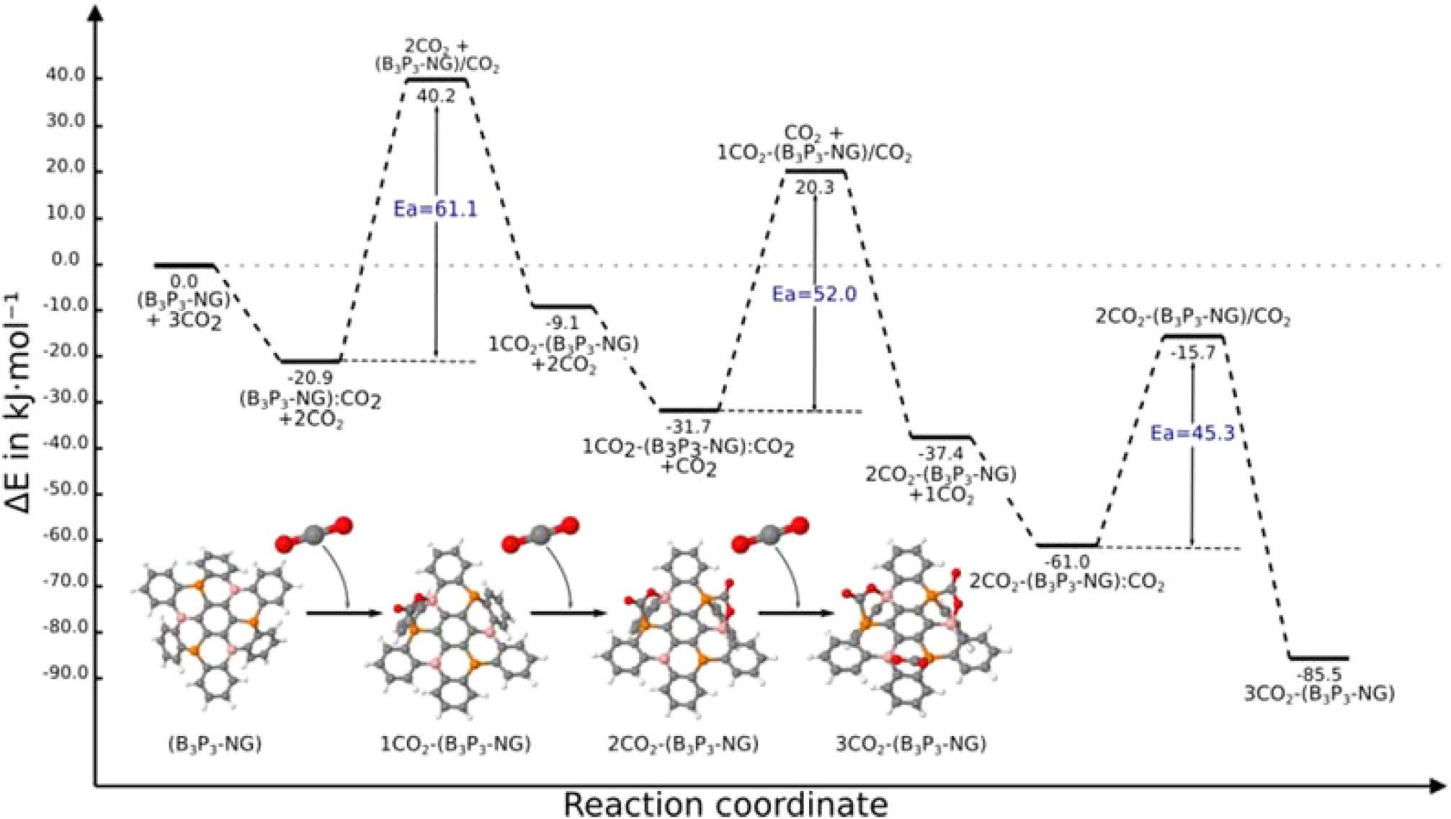
Reaction profile of the sequential capture of three CO_2_ molecules by B_3_P_3_-NG. The energies are in kJ mol^−1^ and correspond to M06-2X/6-311++G(3df,3pd)//M06-2X/6-31+G* calculations.

As pointed out above, the B_3_P_3_-NG chosen structure for this study is symmetric, and provides three degenerate favourable positions. In other words, the first adduct can be formed by attacking, without distinction, the pairs P1/B1, P2/B2 or P3/B3 (Fig. 3b).The sequential complex formation and incorporation of CO_2_ to the B_3_P_3_-NG molecule will be discussed below and the energy profile of the process is shown in Fig. 4.

**Figure 5:**
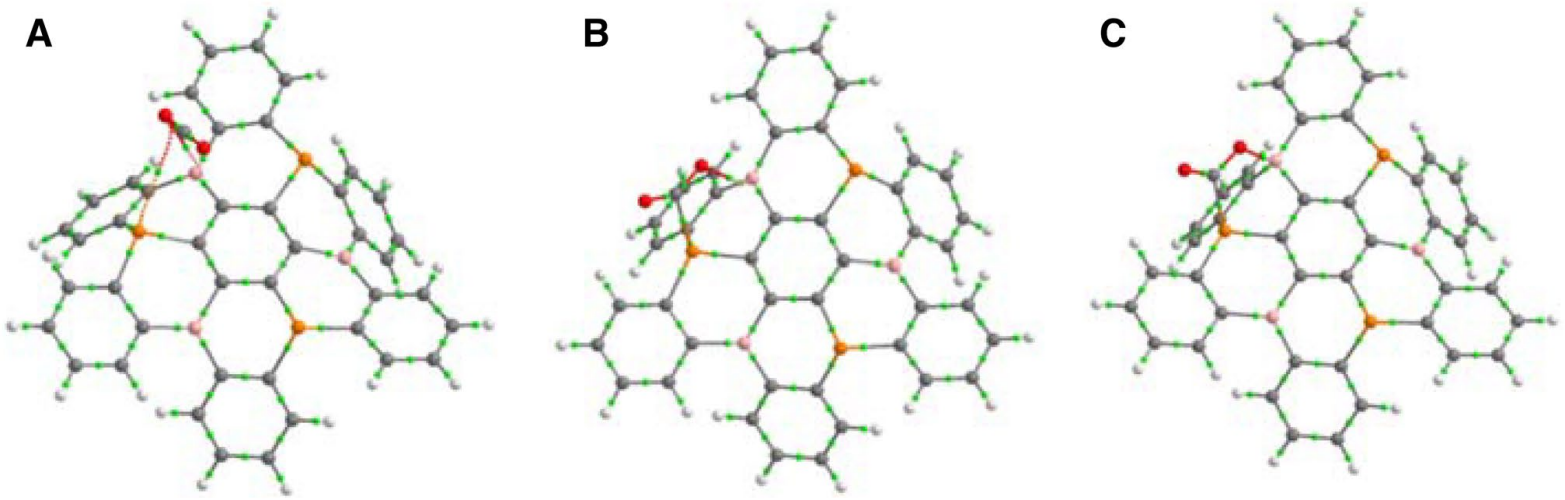
Molecular graph of the (**A**) (B_3_P_3_-NG):CO_2_ , (**B**) (B_3_P_3_-NG)/CO_2_ and (**C**) 1CO_2_-(B_3_P_3_-NG) of the first CO_2_ capture by B_3_P_3_-NG. Bond critical points (BCP) are shown in green.

**Figure 6:**
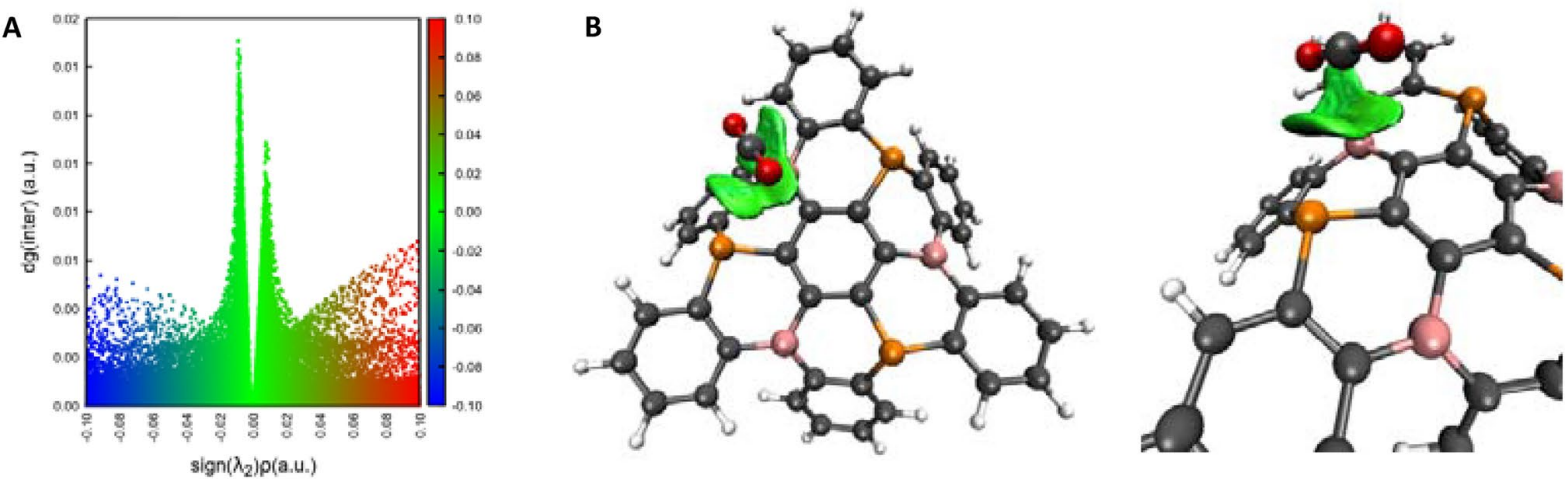
IGMPlot results of (B_3_P_3_-NG):CO_2_. (**A**) 2D plot of the δg as a function of the sign(λ_2_). Attractive interactions, Van der Waals regions, and repulsive interactions are shown in blue, green and red colours respecitvely. (**B**) Interaction surface of the CO_2_ molecule with the π system of B_3_P_3_-NG.

First, as it is often the case when a CO_2_ molecule is interacting with a FLP, a pre-reactive complex is formed, in the present case with a binding energy of –20.9 kJ mol^−1^. In general, the complex is stabilized by electrostatic interactions. Indeed, as it can be observed in Fig. 5A, the CO_2_ molecule is not activated. It remains in a geometry close to the one it adopts when isolated in vacuum, with C-O bonds around 1.17 Å and a O–C–O angle around 179°. As indicated in the molecular graph of the (B_3_P_3_-NG):CO_2_, Fig. 5A, only one oxygen atom in CO_2_ is interacting with the acidic and basic center of B_3_P_3_-NG. A bond path is observed between the oxygen and the boron atom, as well as between the same oxygen and the phosphorus atom. The density at the BCP for these interactions is 0.009 and 0.008 a.u. respectively. As observed in the case of the dibenzophosphaborine^39^, CO_2_ is tilted toward the central phenyl ring, due to a π-π stacking between the π-system of the B_3_P_3_-NG and the C=O double bond. The IGMPlot software was used to characterize the interaction between the B_3_P_3_-NG and the CO_2_ molecules in the complex. The interaction surface between B_3_P_3_-NG (Fragment 1) and CO_2_ (Fragment 2) is depicted in Fig. 6, using a density cutoff of 0.01 a.u. It can be observed that the orientation of the CO_2_ molecule is linked with a maximization of the π-π staking between the π-system of the P/B ring and the double bonds of CO_2_. These interactions appear for a density of 0.008 a.u.. The attractive interaction [sign(λ_2_) ρ = − 0.008 a.u.], has a larger maximum value, hence a higher contribution as compared to the repulsive interaction [sign(λ_2_) ρ =  + 0.008 a.u.].

Also a non-covalent complex between the B1/P2 couple of B_3_P_3_-NG and CO_2_ has been obtained with a binding energy of –20.7 kJ mol^−1^ (almost the same energy as B_3_P_3_-NG:1CO_2_). However all attempts to optimize the corresponding adduct in that position failed, returning back to the complex in the optimization process. The P–C and B–O distances in this less favorable B1/P2 complex are longer than the ones in the complex with the B1/P1 pair (d(P2–C) = 3.979 Å vs. d(P1–C) = 3.332 Å; d(B1–O) = 2.955 Å vs. d(B1–O) = 2.886 Å).

The transformation of the first pre-reactive complex into the adduct proceeds through a TS with an energy barrier of + 40.2 kJ mol^−1^ (Fig. 5B). The adduct has a relative energy of − 9.1 kJ mol^−1^ with respect to the isolated B_3_P_3_-NG and CO_2_ molecules (Fig. 5C). It is more stable than the entrance channel (B_3_P_3_-NG + CO_2_) but less stable than the complex. This behavior is common in the interaction between CO_2_ and FLPs^31,39,58^.

The formation of the adduct breaks the symmetry of the system. The remaining P/B couples are no longer equivalent by symmetry. For example, in the case of the B2/P2 pair the two surrounding aromatic rings show an angle between the centroids of the three rings of 146.4° ; this angle is 151.9° in the case of the P3/B3 pair. The MEP of the P/B pairs is also different. For the P2/B2 pair (Fig. S1 of the Supporting Material), the extrema are respectively − 111.6 and + 31.8 kJ mol^−1^. For the P3/B3 pair, the extrema are − 78.5 and + 72.5 kJ mol^−1^. The adduct in both cases are identical but the differences can influence the stability of the TSs. A priori, it is not possible to predict which FLP will provide the smaller barrier to fix the new CO_2_ molecule. Indeed, P2/B2 has a more adequate geometry, but B2 is less electrophilic than B3. However, P2 is more nucleophilic than P3 based on the MEP. The TS which enables the fixation of the CO_2_ molecule on the P2/B2 FLP has a relative energy of + 20.3 kJ mol^−1^ and the one where CO_2_ interacts with P3/B3 is approximately 5 kJ mol^−1^ higher in energy. Thus, the fixation of the new CO_2_ molecule is easier on the P2/B2 pair for kinetic reasons. Statistically, both reactions can take place, but in this work we will consider only the most probable based on its lower barrier.

Finally, the last CO_2_ molecule can be fixed on the remaining P3/B3 pair. The TS 2CO_2_-(B_3_P_3_-NG)/CO_2_ has a relative energy of − 15.7 kJ mol^−1^ with respect to the isolated systems and the 3CO_2_-(B_3_P_3_-NG) has a relative energy of − 85.5 kJ mol^−1^. The striking point with this last capture is that the TS is more stable than the entrance channel.

In order to get more insights into the reaction, the enthalpies and free energies of the successive captures were calculated (Fig. S2). As expected, the entropic term, –TΔS, has a large positive contribution, since two molecules react to form only one. For that reason, at room temperature, the captures, even if the last two are exothermic, will not be spontaneous. One can however observe a cooperative effect as the ΔΔG reduces when increasing the number of CO_2_ molecules captured (28.6; 16.6; 3.2 kJ mol^−1^).

### Understanding the cooperativity along the reaction

The presence of a cooperative effect is clear, as observed in Fig. 4. On one hand the adducts are more stable, but strikingly the activation barriers are reduced with the number of captured CO_2_ molecules.

#### Stability of the adducts

The first indication of cooperativity is that the larger the number of CO_2_ molecules on the B_3_P_3_-NG structure, the more stable the obtained adducts are. The increase in the interaction energy and the presence of a cooperative effect is even more obvious by using the decomposition scheme proposed in the Computational Methods section. A priori, the increase in stability can be related to an increase of the interaction energy between the CO_2_ and the B_3_P_3_-NG, and/or a decrease of the deformation energy needed to form the adduct. The interaction energy in the adduct per CO_2_ molecule (E_i_/*n*CO_2_), ranges from − 415.3 kJ mol^−1^ in 1CO_2_-(B_3_P_3_-NG) to − 437.5 kJ mol^−1^ in 2CO_2_-(B_3_P_3_-NG) adduct/complex and − 457.9 kJ mol^−1^ in the 3CO_2_-(B_3_P_3_-NG) one (Table 1). The deformation energy of B_3_P_3_-NG per CO_2_ molecule increases slightly with the number of CO_2_ molecules: 151.8 kJ mol^−1^, 155.1 kJ mol^−1^ and 155.3 kJ mol^−1^ for 1, 2 and 3 CO_2_ molecules, respectively. Thus, it seems that the increase in adduct stability is mainly due to an increase of interaction energy between CO_2_ and the B_3_P_3_-NG moiety. It can be realized that indeed, the interaction energy by CO_2_ increases, but that the total increase in stability is due to the presence of a cooperative energy, C. In other words, in the 2CO_2_-(B_3_P_3_-NG) adduct, the interaction energy of CO_2_(1) and CO_2_(2) is larger than the one of CO_2_(1) in the 1CO_2_-(B_3_P_3_-NG) adduct. However the presence of CO_2_(1) and CO_2_(2) at the same time on the B_3_P_3_-NG induces an extra interaction of –24.1 kJ mol^–1^, explaining the extra stabilization of the adduct.

**Table 1:** Binding energy (Eb), total and individual deformation energies (E_def_), total interaction energy (E_i_), interaction energy for each individual contact with a CO_2_ molecule (Ei_B3P3-NG-CO2(n)_), mean interaction energy per CO_2_ molecule (E_i_/*n*CO_2_), cooperative energy (C) in kJ mol^−1^ for the different adducts obtained (M06-2X/6-311++G(3df,3pd)//M06-2X/6-31+G*).

	1CO_2_-(B_3_P_3_-NG)	2CO_2_-(B_3_P_3_-NG)	3CO_2_-(B_3_P_3_-NG)
E_b_			
E_def_	+ 406.2	+ 837.6	+ 1288.2
E_i_	− 415.3	− 850.9	− 293.3
C		− 24.1	− 80.4
Total	− 9.1	− 37.4	− 85.5
E_def_			
E_def_ (B_3_P_3_-NG)	+ 151.8	+ 310.1	+ 465.9
E_def_ (CO_2_(1))	+ 254.4	+ 263.0	+ 274.1
E_def_ (CO_2_(2))		+ 264.5	+ 274.1
E_def_ (CO_2_(3))			+ 274.1
E_i_			
E_i_/nCO_2_	− 415.3	− 437.5	− 457.9
E_i_ (B_3_P_3_-NG-CO_2_(1))	− 415.3	− 420.6	− 431.2
E_i_ (B_3_P_3_-NG-CO_2_(2))		− 430.4	− 431.2
E_i_ (B_3_P_3_-NG-CO_2_(3))			− 431.2

The variation in the interaction energy can also be analyzed from a geometrical point of view by looking at the B–O and P–C bonds of the first fixed CO_2_ molecule when increasing the number of CO_2_ molecules. First, it can be observed that the P1–C distance increases a little bit when adding more CO_2_ molecules (Table 2). The total change is 0.006 Å. The B1–O bond is more affected by the fixation of new CO_2_ molecules as its distance decreases. It shortens by 0.021 Å, from 1.581 Å in 1CO_2_-(B_3_P_3_-NG), to 1.574 Å in 2CO_2_-(B_3_P_3_-NG), to 1.560 Å in 3CO_2_-(B_3_P_3_-NG) (Table 2). Looking at the IBSI and at the density at the different BCP, it can be observed that the properties of the P–C bonds do not change significantly with the number of attached CO_2_ molecules. The strength and the density of this bond are not really influenced by the fixation of new CO_2_ molecules. On the contrary, it can be observed that the B–O bond gets stronger when increasing the number of CO_2_ fixed. In other words, it seems that the increase of stability observed is linked with a strengthening of the B-O interaction.

**Table 2:** Bond distances (Å), intrinsic bond strength index (IBSI) in a.u. and density at the Bond Critical Points (BCP) in a.u. of the P1-C and O-B1 bond in the different adducts.

	1CO_2_-(B_3_P_3_-NG)			2CO_2_-(B_3_P_3_-NG)			3CO_2_-(B_3_P_3_-NG)		
	Bond distance	IBSI	ρ_BCP_	Bond distance (Å)	IBSI (a.u.)	ρ_BCP_ (a.u.)	Bond distance (Å)	IBSI (a.u.)	ρ_BCP_ (a.u.)
P1–C bond	1.901	0.458	0.160	1.903	0.454	0.159	1.907	0.464	0.158
O–B1 bond	1.581	0.572	0.112	1.574	0.582	0.115	1.560	0.618	0.120

The IBSI were obtained using IGMPlot, and the BCP were localized with AIMAll at M06-2X/6-311+ +G(3df,3pd)//M06-2X/6-31+G* computational level.

#### The stabilization of the TSs

**Figure 7:**
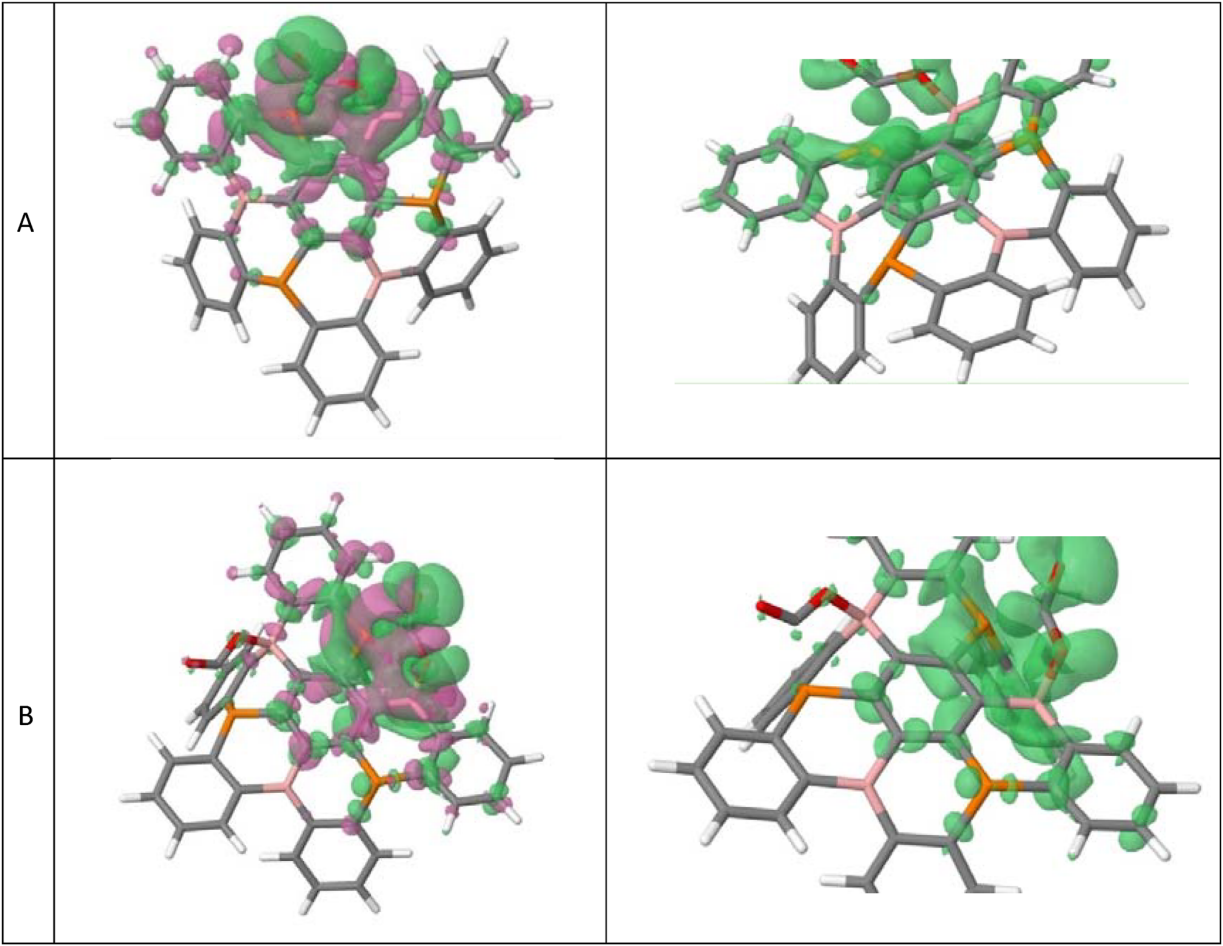
Electron Density Shift (EDS) plots of (**A**) 1CO_2_-(B_3_P_3_-NG) and (**B**) 2CO_2_-(B_3_P_3_-NG) . In magenta color the region of electron density decrease and in green the region of electron density increase. An isovalue of 0.001 a.u. was used for the plot.

As it can be observed in Fig. 4, the successive fixation of CO_2_ molecules is accompanied by a decrease of the activation barrier. In a previous paper, we observed that during the capture of CO_2_ by a P/B cyclic FLP, the energy of the TS can be influenced by the basicity of the phosphorus atom^39^. In order to see if the differences in activation energy are related to the increase of phosphorus basicity, we computed Electron Density Shift (EDS) plots of the 1CO_2_-(B_3_P_3_-NG) and 2CO_2_-(B_3_P_3_-NG) adducts, as shown in Fig. 7.

As it can be observed, the fixation of the first CO_2_ molecule in P1/B1 induces a shift of the density toward the phosphorus atoms (P2) while the rest of the system is not influenced by CO_2_ addition (Fig. 7A). The same behavior is observed when 2CO_2_-(B_3_P_3_-NG) is formed (Fig. 7B).

### Simultaneous reaction of three CO_2_ molecules with B_3_P_3_-NG

**Figure 8:**
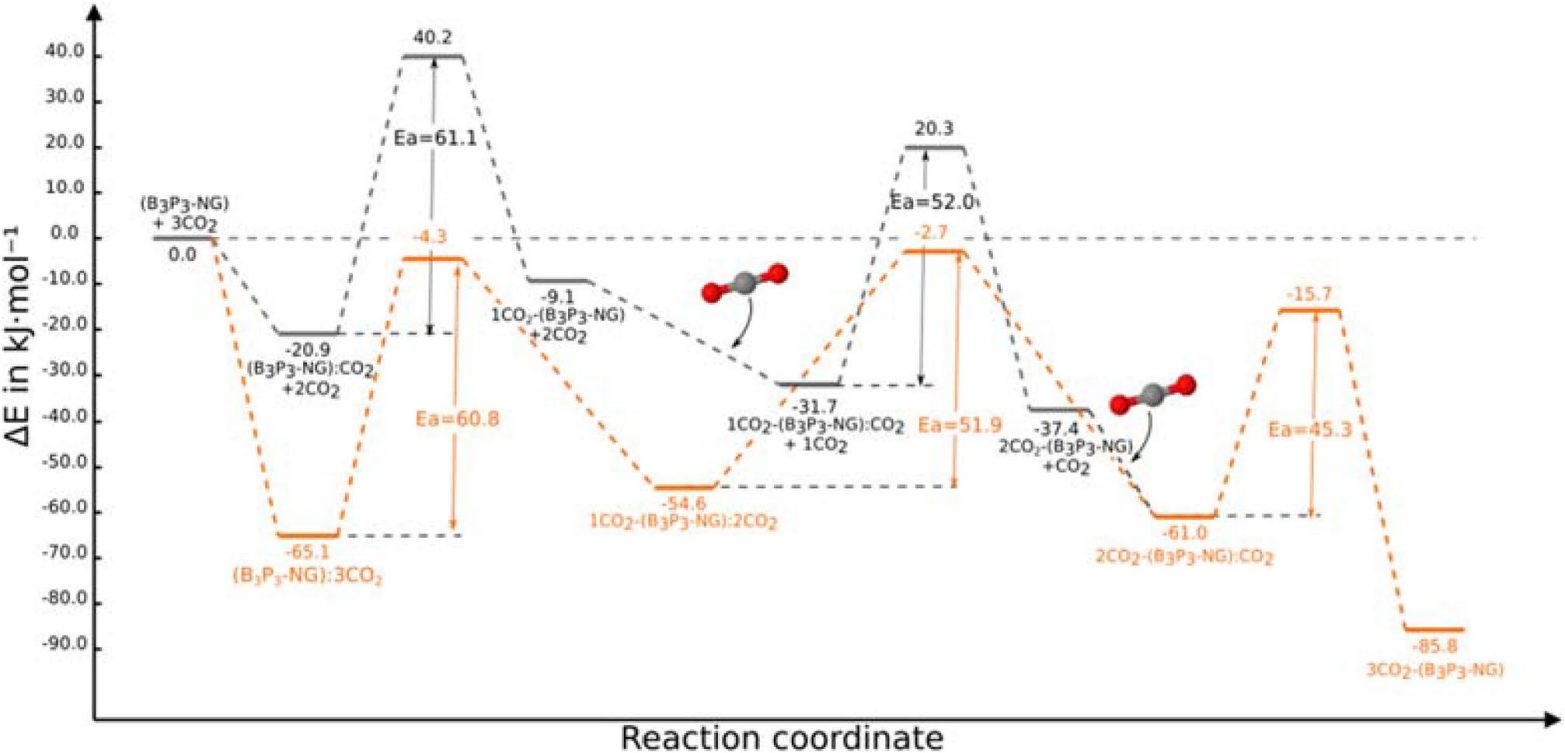
Comparison of the reaction profiles obtained by sequential addition of CO_2_ molecules (black), and with 3 CO_2_ molecules along the reaction profile (orange). The energies are in kJ mol^–1^ and calculated at M06-2X/6-311+ +G(3df,3pd)//M06-2X/6-31+G* computational level.

Another alternative is B_3_P_3_-NG surrounded by CO_2_ molecules as the addition reactions proceed. These conditions can occur in a CO_2_ atmosphere or CO_2_ supercritical. Thus, we consider the presence of three CO_2_ molecules along the reaction coordinate, as depicted in Fig. 8.

We should emphasize that in this new reaction path (Fig. 8, orange curve), the pre-reactive complexes of the next step and adducts from the previous step are the same stationary points. For this reason the new path presents only 7 stationary points instead of 9, as describe above in "Isolated B3P3-doped nanographene" Section.

The first stationary point, (B_3_P_3_-NG):3CO_2_, has a relative energy of − 65.1 kJ mol^−1^ while the (B_3_P_3_-NG):CO_2_ adduct/complex is − 20.9 kJ mol^−1^. In other words, the presence of the two extra CO_2_ molecules in (B_3_P_3_-NG):3CO_2_ produces a stabilization of − 44.2 kJ mol^−1^, more than twice the energy of (B_3_P_3_-NG):CO_2_, hence the presence of a cooperativity effect; otherwise the energy of (B_3_P_3_-NG):3CO_2_ would have been three times the energy of (B_3_P_3_-NG):CO_2_.

We also used the IGMPlot software in order to study this cooperative effect. By integrating the peaks corresponding to the attractive weak interactions, we obtained a value of 0.09 a.u. for (B_3_P_3_-NG):CO_2_ with a rise of 0.36 a.u. for (B_3_P_3_-NG):3CO_2_, more than three times larger, confirming the cooperativity effect as more CO_2_ molecules are added to the system.

In this new reaction profile one can observe that all TS are more stable as compared to the entrance channel. The relative energy of the first TS ranges from + 40.2 to − 4.3 kJ mol^−1^ (ΔE = 44.5 kJ mol^−1^), and the second TS ranges from + 20.3 to –2.7 kJ mol^−1^ (ΔE = 23.0 kJ mol^−1^). It can be pointed out that the activation energies are similar in both reaction paths (61.1 vs. 60.8 kJ mol^−1^ and 52.0 vs. 51.9 kJ mol^−1^). This fact is due to the similar stabilization of adducts and TSs.

## Conclusion

The capture of CO_2_ molecules by the B_3_P_3_-NG compound was studied by means of DFT computational methods. Two potential mechanisms have been studied: (i) the interaction between B_3_P_3_-NG and CO_2_ molecules and adduct formation is done sequentially, and (ii) three CO_2_ molecules interact simultaneously with the B_3_P_3_-NG compound along the reaction coordinate.

The main conclusions are:

- The capture of CO_2_ by B_3_P_3_-NG presents a cooperative effect.
- The increase in number of CO_2_ molecules fixed on the B_3_P_3_-NG surface stabilizes the respective adducts due to an increase of the boron acidity, and then an increase of the boron-oxygen interaction.
- The decrease of the activation barriers with the number of CO_2_ molecules is due to a basicity increase of the phosphorus.
- The cooperative effects observed in this system are linked to the π delocalization of the system. The modifications imposed by the fixation of a new CO_2_ molecule are compensated by a π-electron reorganization, affecting the acidity and basicity of the boron and phosphorus atoms.
- The multi-capture can be enhanced by considering that several CO_2_ molecules are simultaneously in contact with the B_3_P_3_-NG surface.

## Supplementary Information

Supplementary Information.

## Data Availability

All data generated or analysed during this study are included in this published article and its supplementary information files.
