# Supplementary material for: Reactivity of a model of B3P3-doped nanographene with up to three CO2 molecules
Source: Sci Rep. 2023 Feb 10;13:2407. doi: 10.1038/s41598-023-29336-y (PMC9918725; doi:10.1038/s41598-023-29336-y)
Supplement: Supplementary file 1 — Supplementary Information. [file 41598_2023_29336_MOESM1_ESM.docx]

**SUPPORTING INFORMATION**

Reactivity of a model of B_3_P_3_-doped nanographene with up to three CO_2_ molecules

Maxime Ferrer, ^a,b^ Ibon Alkorta, ^a*^ JoseElguero, ^a^ Josep M. Oliva-Enrich ^c^

^a^ Instituto de Química Médica (CSIC). Juan de la Cierva, 3, E-28006 Madrid, Spain

^b^ PhD Program in Theoretical Chemistry and Computational Modeling, Doctoral School, Universidad Autónoma de Madrid, 28049 Madrid, Spain

^c^ Instituto de Química-FísicaRocasolano (CSIC). Serrano, 119, E-28006 Madrid, Spain

**Index**

| Pg. S2-S5 | **Table S1:** Geometries, energies (in Hartree) and Cartesian coordinates (in Å) of the monomers in vacuum. Level of theory : M06-2X / 6-31+G* |
| --- | --- |
| Pg. S3-S11 | **Table S2:** Geometries, energies (in Hartree) and Cartesian coordinates (in Å) of the complexes in vacuum. Level of theory : M06-2X/6-31+G* |
| Pg. S12-S19 | **Table S3:** Geometries, energies (in Hartree) and Cartesian coordinates (in Å) of the transition states in vacuum. Level of theory : M06-2X/6-31+G* |
| Pg. S19-S25 | **Table S4:** Geometries, energies (in Hartree) and Cartesian coordinates (in Å) of the adducts in vacuum. Level of theory : M06-2X/6-31+G* |
| Pg. S25-27 | **Table S5 :** Comparison of the M06-2X/6-31+G* and M06-2X/6-311++G(3df,3dp) level of theories for the optimization (RMSD). |
| Pg. S28 | **Figure S1 :** Molecular Electrostatic Potential surface of the 1CO_2_-(B3P3-NG) calculated on the 0.001a.u. electron density isosurface. Level of Theory : M06-2X/6-311++G(3df,3pd). |
| Pg. S29 | **Figure S2.** Enthalpies (green) and free energies (black) of the successive CO_2_ captures at 298K, calculated using the (M06‐2X/6‐311++G(3df,3dp)//M06‐2X/6‐31+G*) electronic energy and the M06‐2X/6‐31+G* thermal and entropic corrections. In each CO_2_ capture, the enthalpy and free energy of the entrance channel has been defined as 0.0 kJ mol^-1^. |

**Table S1:** Geometries, energies (in Hartree) and Cartesian coordinates (in Å) of the monomers in vacuum. Level of theory : M06-2X / 6-31+G*

| 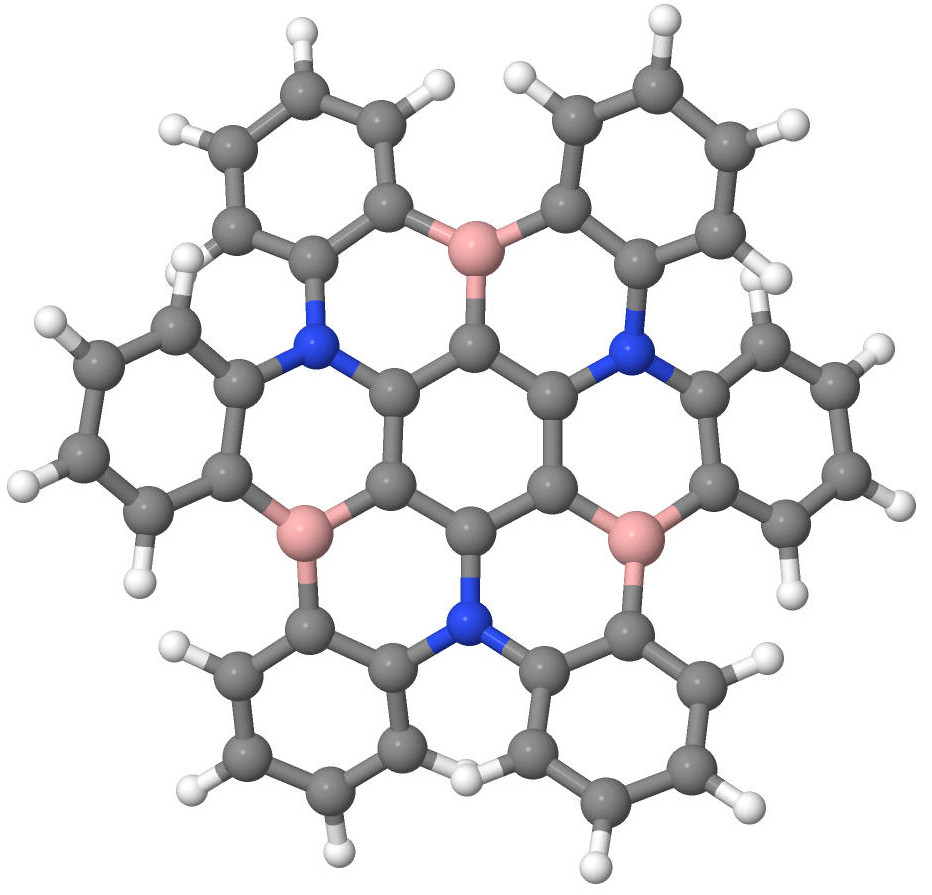 | B3N3-NG E=-1853.241025H FreqIm=0   \| C \| -5.927034 \| -0.473092 \| 1.531300 \| \| --- \| --- \| --- \| --- \| \| C \| -3.611841 \| 0.730305 \| 0.489325 \| \| C \| -3.752970 \| -0.673760 \| 0.430655 \| \| C \| -4.602891 \| 1.508895 \| 1.113130 \| \| C \| -5.746693 \| 0.911755 \| 1.617759 \| \| N \| 0.033193 \| -2.792410 \| 0.000045 \| \| N \| -2.434872 \| 1.367556 \| 0.000186 \| \| C \| -1.227616 \| -0.728350 \| -0.000004 \| \| C \| -2.390078 \| -2.970859 \| -0.431018 \| \| C \| 0.016706 \| -1.398200 \| -0.000015 \| \| C \| -3.684368 \| 3.144963 \| -1.112353 \| \| H \| -4.525315 \| 2.469566 \| -1.223819 \| \| C \| -2.503412 \| 2.704182 \| -0.488990 \| \| C \| -1.219189 \| 0.684803 \| -0.000030 \| \| C \| -3.452952 \| -3.729701 \| -0.959782 \| \| H \| -4.450592 \| -3.298369 \| -0.952500 \| \| C \| -1.377934 \| 3.555463 \| -0.431046 \| \| C \| -1.090155 \| -3.520011 \| -0.489040 \| \| C \| -0.881577 \| -4.763166 \| -1.112408 \| \| H \| 0.123776 \| -5.153819 \| -1.223968 \| \| C \| -3.255512 \| -4.975429 \| -1.531484 \| \| B \| -2.522127 \| -1.496365 \| -0.000149 \| \| C \| -3.770095 \| 4.432249 \| -1.617337 \| \| H \| -4.687264 \| 4.748103 \| -2.106266 \| \| C \| -2.681482 \| 5.307076 \| -1.531532 \| \| C \| -1.503852 \| 4.855382 \| -0.959779 \| \| H \| -0.631679 \| 5.503958 \| -0.952448 \| \| C \| -1.953621 \| -5.480953 \| -1.617293 \| \| H \| -1.768693 \| -6.433214 \| -2.106196 \| \| B \| -0.034804 \| 2.932646 \| -0.000384 \| \| C \| -0.016899 \| 1.427538 \| -0.000312 \| \| C \| 5.936781 \| -0.332037 \| -1.531603 \| \| C \| 3.593653 \| 0.815798 \| -0.489490 \| \| C \| 3.768066 \| -0.584549 \| -0.431033 \| \| C \| 4.956714 \| -1.125739 \| -0.959597 \| \| H \| 5.082123 \| -2.205365 \| -0.951881 \| \| C \| 4.565904 \| 1.617801 \| -1.113217 \| \| H \| 4.401486 \| 2.683712 \| -1.225289 \| \| C \| 5.723563 \| 1.048145 \| -1.617931 \| \| H \| 6.455709 \| 1.684247 \| -2.107170 \| \| N \| 2.401891 \| 1.425039 \| -0.000461 \| \| C \| 1.244788 \| -0.698931 \| -0.000081 \| \| C \| 2.459986 \| -2.913257 \| 0.431143 \| \| C \| 3.608480 \| 3.231909 \| 1.112232 \| \| H \| 4.465319 \| 2.576864 \| 1.224085 \| \| C \| 2.438560 \| 2.762977 \| 0.488603 \| \| C \| 1.202721 \| 0.713641 \| -0.000313 \| \| C \| 3.540557 \| -3.646413 \| 0.960279 \| \| H \| 4.527707 \| -3.191617 \| 0.952852 \| \| C \| 1.293080 \| 3.587184 \| 0.430421 \| \| C \| 1.173455 \| -3.493079 \| 0.489383 \| \| C \| 0.994444 \| -4.740597 \| 1.113205 \| \| H \| -0.001324 \| -5.155042 \| 1.224832 \| \| C \| 3.372729 \| -4.896216 \| 1.532528 \| \| B \| 2.557148 \| -1.436092 \| 0.000033 \| \| C \| 3.663302 \| 4.520875 \| 1.617219 \| \| H \| 4.572502 \| 4.858555 \| 2.106441 \| \| C \| 2.554016 \| 5.369344 \| 1.531445 \| \| C \| 1.387692 \| 4.889594 \| 0.959484 \| \| H \| 0.500155 \| 5.516947 \| 0.952233 \| \| C \| 2.083234 \| -5.432509 \| 1.618433 \| \| H \| 1.920957 \| -6.388638 \| 2.107810 \| \| C \| -4.928378 \| -1.243166 \| 0.959195 \| \| H \| -5.028056 \| -2.325469 \| 0.951380 \| \| H \| -4.464025 \| 2.578445 \| 1.225239 \| \| H \| -6.493769 \| 1.530221 \| 2.107035 \| \| H \| -2.750023 \| 6.311137 \| -1.938767 \| \| H \| -6.819981 \| -0.937387 \| 1.938421 \| \| H \| -4.090863 \| -5.536682 \| -1.938723 \| \| H \| 4.221124 \| -5.437242 \| 1.940198 \| \| H \| 6.840531 \| -0.774960 \| -1.938697 \| \| H \| 2.598340 \| 6.374634 \| 1.939002 \| |
| --- | --- | --- | --- | --- | --- | --- | --- | --- | --- | --- | --- | --- | --- | --- | --- | --- | --- | --- | --- | --- | --- | --- | --- | --- | --- | --- | --- | --- | --- | --- | --- | --- | --- | --- | --- | --- | --- | --- | --- | --- | --- | --- | --- | --- | --- | --- | --- | --- | --- | --- | --- | --- | --- | --- | --- | --- | --- | --- | --- | --- | --- | --- | --- | --- | --- | --- | --- | --- | --- | --- | --- | --- | --- | --- | --- | --- | --- | --- | --- | --- | --- | --- | --- | --- | --- | --- | --- | --- | --- | --- | --- | --- | --- | --- | --- | --- | --- | --- | --- | --- | --- | --- | --- | --- | --- | --- | --- | --- | --- | --- | --- | --- | --- | --- | --- | --- | --- | --- | --- | --- | --- | --- | --- | --- | --- | --- | --- | --- | --- | --- | --- | --- | --- | --- | --- | --- | --- | --- | --- | --- | --- | --- | --- | --- | --- | --- | --- | --- | --- | --- | --- | --- | --- | --- | --- | --- | --- | --- | --- | --- | --- | --- | --- | --- | --- | --- | --- | --- | --- | --- | --- | --- | --- | --- | --- | --- | --- | --- | --- | --- | --- | --- | --- | --- | --- | --- | --- | --- | --- | --- | --- | --- | --- | --- | --- | --- | --- | --- | --- | --- | --- | --- | --- | --- | --- | --- | --- | --- | --- | --- | --- | --- | --- | --- | --- | --- | --- | --- | --- | --- | --- | --- | --- | --- | --- | --- | --- | --- | --- | --- | --- | --- | --- | --- | --- | --- | --- | --- | --- | --- | --- | --- | --- | --- | --- | --- | --- | --- | --- | --- | --- | --- | --- | --- | --- | --- | --- | --- | --- | --- | --- | --- | --- | --- | --- | --- | --- | --- | --- | --- | --- | --- | --- | --- | --- | --- | --- | --- | --- | --- | --- | --- | --- | --- | --- | --- | --- | --- | --- |
| 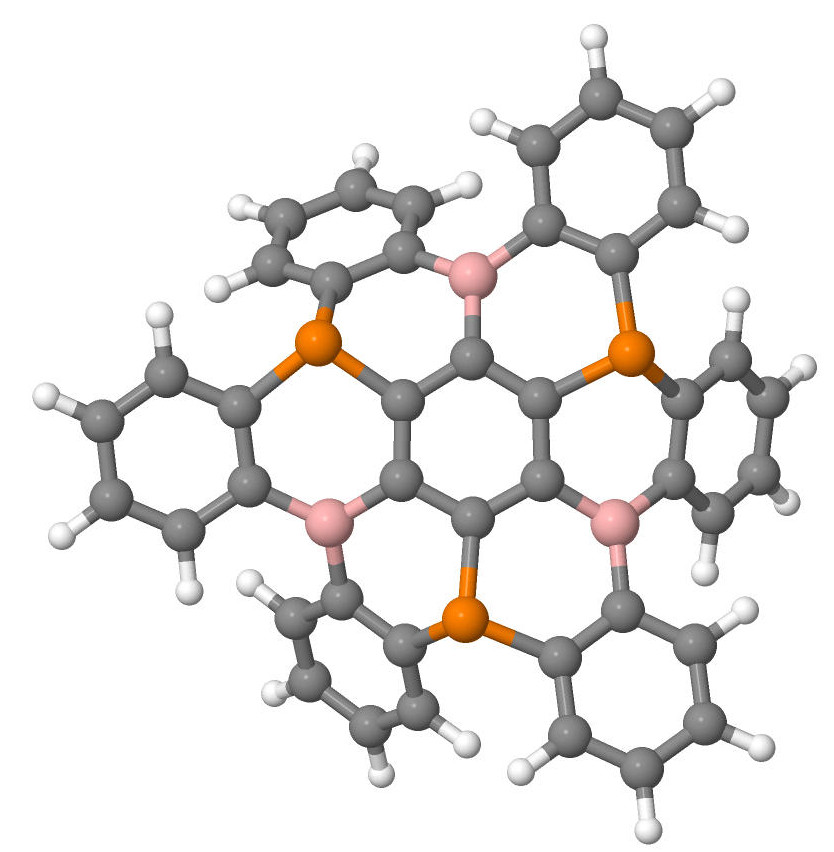 | B3P3-NG-A E=-2712.89675H FreqIm=0   \| C \| 5.159719 \| -0.091850 \| 2.368629 \| \| --- \| --- \| --- \| --- \| \| C \| 3.625435 \| -1.164260 \| 0.295214 \| \| C \| 3.624604 \| 0.238393 \| 0.491325 \| \| C \| 4.408558 \| -1.999278 \| 1.092196 \| \| C \| 5.172644 \| -1.463256 \| 2.128416 \| \| P \| 0.238814 \| 3.175436 \| -1.113362 \| \| P \| 2.629998 \| -1.794664 \| -1.115894 \| \| C \| 1.308421 \| 0.578714 \| -0.788643 \| \| C \| 2.975396 \| 2.706733 \| -0.501795 \| \| C \| 0.167174 \| 1.385239 \| -0.823058 \| \| C \| 3.193342 \| -4.480993 \| -0.958766 \| \| H \| 4.202557 \| -4.160065 \| -1.210722 \| \| C \| 2.186028 \| -3.524807 \| -0.765386 \| \| C \| 1.116040 \| -0.837302 \| -0.823455 \| \| C \| 4.304312 \| 3.176167 \| -0.465800 \| \| H \| 5.109220 \| 2.461207 \| -0.317422 \| \| C \| 0.856243 \| -3.929893 \| -0.499900 \| \| C \| 1.959185 \| 3.655856 \| -0.764389 \| \| C \| 2.283095 \| 5.006681 \| -0.956444 \| \| H \| 1.499927 \| 5.720411 \| -1.205855 \| \| C \| 4.619989 \| 4.518747 \| -0.637537 \| \| B \| 2.668364 \| 1.184424 \| -0.327978 \| \| C \| 2.912384 \| -5.839630 \| -0.866963 \| \| H \| 3.706210 \| -6.567782 \| -1.007820 \| \| C \| 1.602479 \| -6.260302 \| -0.635261 \| \| C \| 0.598054 \| -5.315391 \| -0.462133 \| \| H \| -0.423300 \| -5.654853 \| -0.311448 \| \| C \| 3.600227 \| 5.442923 \| -0.866279 \| \| H \| 3.833376 \| 6.494732 \| -1.005976 \| \| B \| -0.307898 \| -2.902518 \| -0.324997 \| \| C \| -0.153089 \| -1.422398 \| -0.787840 \| \| C \| -6.223191 \| 1.741347 \| -0.640751 \| \| C \| -4.145497 \| -0.131448 \| -0.766316 \| \| C \| -3.831979 \| 1.222944 \| -0.501826 \| \| C \| -4.903011 \| 2.139013 \| -0.466174 \| \| H \| -4.686642 \| 3.193541 \| -0.317213 \| \| C \| -5.476895 \| -0.526061 \| -0.961302 \| \| H \| -5.702893 \| -1.560876 \| -1.212436 \| \| C \| -6.513389 \| 0.396370 \| -0.871814 \| \| H \| -7.540670 \| 0.072694 \| -1.013714 \| \| P \| -2.868998 \| -1.381065 \| -1.114998 \| \| C \| -1.155319 \| 0.843702 \| -0.788184 \| \| C \| -2.019218 \| 3.018766 \| 0.493456 \| \| C \| -3.935360 \| -2.816178 \| 1.094900 \| \| H \| -4.862140 \| -2.270881 \| 0.939407 \| \| C \| -2.820606 \| -2.556697 \| 0.297588 \| \| C \| -1.283172 \| -0.547938 \| -0.823379 \| \| C \| -2.839307 \| 3.434066 \| 1.557373 \| \| H \| -3.751555 \| 2.882376 \| 1.768700 \| \| C \| -1.605212 \| -3.256779 \| 0.494944 \| \| C \| -0.804346 \| 3.720327 \| 0.298746 \| \| C \| -0.472073 \| 4.814455 \| 1.097485 \| \| H \| 0.464128 \| 5.343926 \| 0.943909 \| \| C \| -2.500273 \| 4.511602 \| 2.372339 \| \| B \| -2.360270 \| 1.718104 \| -0.326923 \| \| C \| -3.853304 \| -3.744372 \| 2.132451 \| \| H \| -4.719767 \| -3.922639 \| 2.763432 \| \| C \| -2.659048 \| -4.418160 \| 2.374014 \| \| C \| -1.555234 \| -4.172712 \| 1.560549 \| \| H \| -0.621116 \| -4.685611 \| 1.774036 \| \| C \| -1.318156 \| 5.207455 \| 2.133887 \| \| H \| -1.039198 \| 6.045946 \| 2.766158 \| \| C \| 4.394605 \| 0.740632 \| 1.555206 \| \| H \| 4.372476 \| 1.806285 \| 1.767552 \| \| H \| 4.399494 \| -3.074655 \| 0.937478 \| \| H \| 5.760900 \| -2.124038 \| 2.759272 \| \| H \| 5.655860 \| 4.843724 \| -0.606511 \| \| H \| 5.736919 \| 0.326685 \| 3.187835 \| \| H \| 1.365922 \| -7.319830 \| -0.602893 \| \| H \| -2.585214 \| -5.126186 \| 3.194202 \| \| H \| -7.022654 \| 2.475900 \| -0.610150 \| \| H \| -3.151555 \| 4.801773 \| 3.191516 \| |
| 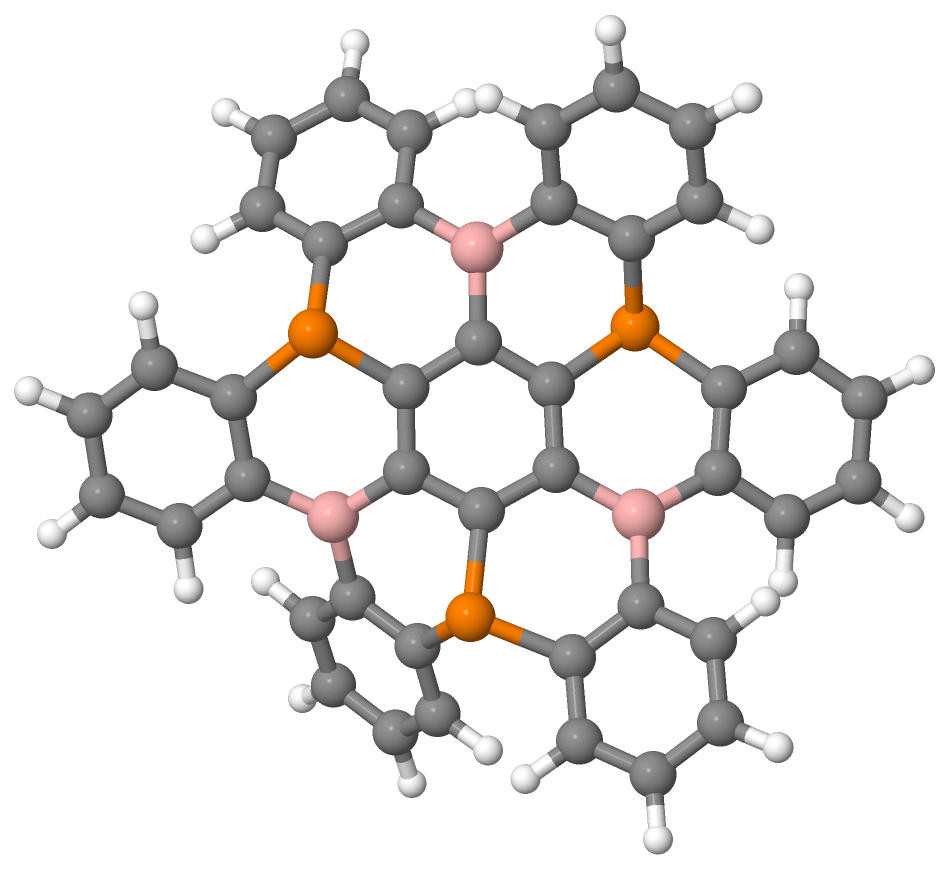 | B3P3-NG-B E=-2712.884556H FreqIm=0   \| C \| -0.868121 \| 5.410016 \| 2.078290 \| \| --- \| --- \| --- \| --- \| \| C \| 0.421548 \| 3.847492 \| 0.165801 \| \| C \| -0.956351 \| 3.626356 \| 0.408956 \| \| C \| 1.119227 \| 4.855043 \| 0.828190 \| \| C \| 0.473274 \| 5.634708 \| 1.785287 \| \| P \| -3.153131 \| -0.338111 \| -1.137133 \| \| P \| 1.189593 \| 2.796973 \| -1.127764 \| \| C \| -0.866961 \| 1.156369 \| -0.555039 \| \| C \| -3.259866 \| 2.431145 \| -0.523308 \| \| C \| -1.459191 \| -0.119324 \| -0.548432 \| \| C \| 3.709236 \| 3.834956 \| -1.351852 \| \| H \| 3.183116 \| 4.771587 \| -1.524606 \| \| C \| 2.990228 \| 2.703934 \| -0.936053 \| \| C \| 0.550919 \| 1.203381 \| -0.556208 \| \| C \| -3.972772 \| 3.645977 \| -0.547701 \| \| H \| -3.431525 \| 4.571657 \| -0.377900 \| \| C \| 3.643994 \| 1.460689 \| -0.742686 \| \| C \| -3.984247 \| 1.251274 \| -0.830306 \| \| C \| -5.350062 \| 1.320411 \| -1.138178 \| \| H \| -5.882081 \| 0.423491 \| -1.445431 \| \| C \| -5.334408 \| 3.704581 \| -0.812382 \| \| B \| -1.716167 \| 2.428235 \| -0.279806 \| \| C \| 5.070242 \| 3.760660 \| -1.609142 \| \| H \| 5.615325 \| 4.647241 \| -1.918792 \| \| C \| 5.709325 \| 2.523807 \| -1.542449 \| \| C \| 5.001256 \| 1.405425 \| -1.125195 \| \| H \| 5.511598 \| 0.448218 \| -1.111736 \| \| C \| -6.029410 \| 2.531417 \| -1.097354 \| \| H \| -7.091025 \| 2.563212 \| -1.323473 \| \| B \| 2.908182 \| 0.205529 \| -0.135027 \| \| C \| 1.357637 \| 0.092738 \| -0.239718 \| \| C \| -0.201121 \| -6.145853 \| -1.364100 \| \| C \| 0.882431 \| -3.910284 \| -0.085534 \| \| C \| -0.503911 \| -3.938159 \| -0.374416 \| \| C \| -1.019221 \| -5.076269 \| -1.012129 \| \| H \| -2.079569 \| -5.108532 \| -1.251659 \| \| C \| 1.701446 \| -4.985383 \| -0.425445 \| \| H \| 2.767327 \| -4.952187 \| -0.220770 \| \| C \| 1.159625 \| -6.093480 \| -1.074578 \| \| H \| 1.808726 \| -6.915790 \| -1.361158 \| \| P \| 1.427726 \| -2.483288 \| 0.920760 \| \| C \| -0.709944 \| -1.278006 \| -0.248930 \| \| C \| -2.911576 \| -2.734619 \| 0.215885 \| \| C \| 4.013076 \| -3.204882 \| 1.447573 \| \| H \| 3.577300 \| -4.170231 \| 1.693521 \| \| C \| 3.214527 \| -2.209531 \| 0.863525 \| \| C \| 0.663708 \| -1.118460 \| 0.024901 \| \| C \| -3.407128 \| -3.826069 \| 0.947433 \| \| H \| -2.749129 \| -4.670684 \| 1.135995 \| \| C \| 3.740927 \| -0.919457 \| 0.593206 \| \| C \| -3.776679 \| -1.631882 \| 0.001667 \| \| C \| -5.075100 \| -1.645439 \| 0.508937 \| \| H \| -5.731447 \| -0.793114 \| 0.365747 \| \| C \| -4.700433 \| -3.836183 \| 1.459755 \| \| B \| -1.394879 \| -2.673587 \| -0.145991 \| \| C \| 5.336183 \| -2.954060 \| 1.778891 \| \| H \| 5.945350 \| -3.739201 \| 2.216597 \| \| C \| 5.847770 \| -1.668325 \| 1.615998 \| \| C \| 5.055175 \| -0.680023 \| 1.049673 \| \| H \| 5.460442 \| 0.322912 \| 0.980661 \| \| C \| -5.528606 \| -2.738788 \| 1.244574 \| \| H \| -6.533729 \| -2.725896 \| 1.656062 \| \| C \| -1.566514 \| 4.418636 \| 1.394467 \| \| H \| -2.606807 \| 4.236388 \| 1.649587 \| \| H \| 2.177184 \| 5.007759 \| 0.633146 \| \| H \| 1.027956 \| 6.401719 \| 2.317868 \| \| H \| -0.619609 \| -7.012688 \| -1.865784 \| \| H \| 6.854598 \| -1.433434 \| 1.947005 \| \| H \| 6.753429 \| 2.431305 \| -1.825182 \| \| H \| -1.367345 \| 6.000211 \| 2.840415 \| \| H \| -5.848197 \| 4.660773 \| -0.820061 \| \| H \| -5.057195 \| -4.688080 \| 2.029967 \| |

**Table S2:** Geometries, energies (in Hartree) and Cartesian coordinates (in Å) of the complexes in vacuum. Level of theory : M06-2X/6-31+G*

| 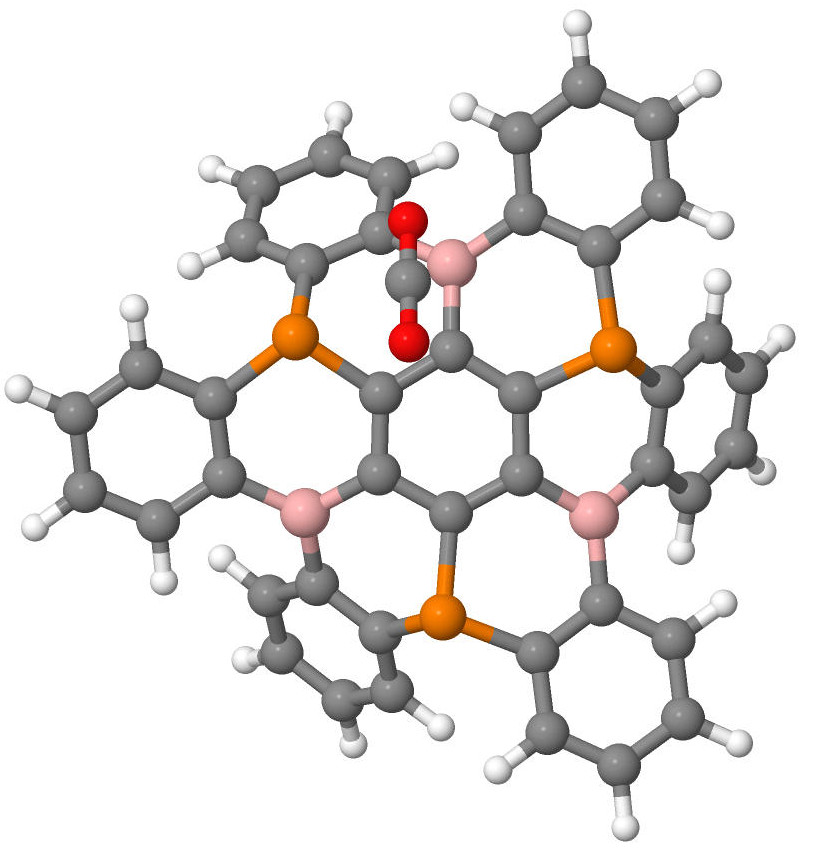 | B3P3-NG:CO2 E=--2901.420399H FreqIm=0   \| C \| -3.704652 \| 3.910573 \| -2.355269 \| \| --- \| --- \| --- \| --- \| \| C \| -3.411223 \| 2.051614 \| -0.290759 \| \| C \| -2.344971 \| 2.942505 \| -0.565470 \| \| C \| -4.606735 \| 2.122362 \| -1.005927 \| \| C \| -4.751580 \| 3.049299 \| -2.037749 \| \| P \| 2.171006 \| 2.207327 \| 0.733476 \| \| P \| -3.165703 \| 0.888628 \| 1.111666 \| \| C \| -0.524081 \| 1.375747 \| 0.594372 \| \| C \| 0.033333 \| 4.016429 \| 0.265242 \| \| C \| 0.823441 \| 1.008071 \| 0.537278 \| \| C \| -5.601757 \| -0.385597 \| 1.134474 \| \| H \| -5.977370 \| 0.598368 \| 1.409469 \| \| C \| -4.240492 \| -0.555346 \| 0.843341 \| \| C \| -1.488862 \| 0.327376 \| 0.701673 \| \| C \| -0.453646 \| 5.339316 \| 0.261433 \| \| H \| -1.525127 \| 5.503529 \| 0.184500 \| \| C \| -3.727244 \| -1.840245 \| 0.546445 \| \| C \| 1.426874 \| 3.839496 \| 0.435274 \| \| C \| 2.272484 \| 4.949714 \| 0.571364 \| \| H \| 3.336001 \| 4.801479 \| 0.749810 \| \| C \| 0.388961 \| 6.438427 \| 0.377794 \| \| B \| -0.953485 \| 2.809424 \| 0.159337 \| \| C \| -6.475165 \| -1.467295 \| 1.110834 \| \| H \| -7.529087 \| -1.317559 \| 1.327648 \| \| C \| -5.983937 \| -2.746535 \| 0.848749 \| \| C \| -4.631918 \| -2.920977 \| 0.578292 \| \| H \| -4.255424 \| -3.925556 \| 0.404699 \| \| C \| 1.763359 \| 6.242435 \| 0.515406 \| \| H \| 2.432543 \| 7.092805 \| 0.611130 \| \| B \| -2.210911 \| -2.086419 \| 0.262249 \| \| C \| -1.135786 \| -1.024327 \| 0.645035 \| \| C \| 5.141087 \| -3.700540 \| 0.084374 \| \| C \| 2.387918 \| -3.288499 \| 0.377695 \| \| C \| 3.215776 \| -2.186054 \| 0.063783 \| \| C \| 4.599239 \| -2.428445 \| -0.051867 \| \| H \| 5.265012 \| -1.587810 \| -0.228553 \| \| C \| 2.940506 \| -4.568465 \| 0.535642 \| \| H \| 2.302404 \| -5.401217 \| 0.826085 \| \| C \| 4.302968 \| -4.781519 \| 0.361074 \| \| H \| 4.714293 \| -5.780547 \| 0.474891 \| \| P \| 0.641121 \| -3.094976 \| 0.850629 \| \| C \| 1.245346 \| -0.356744 \| 0.480368 \| \| C \| 3.392010 \| 0.359359 \| -0.937051 \| \| C \| 0.054039 \| -4.832627 \| -1.319362 \| \| H \| 1.073026 \| -5.200232 \| -1.236321 \| \| C \| -0.396639 \| -3.806216 \| -0.489281 \| \| C \| 0.256506 \| -1.340598 \| 0.584582 \| \| C \| 4.166630 \| -0.011614 \| -2.049878 \| \| H \| 4.308120 \| -1.066862 \| -2.267643 \| \| C \| -1.720589 \| -3.314300 \| -0.592743 \| \| C \| 3.173762 \| 1.743628 \| -0.733161 \| \| C \| 3.756968 \| 2.694431 \| -1.570695 \| \| H \| 3.579432 \| 3.753963 \| -1.408460 \| \| C \| 4.731609 \| 0.933209 \| -2.903425 \| \| B \| 2.658892 \| -0.731236 \| -0.067884 \| \| C \| -0.786624 \| -5.361136 \| -2.298403 \| \| H \| -0.418976 \| -6.143698 \| -2.956504 \| \| C \| -2.080551 \| -4.869059 \| -2.448120 \| \| C \| -2.533944 \| -3.860141 \| -1.601543 \| \| H \| -3.536772 \| -3.466075 \| -1.743934 \| \| C \| 4.533257 \| 2.288786 \| -2.655674 \| \| H \| 4.964053 \| 3.035393 \| -3.317187 \| \| C \| -2.521357 \| 3.852443 \| -1.622721 \| \| H \| -1.701947 \| 4.512217 \| -1.895624 \| \| H \| -5.418050 \| 1.432309 \| -0.791282 \| \| H \| -5.677668 \| 3.080905 \| -2.605202 \| \| O \| 2.018085 \| 0.278314 \| 3.836578 \| \| C \| 2.935856 \| 0.056100 \| 3.160306 \| \| O \| 3.863844 \| -0.181351 \| 2.496211 \| \| H \| -0.022438 \| 7.443588 \| 0.374399 \| \| H \| -3.806286 \| 4.619442 \| -3.171711 \| \| H \| -6.651117 \| -3.603335 \| 0.868432 \| \| H \| -2.730903 \| -5.263774 \| -3.223146 \| \| H \| 6.213009 \| -3.849349 \| -0.007808 \| \| H \| 5.317960 \| 0.612519 \| -3.759464 \| |
| --- | --- | --- | --- | --- | --- | --- | --- | --- | --- | --- | --- | --- | --- | --- | --- | --- | --- | --- | --- | --- | --- | --- | --- | --- | --- | --- | --- | --- | --- | --- | --- | --- | --- | --- | --- | --- | --- | --- | --- | --- | --- | --- | --- | --- | --- | --- | --- | --- | --- | --- | --- | --- | --- | --- | --- | --- | --- | --- | --- | --- | --- | --- | --- | --- | --- | --- | --- | --- | --- | --- | --- | --- | --- | --- | --- | --- | --- | --- | --- | --- | --- | --- | --- | --- | --- | --- | --- | --- | --- | --- | --- | --- | --- | --- | --- | --- | --- | --- | --- | --- | --- | --- | --- | --- | --- | --- | --- | --- | --- | --- | --- | --- | --- | --- | --- | --- | --- | --- | --- | --- | --- | --- | --- | --- | --- | --- | --- | --- | --- | --- | --- | --- | --- | --- | --- | --- | --- | --- | --- | --- | --- | --- | --- | --- | --- | --- | --- | --- | --- | --- | --- | --- | --- | --- | --- | --- | --- | --- | --- | --- | --- | --- | --- | --- | --- | --- | --- | --- | --- | --- | --- | --- | --- | --- | --- | --- | --- | --- | --- | --- | --- | --- | --- | --- | --- | --- | --- | --- | --- | --- | --- | --- | --- | --- | --- | --- | --- | --- | --- | --- | --- | --- | --- | --- | --- | --- | --- | --- | --- | --- | --- | --- | --- | --- | --- | --- | --- | --- | --- | --- | --- | --- | --- | --- | --- | --- | --- | --- | --- | --- | --- | --- | --- | --- | --- | --- | --- | --- | --- | --- | --- | --- | --- | --- | --- | --- | --- | --- | --- | --- | --- | --- | --- | --- | --- | --- | --- | --- | --- | --- | --- | --- | --- | --- | --- | --- | --- | --- | --- | --- | --- | --- | --- | --- | --- | --- | --- | --- | --- | --- | --- | --- | --- | --- | --- | --- | --- | --- | --- | --- | --- | --- | --- | --- | --- | --- | --- | --- | --- | --- | --- |
| 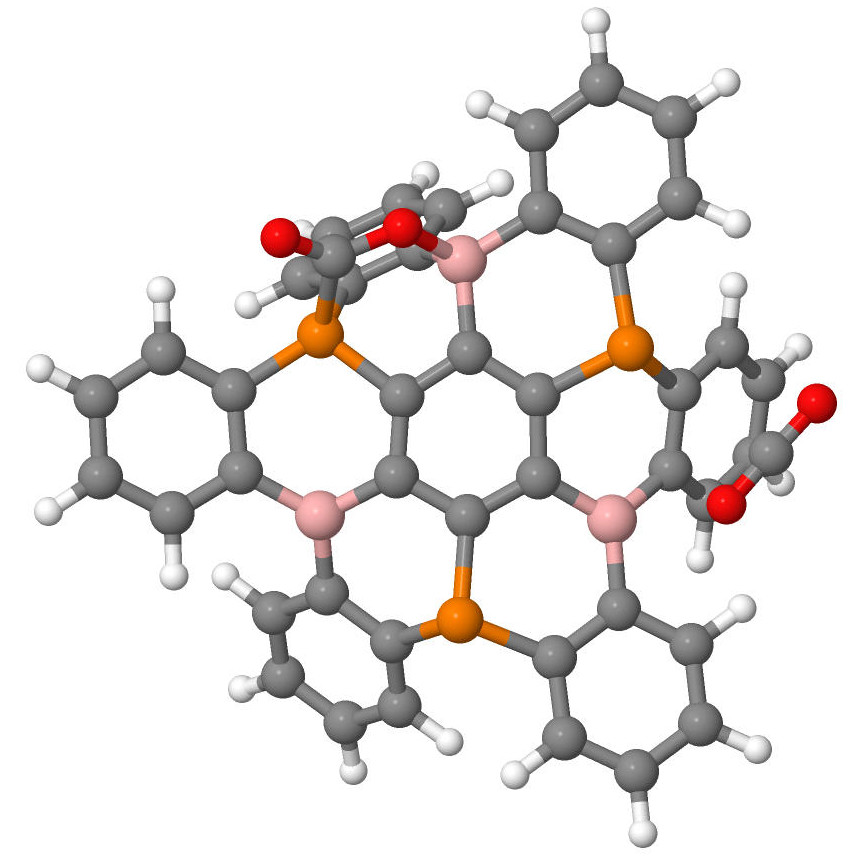 | 1CO2-B3P3-NG:CO2 E=-3089.942996H FreqIm=0   \| C \| -3.133258 \| 4.883517 \| 2.004378 \| \| --- \| --- \| --- \| --- \| \| C \| -1.163562 \| 3.933814 \| 0.248268 \| \| C \| -2.392716 \| 3.228989 \| 0.359505 \| \| C \| -0.952884 \| 5.101637 \| 0.983112 \| \| C \| -1.928659 \| 5.568801 \| 1.862361 \| \| P \| -2.916998 \| -1.278648 \| -0.406191 \| \| P \| 0.044736 \| 3.344702 \| -1.008350 \| \| C \| -1.329912 \| 1.007858 \| -0.648695 \| \| C \| -4.039822 \| 1.225035 \| -0.573891 \| \| C \| -1.383755 \| -0.386260 \| -0.604111 \| \| C \| 2.044139 \| 5.209015 \| -0.695277 \| \| H \| 1.273362 \| 5.912660 \| -1.003892 \| \| C \| 1.724187 \| 3.853962 \| -0.522246 \| \| C \| -0.026759 \| 1.566539 \| -0.679490 \| \| C \| -5.135385 \| 2.060858 \| -0.842964 \| \| H \| -4.985411 \| 3.137091 \| -0.856224 \| \| C \| 2.734940 \| 2.915328 \| -0.201398 \| \| C \| -4.298801 \| -0.168556 \| -0.612132 \| \| C \| -5.564432 \| -0.695071 \| -0.878286 \| \| H \| -5.712378 \| -1.771017 \| -0.922351 \| \| C \| -6.403309 \| 1.549139 \| -1.119133 \| \| B \| -2.601503 \| 1.844895 \| -0.335723 \| \| C \| 3.346380 \| 5.662007 \| -0.519031 \| \| H \| 3.574887 \| 6.716566 \| -0.644687 \| \| C \| 4.360652 \| 4.750070 \| -0.226492 \| \| C \| 4.051472 \| 3.403274 \| -0.082152 \| \| H \| 4.854861 \| 2.696274 \| 0.106649 \| \| C \| -6.624525 \| 0.173864 \| -1.126646 \| \| H \| -7.610894 \| -0.223661 \| -1.343994 \| \| B \| 2.450944 \| 1.383291 \| -0.055373 \| \| C \| 1.120480 \| 0.758274 \| -0.587547 \| \| C \| 1.484805 \| -6.114166 \| -0.331481 \| \| C \| 1.998109 \| -3.369516 \| -0.485789 \| \| C \| 0.661094 \| -3.806642 \| -0.396270 \| \| C \| 0.442895 \| -5.192985 \| -0.324481 \| \| H \| -0.583605 \| -5.551862 \| -0.266895 \| \| C \| 3.050016 \| -4.300696 \| -0.537004 \| \| H \| 4.072349 \| -3.954633 \| -0.685004 \| \| C \| 2.801458 \| -5.664018 \| -0.436071 \| \| H \| 3.626021 \| -6.370647 \| -0.465665 \| \| P \| 2.480599 \| -1.635345 \| -0.813634 \| \| C \| -0.292277 \| -1.262115 \| -0.608581 \| \| C \| -1.480669 \| -2.929176 \| 1.041347 \| \| C \| 4.097983 \| -1.782606 \| 1.520218 \| \| H \| 4.103407 \| -2.859728 \| 1.380308 \| \| C \| 3.363004 \| -0.965559 \| 0.659274 \| \| C \| 0.963929 \| -0.655603 \| -0.630563 \| \| C \| -1.139074 \| -3.657302 \| 2.184552 \| \| H \| -0.215828 \| -4.231952 \| 2.191006 \| \| C \| 3.347399 \| 0.443022 \| 0.836438 \| \| C \| -2.684714 \| -2.200721 \| 1.117961 \| \| C \| -3.529727 \| -2.188481 \| 2.224096 \| \| H \| -4.448079 \| -1.605845 \| 2.222782 \| \| C \| -1.963028 \| -3.661549 \| 3.313600 \| \| B \| -0.608540 \| -2.835789 \| -0.358217 \| \| C \| 4.791199 \| -1.229301 \| 2.595464 \| \| H \| 5.339486 \| -1.879139 \| 3.272057 \| \| C \| 4.760191 \| 0.145710 \| 2.814268 \| \| C \| 4.047065 \| 0.962658 \| 1.940845 \| \| H \| 4.008673 \| 2.031131 \| 2.136044 \| \| C \| -3.155387 \| -2.937338 \| 3.338529 \| \| H \| -3.786850 \| -2.950194 \| 4.221640 \| \| C \| -3.353815 \| 3.730924 \| 1.257034 \| \| H \| -4.284882 \| 3.186764 \| 1.394237 \| \| H \| -0.009898 \| 5.635068 \| 0.906044 \| \| H \| -1.736777 \| 6.462872 \| 2.449221 \| \| O \| -3.618451 \| -2.914285 \| -2.468856 \| \| C \| -2.742110 \| -2.683577 \| -1.674154 \| \| O \| -1.616204 \| -3.287146 \| -1.489618 \| \| O \| 4.421058 \| 0.817573 \| -2.150684 \| \| C \| 5.085128 \| -0.125987 \| -1.989083 \| \| O \| 5.773793 \| -1.049023 \| -1.833358 \| \| H \| -7.222065 \| 2.229656 \| -1.334111 \| \| H \| 5.388496 \| 5.087275 \| -0.129789 \| \| H \| -3.889380 \| 5.239675 \| 2.697473 \| \| H \| 5.282965 \| 0.578732 \| 3.662043 \| \| H \| -1.671052 \| -4.238528 \| 4.186898 \| \| H \| 1.273804 \| -7.178533 \| -0.274173 \| |
| 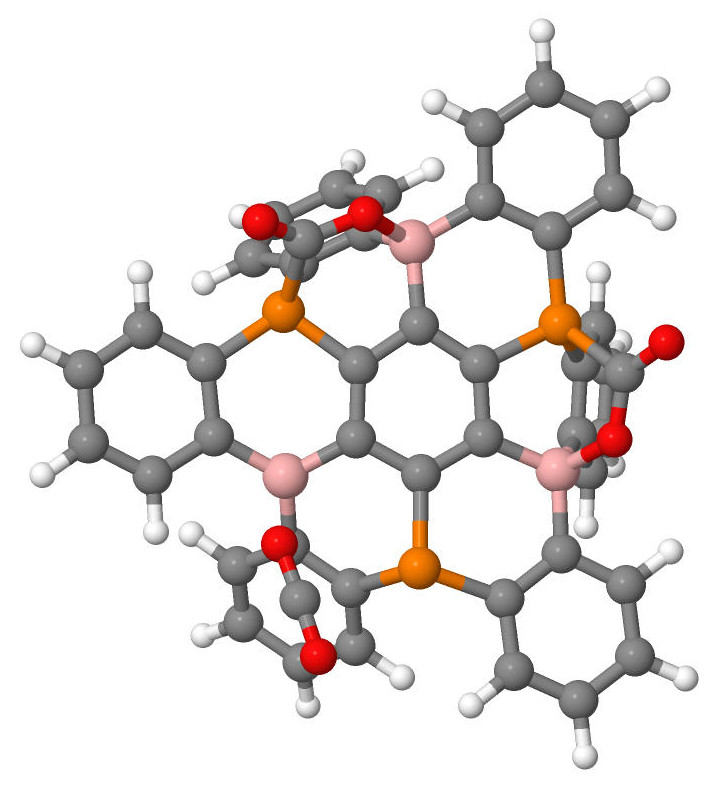 | 2CO2-B3P3-NG:CO2 E=-3278.473063H FreqIm=0   \| C \| -5.109456 \| -0.573139 \| 2.731955 \| \| --- \| --- \| --- \| --- \| \| C \| -3.659121 \| 0.728835 \| 0.707814 \| \| C \| -3.523433 \| -0.680817 \| 0.869147 \| \| C \| -4.526707 \| 1.445306 \| 1.535701 \| \| C \| -5.238804 \| 0.801602 \| 2.546189 \| \| P \| 0.262514 \| -3.087492 \| -0.201024 \| \| P \| -2.804449 \| 1.527822 \| -0.719159 \| \| C \| -1.210258 \| -0.707317 \| -0.427402 \| \| C \| -2.485866 \| -3.081731 \| -0.057774 \| \| C \| 0.028597 \| -1.349948 \| -0.476086 \| \| C \| -3.604180 \| 4.133683 \| -0.384669 \| \| H \| -4.597086 \| 3.702112 \| -0.500527 \| \| C \| -2.473479 \| 3.298244 \| -0.378689 \| \| C \| -1.203463 \| 0.702932 \| -0.551458 \| \| C \| -3.715172 \| -3.753129 \| -0.142570 \| \| H \| -4.632005 \| -3.171000 \| -0.101424 \| \| C \| -1.175226 \| 3.854629 \| -0.347458 \| \| C \| -1.328279 \| -3.897715 \| -0.163553 \| \| C \| -1.392821 \| -5.284604 \| -0.318831 \| \| H \| -0.480854 \| -5.867889 \| -0.418806 \| \| C \| -3.794622 \| -5.135709 \| -0.306033 \| \| B \| -2.458702 \| -1.498162 \| 0.067203 \| \| C \| -3.475192 \| 5.513838 \| -0.288087 \| \| H \| -4.360551 \| 6.143245 \| -0.282627 \| \| C \| -2.201442 \| 6.079975 \| -0.237098 \| \| C \| -1.082547 \| 5.255135 \| -0.279889 \| \| H \| -0.090777 \| 5.704416 \| -0.266856 \| \| C \| -2.636781 \| -5.906566 \| -0.383414 \| \| H \| -2.698860 \| -6.982366 \| -0.513349 \| \| B \| 0.188462 \| 3.012587 \| -0.373105 \| \| C \| -0.002206 \| 1.417019 \| -0.588896 \| \| C \| 6.381214 \| -0.856727 \| -1.204325 \| \| C \| 4.100308 \| 0.638932 \| -0.758535 \| \| C \| 3.974220 \| -0.772467 \| -0.758988 \| \| C \| 5.158466 \| -1.488092 \| -0.989874 \| \| H \| 5.108088 \| -2.574639 \| -1.010081 \| \| C \| 5.323224 \| 1.289198 \| -0.981154 \| \| H \| 5.368199 \| 2.375694 \| -1.006955 \| \| C \| 6.469582 \| 0.536092 \| -1.199337 \| \| H \| 7.419318 \| 1.030344 \| -1.377763 \| \| P \| 2.649078 \| 1.662696 \| -0.535280 \| \| C \| 1.264527 \| -0.714441 \| -0.599566 \| \| C \| 2.535638 \| -2.373614 \| 0.957048 \| \| C \| 3.285067 \| 2.653793 \| 2.057291 \| \| H \| 4.252566 \| 2.158860 \| 2.010456 \| \| C \| 2.392000 \| 2.580184 \| 0.992666 \| \| C \| 1.179351 \| 0.665259 \| -0.631966 \| \| C \| 3.481359 \| -2.323342 \| 1.982612 \| \| H \| 4.378621 \| -1.722269 \| 1.849639 \| \| C \| 1.119823 \| 3.190574 \| 0.979792 \| \| C \| 1.395218 \| -3.169684 \| 1.201841 \| \| C \| 1.191143 \| -3.895065 \| 2.371243 \| \| H \| 0.294299 \| -4.494588 \| 2.509327 \| \| C \| 3.298218 \| -3.036148 \| 3.171819 \| \| B \| 2.611304 \| -1.598847 \| -0.492001 \| \| C \| 2.898273 \| 3.369316 \| 3.189981 \| \| H \| 3.569431 \| 3.445542 \| 4.040091 \| \| C \| 1.643882 \| 3.977771 \| 3.225295 \| \| C \| 0.768820 \| 3.888385 \| 2.138576 \| \| H \| -0.203591 \| 4.372576 \| 2.192335 \| \| C \| 2.163312 \| -3.821251 \| 3.369313 \| \| H \| 2.031256 \| -4.370377 \| 4.296461 \| \| C \| -4.261503 \| -1.295223 \| 1.898499 \| \| H \| -4.144321 \| -2.362827 \| 2.067942 \| \| H \| -4.622228 \| 2.521242 \| 1.423535 \| \| H \| -5.885318 \| 1.382267 \| 3.198543 \| \| O \| 1.271197 \| -4.470360 \| -2.329930 \| \| C \| 1.477761 \| -3.541733 \| -1.592787 \| \| O \| 2.493483 \| -2.734108 \| -1.563887 \| \| O \| 1.092850 \| 3.527739 \| -1.553405 \| \| C \| 2.269320 \| 3.038799 \| -1.792900 \| \| O \| 3.064939 \| 3.358263 \| -2.638680 \| \| O \| -3.857358 \| -1.205744 \| -2.278438 \| \| C \| -4.616270 \| -0.387236 \| -2.619173 \| \| O \| -5.398938 \| 0.391146 \| -2.976537 \| \| H \| -2.083774 \| 7.158855 \| -0.184854 \| \| H \| -5.656730 \| -1.075031 \| 3.524213 \| \| H \| -4.767494 \| -5.613093 \| -0.378669 \| \| H \| 4.051271 \| -2.979024 \| 3.952983 \| \| H \| 7.271327 \| -1.453257 \| -1.384356 \| \| H \| 1.342376 \| 4.529612 \| 4.111593 \| |
| 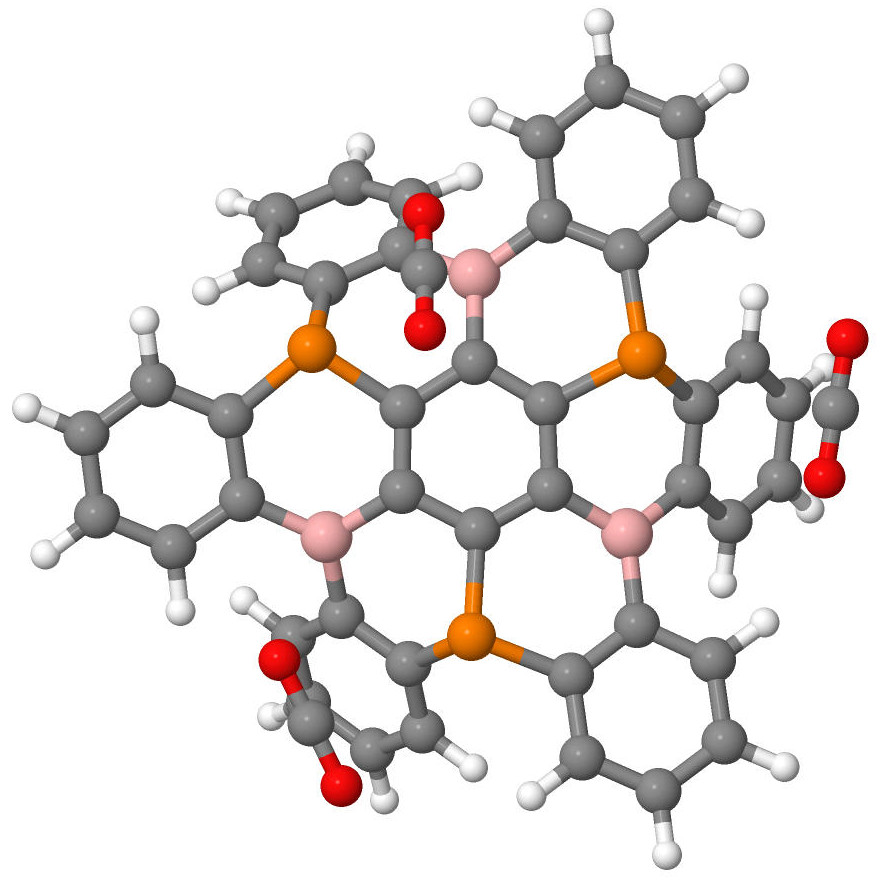 | B3P3-NG:3CO2 E=-3278.468742H FreqIm=0   \| C \| -4.872111 \| 1.850789 \| 2.772876 \| \| --- \| --- \| --- \| --- \| \| C \| -3.024574 \| 2.278891 \| 0.724437 \| \| C \| -3.540442 \| 0.973629 \| 0.916705 \| \| C \| -3.461319 \| 3.347387 \| 1.508255 \| \| C \| -4.382205 \| 3.132127 \| 2.532696 \| \| P \| -1.471731 \| -2.996987 \| -0.688792 \| \| P \| -1.858986 \| 2.499797 \| -0.679803 \| \| C \| -1.509077 \| -0.192631 \| -0.362480 \| \| C \| -3.845735 \| -1.557698 \| -0.091017 \| \| C \| -0.742973 \| -1.361783 \| -0.395052 \| \| C \| -1.399240 \| 5.205099 \| -0.554072 \| \| H \| -2.456010 \| 5.274980 \| -0.807269 \| \| C \| -0.814240 \| 3.948307 \| -0.344588 \| \| C \| -0.808522 \| 1.051314 \| -0.392228 \| \| C \| -5.253784 \| -1.506674 \| -0.062098 \| \| H \| -5.740029 \| -0.544792 \| 0.076091 \| \| C \| 0.570748 \| 3.835326 \| -0.078083 \| \| C \| -3.248624 \| -2.813927 \| -0.348410 \| \| C \| -4.043915 \| -3.952653 \| -0.543175 \| \| H \| -3.576058 \| -4.904026 \| -0.789860 \| \| C \| -6.039112 \| -2.639839 \| -0.234502 \| \| B \| -3.001884 \| -0.254892 \| 0.086648 \| \| C \| -0.633508 \| 6.363662 \| -0.478087 \| \| H \| -1.100820 \| 7.332224 \| -0.631872 \| \| C \| 0.739508 \| 6.272225 \| -0.247346 \| \| C \| 1.325149 \| 5.025786 \| -0.057808 \| \| H \| 2.400640 \| 4.963151 \| 0.085958 \| \| C \| -5.429250 \| -3.874558 \| -0.459118 \| \| H \| -6.031965 \| -4.767168 \| -0.600914 \| \| B \| 1.268991 \| 2.452598 \| 0.116154 \| \| C \| 0.586171 \| 1.130450 \| -0.348579 \| \| C \| 5.078541 \| -4.030517 \| -0.218525 \| \| C \| 3.832534 \| -1.528650 \| -0.312831 \| \| C \| 3.043107 \| -2.676273 \| -0.069565 \| \| C \| 3.703169 \| -3.921122 \| -0.052967 \| \| H \| 3.112796 \| -4.825486 \| 0.068344 \| \| C \| 5.218267 \| -1.642020 \| -0.500942 \| \| H \| 5.806713 \| -0.757352 \| -0.737083 \| \| C \| 5.843213 \| -2.881672 \| -0.424778 \| \| H \| 6.918564 \| -2.954673 \| -0.560754 \| \| P \| 3.099439 \| 0.100379 \| -0.652539 \| \| C \| 0.686405 \| -1.344650 \| -0.357265 \| \| C \| 0.692225 \| -3.698912 \| 0.901166 \| \| C \| 4.615572 \| 1.064373 \| 1.547687 \| \| H \| 5.288350 \| 0.226436 \| 1.386539 \| \| C \| 3.470539 \| 1.211959 \| 0.764125 \| \| C \| 1.315681 \| -0.096363 \| -0.382820 \| \| C \| 1.303130 \| -4.396407 \| 1.957797 \| \| H \| 2.354568 \| -4.221100 \| 2.169219 \| \| C \| 2.593645 \| 2.307545 \| 0.955211 \| \| C \| -0.695788 \| -3.903211 \| 0.707274 \| \| C \| -1.406208 \| -4.806309 \| 1.498084 \| \| H \| -2.471829 \| -4.952379 \| 1.344839 \| \| C \| 0.592959 \| -5.281628 \| 2.765434 \| \| B \| 1.489265 \| -2.607905 \| 0.091785 \| \| C \| 4.886575 \| 1.975507 \| 2.568652 \| \| H \| 5.769371 \| 1.841205 \| 3.187652 \| \| C \| 4.016445 \| 3.036403 \| 2.806795 \| \| C \| 2.887987 \| 3.194146 \| 2.004766 \| \| H \| 2.207020 \| 4.016010 \| 2.209819 \| \| C \| -0.762346 \| -5.492782 \| 2.526971 \| \| H \| -1.328833 \| -6.176387 \| 3.153375 \| \| C \| -4.454070 \| 0.792078 \| 1.969644 \| \| H \| -4.827820 \| -0.206735 \| 2.179184 \| \| H \| -3.062794 \| 4.345434 \| 1.348065 \| \| H \| -4.700341 \| 3.965359 \| 3.153140 \| \| O \| 0.262948 \| -2.025431 \| -3.759126 \| \| C \| 0.769247 \| -2.876119 \| -3.151675 \| \| O \| 1.293252 \| -3.730369 \| -2.556485 \| \| H \| -7.122094 \| -2.561613 \| -0.209566 \| \| H \| -5.573834 \| 1.675652 \| 3.583061 \| \| H \| 1.351342 \| 7.169428 \| -0.230302 \| \| H \| 4.214127 \| 3.736491 \| 3.613191 \| \| H \| 5.552952 \| -5.007475 \| -0.203470 \| \| H \| 1.093529 \| -5.798478 \| 3.578899 \| \| O \| 4.505226 \| 3.486030 \| -1.408267 \| \| C \| 5.283420 \| 2.624240 \| -1.359128 \| \| O \| 6.077222 \| 1.773279 \| -1.319883 \| \| O \| -4.987972 \| 3.432666 \| -1.831704 \| \| C \| -4.837974 \| 2.284667 \| -1.942370 \| \| O \| -4.711371 \| 1.133494 \| -2.059109 \| |

**Table S3:** Geometries, energies (in Hartree) and Cartesian coordinates (in Å) of the transition states in vacuum. Level of theory : M06-2X/6-31+G*

| 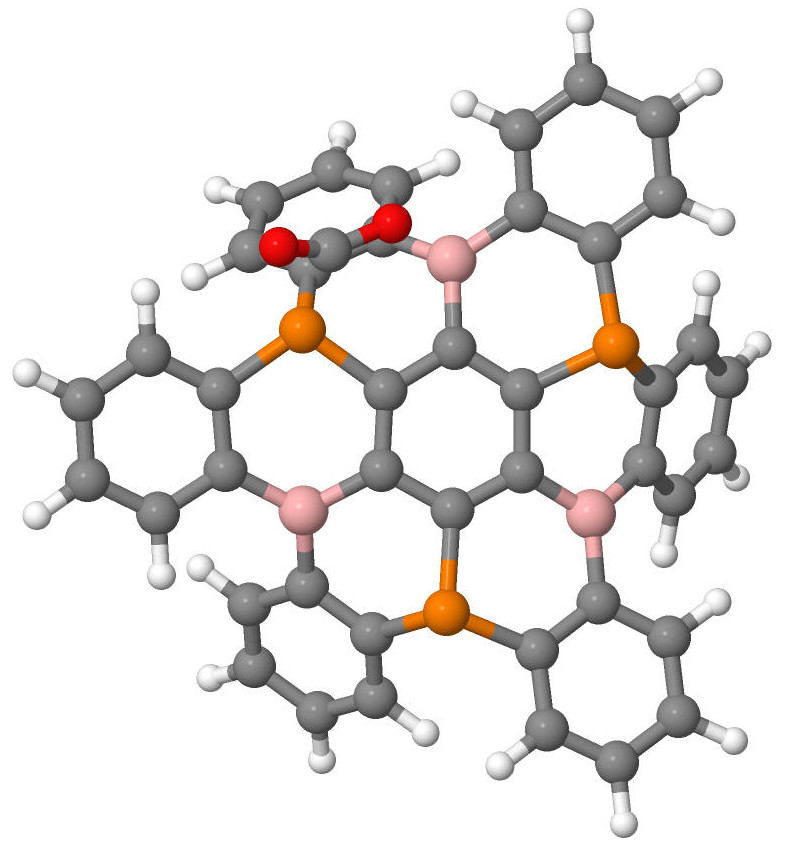 | B3P3-NG/CO2 E=-2901.400074H FreqIm=1   \| C \| 2.758723 \| -4.735631 \| -2.231635 \| \| --- \| --- \| --- \| --- \| \| C \| 2.908658 \| -2.762857 \| -0.252097 \| \| C \| 1.665554 \| -3.402221 \| -0.494571 \| \| C \| 4.049659 \| -3.134395 \| -0.963633 \| \| C \| 3.973152 \| -4.113474 \| -1.953671 \| \| P \| -2.523093 \| -1.564492 \| 0.469053 \| \| P \| 2.960854 \| -1.542548 \| 1.122132 \| \| C \| 0.273710 \| -1.415435 \| 0.610924 \| \| C \| -0.921952 \| -3.843417 \| 0.308666 \| \| C \| -0.938188 \| -0.725243 \| 0.529052 \| \| C \| 5.621618 \| -0.852336 \| 1.069744 \| \| H \| 5.774344 \| -1.900222 \| 1.321195 \| \| C \| 4.325644 \| -0.375150 \| 0.825076 \| \| C \| 1.447698 \| -0.620471 \| 0.742433 \| \| C \| -0.780805 \| -5.238484 \| 0.411595 \| \| H \| 0.217249 \| -5.668425 \| 0.400714 \| \| C \| 4.106287 \| 1.000793 \| 0.569756 \| \| C \| -2.241661 \| -3.334703 \| 0.378545 \| \| C \| -3.349664 \| -4.175174 \| 0.525519 \| \| H \| -4.347530 \| -3.751661 \| 0.616698 \| \| C \| -1.879859 \| -6.082932 \| 0.551673 \| \| B \| 0.344169 \| -2.916780 \| 0.197410 \| \| C \| 6.715354 \| 0.005393 \| 1.034637 \| \| H \| 7.713880 \| -0.382765 \| 1.214666 \| \| C \| 6.517974 \| 1.368199 \| 0.811575 \| \| C \| 5.232600 \| 1.848591 \| 0.591776 \| \| H \| 5.087043 \| 2.916416 \| 0.452758 \| \| C \| -3.168543 \| -5.553984 \| 0.594154 \| \| H \| -4.027717 \| -6.207931 \| 0.708254 \| \| B \| 2.677919 \| 1.594060 \| 0.346025 \| \| C \| 1.401320 \| 0.780408 \| 0.727987 \| \| C \| -4.157057 \| 4.800947 \| 0.485568 \| \| C \| -1.556683 \| 3.772079 \| 0.668620 \| \| C \| -2.625692 \| 2.897038 \| 0.373937 \| \| C \| -3.919443 \| 3.443866 \| 0.302686 \| \| H \| -4.757263 \| 2.775143 \| 0.117740 \| \| C \| -1.803079 \| 5.138776 \| 0.872295 \| \| H \| -0.985441 \| 5.800490 \| 1.153025 \| \| C \| -3.088324 \| 5.656112 \| 0.757233 \| \| H \| -3.260061 \| 6.717953 \| 0.909605 \| \| P \| 0.128952 \| 3.192587 \| 1.068882 \| \| C \| -1.057425 \| 0.682533 \| 0.557013 \| \| C \| -3.237582 \| 0.590292 \| -0.982851 \| \| C \| 1.032117 \| 4.829673 \| -1.073895 \| \| H \| 0.123109 \| 5.410803 \| -0.945112 \| \| C \| 1.263361 \| 3.700515 \| -0.288405 \| \| C \| 0.122688 \| 1.410587 \| 0.712357 \| \| C \| -3.838314 \| 1.242305 \| -2.067510 \| \| H \| -3.790004 \| 2.326467 \| -2.128297 \| \| C \| 2.443437 \| 2.931022 \| -0.451791 \| \| C \| -3.298362 \| -0.821042 \| -0.982658 \| \| C \| -3.952984 \| -1.546526 \| -1.974715 \| \| H \| -3.982434 \| -2.633037 \| -1.937790 \| \| C \| -4.483944 \| 0.530561 \| -3.080057 \| \| B \| -2.413348 \| 1.364933 \| 0.140725 \| \| C \| 1.941958 \| 5.192373 \| -2.066760 \| \| H \| 1.739575 \| 6.059417 \| -2.689662 \| \| C \| 3.089072 \| 4.431364 \| -2.275301 \| \| C \| 3.329384 \| 3.318519 \| -1.472812 \| \| H \| 4.214591 \| 2.717081 \| -1.662323 \| \| C \| -4.547526 \| -0.860276 \| -3.033309 \| \| H \| -5.048876 \| -1.412911 \| -3.822455 \| \| C \| 1.625468 \| -4.379813 \| -1.505322 \| \| H \| 0.676965 \| -4.851970 \| -1.747818 \| \| H \| 4.997976 \| -2.637488 \| -0.779935 \| \| H \| 4.863333 \| -4.374800 \| -2.519228 \| \| O \| -4.206830 \| -1.523722 \| 2.747692 \| \| C \| -3.703536 \| -0.695687 \| 2.045937 \| \| O \| -3.609286 \| 0.501507 \| 1.834918 \| \| H \| -1.729928 \| -7.155256 \| 0.636700 \| \| H \| 2.693167 \| -5.486969 \| -3.012865 \| \| H \| 7.361169 \| 2.052550 \| 0.824607 \| \| H \| 3.789293 \| 4.698518 \| -3.061234 \| \| H \| -5.169344 \| 5.190847 \| 0.431320 \| \| H \| -4.937450 \| 1.065025 \| -3.909834 \| |
| --- | --- | --- | --- | --- | --- | --- | --- | --- | --- | --- | --- | --- | --- | --- | --- | --- | --- | --- | --- | --- | --- | --- | --- | --- | --- | --- | --- | --- | --- | --- | --- | --- | --- | --- | --- | --- | --- | --- | --- | --- | --- | --- | --- | --- | --- | --- | --- | --- | --- | --- | --- | --- | --- | --- | --- | --- | --- | --- | --- | --- | --- | --- | --- | --- | --- | --- | --- | --- | --- | --- | --- | --- | --- | --- | --- | --- | --- | --- | --- | --- | --- | --- | --- | --- | --- | --- | --- | --- | --- | --- | --- | --- | --- | --- | --- | --- | --- | --- | --- | --- | --- | --- | --- | --- | --- | --- | --- | --- | --- | --- | --- | --- | --- | --- | --- | --- | --- | --- | --- | --- | --- | --- | --- | --- | --- | --- | --- | --- | --- | --- | --- | --- | --- | --- | --- | --- | --- | --- | --- | --- | --- | --- | --- | --- | --- | --- | --- | --- | --- | --- | --- | --- | --- | --- | --- | --- | --- | --- | --- | --- | --- | --- | --- | --- | --- | --- | --- | --- | --- | --- | --- | --- | --- | --- | --- | --- | --- | --- | --- | --- | --- | --- | --- | --- | --- | --- | --- | --- | --- | --- | --- | --- | --- | --- | --- | --- | --- | --- | --- | --- | --- | --- | --- | --- | --- | --- | --- | --- | --- | --- | --- | --- | --- | --- | --- | --- | --- | --- | --- | --- | --- | --- | --- | --- | --- | --- | --- | --- | --- | --- | --- | --- | --- | --- | --- | --- | --- | --- | --- | --- | --- | --- | --- | --- | --- | --- | --- | --- | --- | --- | --- | --- | --- | --- | --- | --- | --- | --- | --- | --- | --- | --- | --- | --- | --- | --- | --- | --- | --- | --- | --- | --- | --- | --- | --- | --- | --- | --- | --- | --- | --- | --- | --- | --- | --- | --- | --- | --- | --- | --- | --- | --- | --- | --- | --- | --- | --- | --- | --- | --- | --- |
| 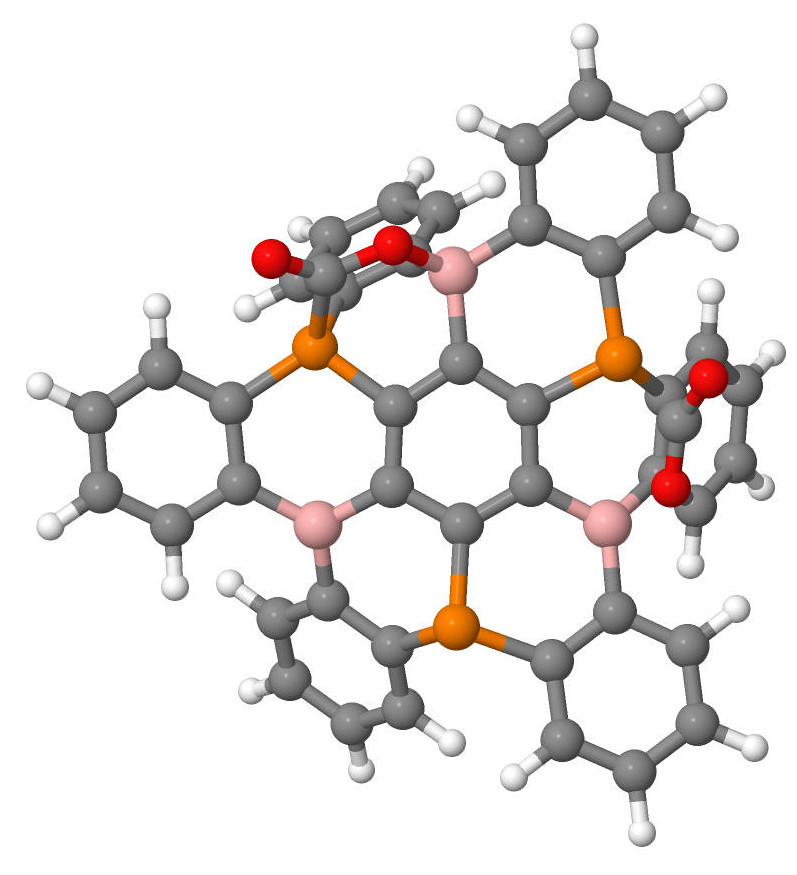 | 1CO2-B3P3-NG/CO2 E=-3089.926162H FreqIm=1   \| C \| -2.779620 \| 5.068896 \| 1.993126 \| \| --- \| --- \| --- \| --- \| \| C \| -0.893069 \| 4.000541 \| 0.210768 \| \| C \| -2.152658 \| 3.354467 \| 0.361067 \| \| C \| -0.614671 \| 5.169044 \| 0.921510 \| \| C \| -1.547913 \| 5.694647 \| 1.813802 \| \| P \| -2.895591 \| -1.150593 \| -0.351733 \| \| P \| 0.260881 \| 3.343541 \| -1.065994 \| \| C \| -1.216610 \| 1.071305 \| -0.624867 \| \| C \| -3.912328 \| 1.403920 \| -0.503337 \| \| C \| -1.336519 \| -0.317943 \| -0.564656 \| \| C \| 2.360257 \| 5.109185 \| -0.829812 \| \| H \| 1.611046 \| 5.847940 \| -1.108369 \| \| C \| 1.985749 \| 3.770034 \| -0.639352 \| \| C \| 0.104158 \| 1.580192 \| -0.687400 \| \| C \| -4.978298 \| 2.282352 \| -0.754314 \| \| H \| -4.783366 \| 3.351039 \| -0.780037 \| \| C \| 2.970589 \| 2.791793 \| -0.363345 \| \| C \| -4.231248 \| 0.020691 \| -0.526892 \| \| C \| -5.524020 \| -0.452140 \| -0.760862 \| \| H \| -5.719387 \| -1.521002 \| -0.793837 \| \| C \| -6.272882 \| 1.823414 \| -0.998518 \| \| B \| -2.445491 \| 1.971017 \| -0.303161 \| \| C \| 3.688733 \| 5.502375 \| -0.712535 \| \| H \| 3.958537 \| 6.545146 \| -0.853796 \| \| C \| 4.673042 \| 4.547673 \| -0.456988 \| \| C \| 4.309638 \| 3.216134 \| -0.293276 \| \| H \| 5.082132 \| 2.469205 \| -0.125450 \| \| C \| -6.552288 \| 0.459127 \| -0.991179 \| \| H \| -7.559080 \| 0.102135 \| -1.183591 \| \| B \| 2.628093 \| 1.277946 \| -0.156433 \| \| C \| 1.212110 \| 0.730119 \| -0.567893 \| \| C \| 1.371347 \| -6.133248 \| -0.484076 \| \| C \| 1.959690 \| -3.417569 \| -0.435597 \| \| C \| 0.602473 \| -3.808415 \| -0.419134 \| \| C \| 0.349536 \| -5.188673 \| -0.446566 \| \| H \| -0.686018 \| -5.523799 \| -0.445073 \| \| C \| 2.996276 \| -4.362255 \| -0.501600 \| \| H \| 4.030944 \| -4.031294 \| -0.573154 \| \| C \| 2.703869 \| -5.720624 \| -0.508731 \| \| H \| 3.507267 \| -6.449477 \| -0.556179 \| \| P \| 2.458855 \| -1.686855 \| -0.566553 \| \| C \| -0.281808 \| -1.236002 \| -0.562037 \| \| C \| -1.508671 \| -2.890033 \| 1.058280 \| \| C \| 3.927993 \| -1.821045 \| 1.850105 \| \| H \| 3.853809 \| -2.904448 \| 1.794389 \| \| C \| 3.325189 \| -1.022012 \| 0.881380 \| \| C \| 0.984818 \| -0.663551 \| -0.561336 \| \| C \| -1.195912 \| -3.661635 \| 2.180067 \| \| H \| -0.300675 \| -4.279328 \| 2.166905 \| \| C \| 3.397547 \| 0.391082 \| 0.916787 \| \| C \| -2.680264 \| -2.110509 \| 1.155150 \| \| C \| -3.518527 \| -2.090339 \| 2.265864 \| \| H \| -4.411494 \| -1.469768 \| 2.283828 \| \| C \| -2.014724 \| -3.657248 \| 3.313290 \| \| B \| -0.645914 \| -2.801011 \| -0.344310 \| \| C \| 4.601602 \| -1.217378 \| 2.911804 \| \| H \| 5.060724 \| -1.832857 \| 3.680027 \| \| C \| 4.669787 \| 0.171322 \| 2.989956 \| \| C \| 4.075109 \| 0.959780 \| 2.003973 \| \| H \| 4.126233 \| 2.042140 \| 2.090290 \| \| C \| -3.172576 \| -2.881061 \| 3.360985 \| \| H \| -3.800676 \| -2.886788 \| 4.246487 \| \| C \| -3.069451 \| 3.916182 \| 1.270229 \| \| H \| -4.021438 \| 3.418384 \| 1.437164 \| \| H \| 0.349856 \| 5.656976 \| 0.814614 \| \| H \| -1.301961 \| 6.587956 \| 2.381363 \| \| O \| -3.668407 \| -2.743456 \| -2.426246 \| \| C \| -2.780253 \| -2.547758 \| -1.637003 \| \| O \| -1.670577 \| -3.192136 \| -1.472040 \| \| O \| 3.698383 \| 0.317681 \| -1.935030 \| \| C \| 3.703923 \| -0.872917 \| -2.183600 \| \| O \| 4.116411 \| -1.726425 \| -2.907757 \| \| H \| -7.066397 \| 2.536904 \| -1.200524 \| \| H \| 5.717718 \| 4.839646 \| -0.402867 \| \| H \| -3.503061 \| 5.470977 \| 2.695880 \| \| H \| 5.184335 \| 0.643520 \| 3.821904 \| \| H \| -1.746257 \| -4.268763 \| 4.170381 \| \| H \| 1.130373 \| -7.192468 \| -0.507496 \| |
| 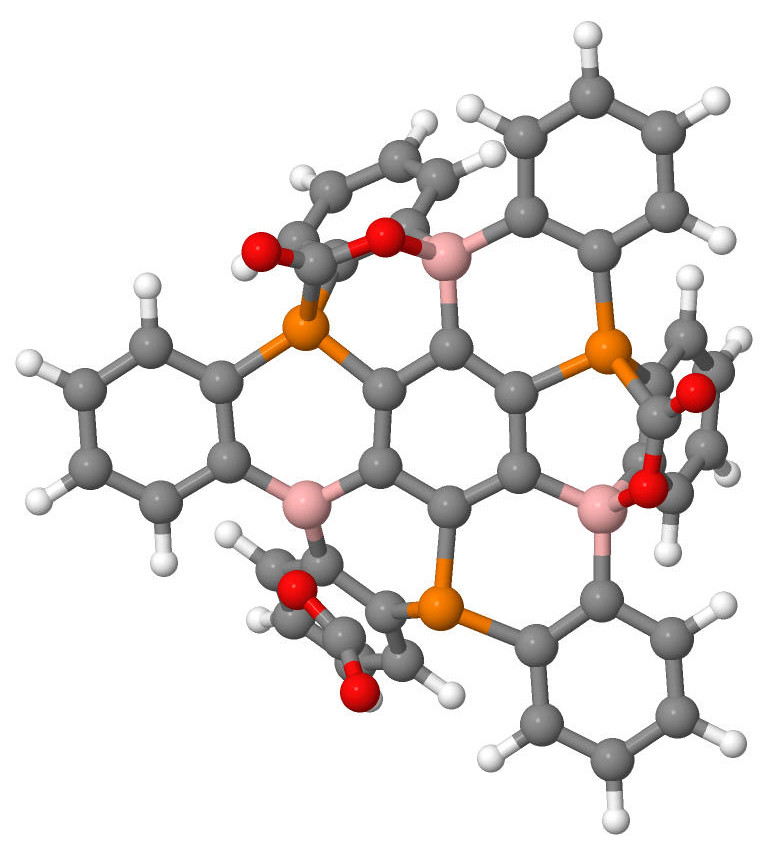 | 2CO2-B3P3-NG/CO2 E=-3278.458262H FreqIm=1   \| C \| -4.718180 \| -1.734758 \| 2.844082 \| \| --- \| --- \| --- \| --- \| \| C \| -3.690547 \| -0.106937 \| 0.832181 \| \| C \| -3.279823 \| -1.465138 \| 0.890315 \| \| C \| -4.605080 \| 0.413226 \| 1.745492 \| \| C \| -5.112124 \| -0.402157 \| 2.758156 \| \| P \| 0.858178 \| -3.007128 \| -0.299797 \| \| P \| -3.051690 \| 0.842663 \| -0.589553 \| \| C \| -1.042829 \| -0.967779 \| -0.435586 \| \| C \| -1.858479 \| -3.551040 \| -0.290578 \| \| C \| 0.294740 \| -1.336161 \| -0.470635 \| \| C \| -4.456262 \| 3.187743 \| -0.479968 \| \| H \| -5.319880 \| 2.527396 \| -0.540606 \| \| C \| -3.161201 \| 2.646678 \| -0.433739 \| \| C \| -1.317037 \| 0.410314 \| -0.514147 \| \| C \| -2.921896 \| -4.453295 \| -0.429434 \| \| H \| -3.937346 \| -4.064939 \| -0.407059 \| \| C \| -2.014448 \| 3.477361 \| -0.434318 \| \| C \| -0.552858 \| -4.107952 \| -0.358576 \| \| C \| -0.331360 \| -5.476470 \| -0.542334 \| \| H \| 0.682550 \| -5.862698 \| -0.613947 \| \| C \| -2.712887 \| -5.818391 \| -0.620912 \| \| B \| -2.168256 \| -2.004307 \| -0.097330 \| \| C \| -4.644334 \| 4.564363 \| -0.480869 \| \| H \| -5.648560 \| 4.976356 \| -0.510547 \| \| C \| -3.531147 \| 5.404838 \| -0.474135 \| \| C \| -2.249757 \| 4.861292 \| -0.458410 \| \| H \| -1.389435 \| 5.528077 \| -0.470577 \| \| C \| -1.419380 \| -6.334856 \| -0.669556 \| \| H \| -1.255995 \| -7.396928 \| -0.822537 \| \| B \| -0.486401 \| 2.969035 \| -0.386082 \| \| C \| -0.311170 \| 1.369428 \| -0.542039 \| \| C \| 6.417523 \| 0.492247 \| -0.967274 \| \| C \| 3.857564 \| 1.472768 \| -0.607194 \| \| C \| 4.028784 \| 0.064198 \| -0.623792 \| \| C \| 5.345387 \| -0.382764 \| -0.810353 \| \| H \| 5.523976 \| -1.455517 \| -0.842961 \| \| C \| 4.926876 \| 2.365868 \| -0.771264 \| \| H \| 4.746167 \| 3.438517 \| -0.785918 \| \| C \| 6.213299 \| 1.872414 \| -0.945938 \| \| H \| 7.045075 \| 2.556833 \| -1.079232 \| \| P \| 2.216861 \| 2.174740 \| -0.448661 \| \| C \| 1.370005 \| -0.446467 \| -0.534904 \| \| C \| 2.923500 \| -1.914217 \| 0.960578 \| \| C \| 2.539114 \| 3.368268 \| 2.117615 \| \| H \| 3.590638 \| 3.090159 \| 2.112890 \| \| C \| 1.715104 \| 3.064579 \| 1.038444 \| \| C \| 1.003985 \| 0.884584 \| -0.557170 \| \| C \| 3.824588 \| -1.742104 \| 2.012832 \| \| H \| 4.579457 \| -0.961528 \| 1.943630 \| \| C \| 0.341902 \| 3.390842 \| 0.976554 \| \| C \| 1.968602 \| -2.944265 \| 1.123502 \| \| C \| 1.905706 \| -3.773316 \| 2.238718 \| \| H \| 1.150533 \| -4.552294 \| 2.314605 \| \| C \| 3.779331 \| -2.557559 \| 3.148186 \| \| B \| 2.863677 \| -1.044055 \| -0.434829 \| \| C \| 1.976911 \| 4.026663 \| 3.211275 \| \| H \| 2.592363 \| 4.275040 \| 4.070556 \| \| C \| 0.622418 \| 4.356282 \| 3.195782 \| \| C \| -0.181790 \| 4.041749 \| 2.095977 \| \| H \| -1.235596 \| 4.311311 \| 2.108793 \| \| C \| 2.829212 \| -3.570610 \| 3.264741 \| \| H \| 2.802432 \| -4.198194 \| 4.150171 \| \| C \| -3.813378 \| -2.254116 \| 1.917745 \| \| H \| -3.504450 \| -3.293508 \| 2.003957 \| \| H \| -4.904510 \| 1.456831 \| 1.688481 \| \| H \| -5.809239 \| 0.009661 \| 3.482217 \| \| O \| 2.172125 \| -4.008125 \| -2.478877 \| \| C \| 2.166492 \| -3.107509 \| -1.681648 \| \| O \| 2.991572 \| -2.109245 \| -1.575278 \| \| O \| 0.314731 \| 3.627637 \| -1.562041 \| \| C \| 1.579351 \| 3.403040 \| -1.757156 \| \| O \| 2.306679 \| 3.870724 \| -2.594030 \| \| O \| -3.339586 \| -1.593724 \| -1.922336 \| \| C \| -3.815614 \| -0.541376 \| -2.285484 \| \| O \| -4.470151 \| 0.058669 \| -3.066918 \| \| H \| -3.663574 \| 6.483040 \| -0.494647 \| \| H \| -5.111086 \| -2.368861 \| 3.633419 \| \| H \| -3.564590 \| -6.482066 \| -0.738071 \| \| H \| 4.495374 \| -2.400755 \| 3.950188 \| \| H \| 7.418707 \| 0.096953 \| -1.115125 \| \| H \| 0.185808 \| 4.865097 \| 4.050926 \| |
| 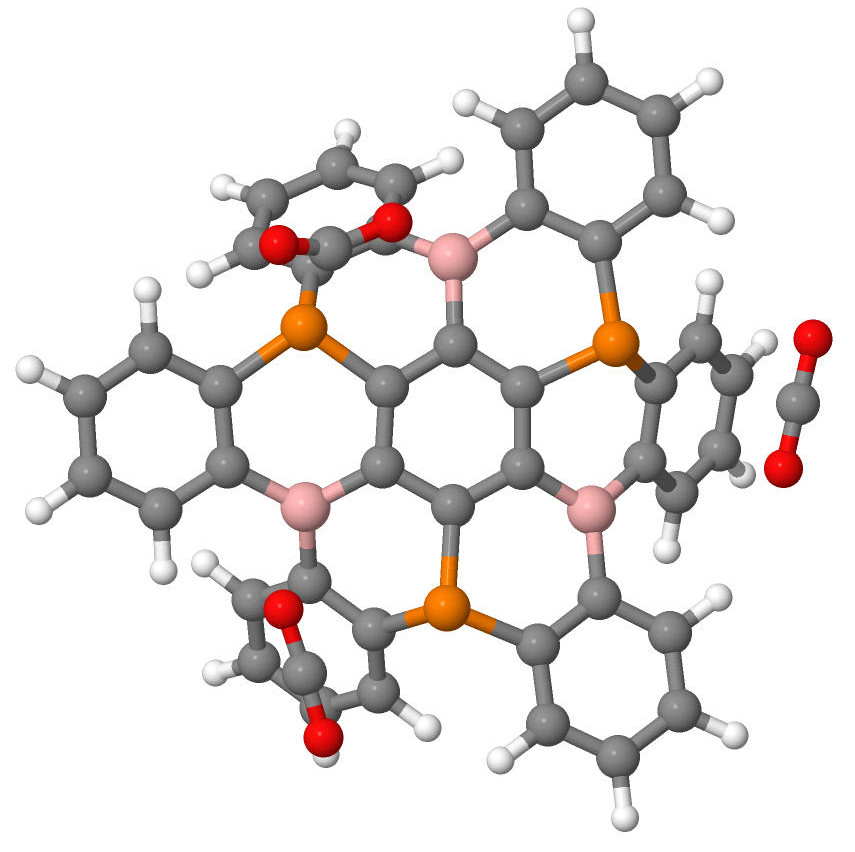 | B3P3-NG/1CO2:2CO2 E=-3278.4486297H FreqIm=1   \| C \| -4.701846 \| 2.487895 \| 2.671005 \| \| --- \| --- \| --- \| --- \| \| C \| -2.716953 \| 2.695822 \| 0.712088 \| \| C \| -3.404652 \| 1.464159 \| 0.865644 \| \| C \| -3.049603 \| 3.801108 \| 1.496324 \| \| C \| -4.035391 \| 3.695196 \| 2.476499 \| \| P \| -1.739554 \| -2.713181 \| -0.416751 \| \| P \| -1.493280 \| 2.798818 \| -0.655915 \| \| C \| -1.480381 \| 0.079062 \| -0.356784 \| \| C \| -3.954797 \| -1.040330 \| -0.125209 \| \| C \| -0.838702 \| -1.161785 \| -0.365602 \| \| C \| -0.698783 \| 5.420760 \| -0.448477 \| \| H \| -1.741286 \| 5.631237 \| -0.681531 \| \| C \| -0.273610 \| 4.094554 \| -0.285967 \| \| C \| -0.637676 \| 1.225236 \| -0.401644 \| \| C \| -5.344465 \| -0.843274 \| -0.200909 \| \| H \| -5.736149 \| 0.167424 \| -0.123832 \| \| C \| 1.092923 \| 3.801041 \| -0.057424 \| \| C \| -3.496426 \| -2.369515 \| -0.287441 \| \| C \| -4.378942 \| -3.434917 \| -0.496057 \| \| H \| -3.992995 \| -4.439077 \| -0.658231 \| \| C \| -6.230499 \| -1.898903 \| -0.399744 \| \| B \| -2.980473 \| 0.180591 \| 0.062624 \| \| C \| 0.207216 \| 6.471876 \| -0.356875 \| \| H \| -0.137986 \| 7.495239 \| -0.473628 \| \| C \| 1.562102 \| 6.202873 \| -0.161504 \| \| C \| 1.990136 \| 4.887637 \| -0.023342 \| \| H \| 3.052196 \| 4.686378 \| 0.088428 \| \| C \| -5.750233 \| -3.200661 \| -0.534058 \| \| H \| -6.436771 \| -4.026156 \| -0.695665 \| \| B \| 1.622303 \| 2.337914 \| 0.076044 \| \| C \| 0.759844 \| 1.124277 \| -0.394239 \| \| C \| 4.564115 \| -4.582859 \| -0.572019 \| \| C \| 3.636249 \| -1.941534 \| -0.557923 \| \| C \| 2.719081 \| -2.994109 \| -0.343212 \| \| C \| 3.216518 \| -4.309134 \| -0.369220 \| \| H \| 2.516608 \| -5.132603 \| -0.245402 \| \| C \| 4.993343 \| -2.220511 \| -0.782794 \| \| H \| 5.684230 \| -1.408528 \| -1.002975 \| \| C \| 5.459699 \| -3.530375 \| -0.765371 \| \| H \| 6.514184 \| -3.730813 \| -0.932870 \| \| P \| 3.119770 \| -0.211359 \| -0.821579 \| \| C \| 0.564033 \| -1.333376 \| -0.405884 \| \| C \| 0.390430 \| -3.613057 \| 0.972278 \| \| C \| 4.803898 \| 0.486988 \| 1.359812 \| \| H \| 5.358621 \| -0.424683 \| 1.154959 \| \| C \| 3.666459 \| 0.800927 \| 0.615435 \| \| C \| 1.337663 \| -0.175089 \| -0.473676 \| \| C \| 1.020124 \| -4.314635 \| 2.008493 \| \| H \| 2.105476 \| -4.313225 \| 2.067671 \| \| C \| 2.942625 \| 1.995188 \| 0.862937 \| \| C \| -1.022177 \| -3.617931 \| 0.973109 \| \| C \| -1.771028 \| -4.312724 \| 1.919528 \| \| H \| -2.857907 \| -4.296823 \| 1.885530 \| \| C \| 0.285134 \| -5.002369 \| 2.975678 \| \| B \| 1.195243 \| -2.741635 \| -0.092131 \| \| C \| 5.217106 \| 1.324615 \| 2.396149 \| \| H \| 6.091725 \| 1.059974 \| 2.984020 \| \| C \| 4.497894 \| 2.480428 \| 2.688787 \| \| C \| 3.378075 \| 2.805047 \| 1.925756 \| \| H \| 2.814361 \| 3.700439 \| 2.174374 \| \| C \| -1.107177 \| -5.007672 \| 2.930262 \| \| H \| -1.678099 \| -5.541707 \| 3.684201 \| \| C \| -4.386176 \| 1.393081 \| 1.870433 \| \| H \| -4.896032 \| 0.449381 \| 2.045966 \| \| H \| -2.518675 \| 4.740928 \| 1.373723 \| \| H \| -4.268264 \| 4.555075 \| 3.098393 \| \| O \| -1.751968 \| -4.231343 \| -2.818661 \| \| C \| -0.907634 \| -3.811650 \| -2.083925 \| \| O \| 0.290356 \| -3.772735 \| -1.863604 \| \| H \| -7.297368 \| -1.704882 \| -0.460991 \| \| H \| -5.458925 \| 2.397954 \| 3.444293 \| \| H \| 2.281981 \| 7.015584 \| -0.134454 \| \| H \| 4.805644 \| 3.125045 \| 3.506840 \| \| H \| 4.915410 \| -5.610420 \| -0.593799 \| \| H \| 0.802572 \| -5.534504 \| 3.768743 \| \| O \| 4.814666 \| 2.962143 \| -1.630804 \| \| C \| 5.515693 \| 2.035617 \| -1.591116 \| \| O \| 6.233610 \| 1.119780 \| -1.559404 \| \| O \| -4.500245 \| 3.843046 \| -2.099964 \| \| C \| -4.349499 \| 2.691937 \| -2.152128 \| \| O \| -4.225696 \| 1.534960 \| -2.209910 \| |
| 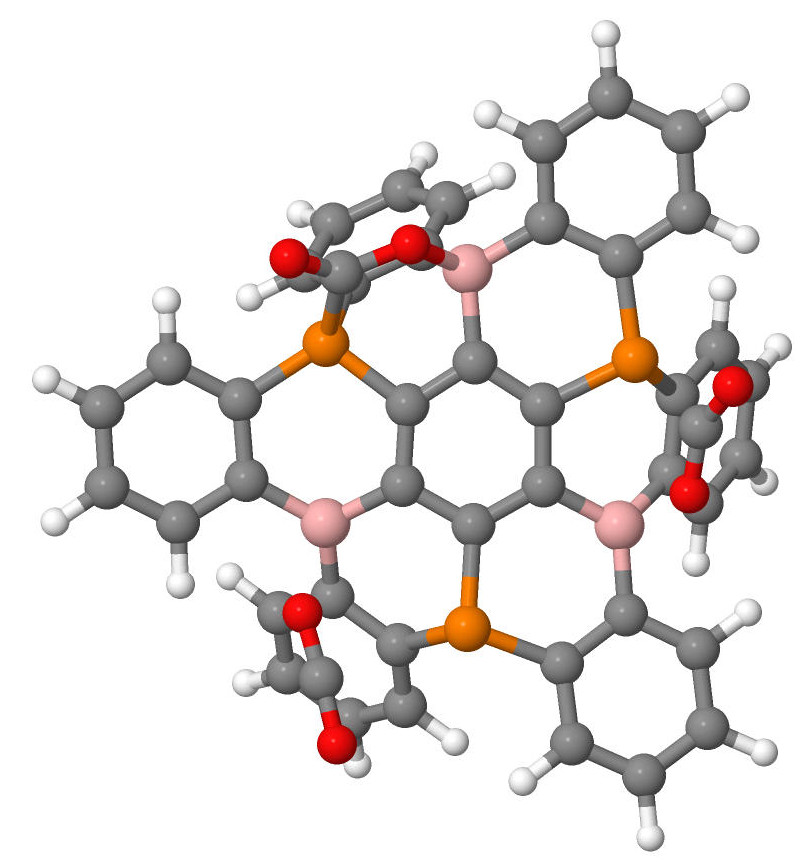 | 1CO2-B3P3-NG/CO2:CO2 E=-3278.451065H FreqIm=1   \| C \| -5.289386 \| 0.184284 \| 2.677451 \| \| --- \| --- \| --- \| --- \| \| C \| -3.575497 \| 1.272435 \| 0.743261 \| \| C \| -3.657984 \| -0.141701 \| 0.881295 \| \| C \| -4.364117 \| 2.105087 \| 1.539386 \| \| C \| -5.209447 \| 1.564487 \| 2.507145 \| \| P \| -0.272059 \| -3.092669 \| -0.194999 \| \| P \| -2.537206 \| 1.939195 \| -0.624185 \| \| C \| -1.325582 \| -0.505250 \| -0.342103 \| \| C \| -2.987094 \| -2.646085 \| -0.090361 \| \| C \| -0.198329 \| -1.325035 \| -0.395084 \| \| C \| -2.943981 \| 4.645123 \| -0.340728 \| \| H \| -3.984547 \| 4.385575 \| -0.527198 \| \| C \| -1.976637 \| 3.633328 \| -0.243786 \| \| C \| -1.082446 \| 0.888133 \| -0.406711 \| \| C \| -4.306614 \| -3.106658 \| -0.217913 \| \| H \| -5.119378 \| -2.386873 \| -0.170408 \| \| C \| -0.610098 \| 3.967499 \| -0.090779 \| \| C \| -1.973593 \| -3.631539 \| -0.209666 \| \| C \| -2.255547 \| -4.983843 \| -0.417909 \| \| H \| -1.446903 \| -5.701987 \| -0.527985 \| \| C \| -4.602962 \| -4.451928 \| -0.433246 \| \| B \| -2.700577 \| -1.097162 \| 0.091022 \| \| C \| -2.583746 \| 5.984929 \| -0.248260 \| \| H \| -3.346072 \| 6.755798 \| -0.316456 \| \| C \| -1.239578 \| 6.332301 \| -0.112452 \| \| C \| -0.276107 \| 5.332689 \| -0.042399 \| \| H \| 0.773600 \| 5.607470 \| 0.031147 \| \| C \| -3.581621 \| -5.395274 \| -0.522639 \| \| H \| -3.812274 \| -6.441976 \| -0.693810 \| \| B \| 0.522171 \| 2.888944 \| 0.000668 \| \| C \| 0.222601 \| 1.398655 \| -0.404126 \| \| C \| 6.158432 \| -2.010548 \| -0.926784 \| \| C \| 4.153344 \| -0.104452 \| -0.663518 \| \| C \| 3.787838 \| -1.468424 \| -0.632008 \| \| C \| 4.830463 \| -2.397712 \| -0.770571 \| \| H \| 4.581936 \| -3.457359 \| -0.760457 \| \| C \| 5.486209 \| 0.295778 \| -0.849184 \| \| H \| 5.731009 \| 1.353495 \| -0.929585 \| \| C \| 6.491842 \| -0.656220 \| -0.963599 \| \| H \| 7.522507 \| -0.344391 \| -1.102989 \| \| P \| 2.923884 \| 1.218786 \| -0.663272 \| \| C \| 1.127493 \| -0.894900 \| -0.513144 \| \| C \| 2.059673 \| -2.819169 \| 1.000617 \| \| C \| 4.023609 \| 2.380888 \| 1.672188 \| \| H \| 4.902912 \| 1.758345 \| 1.525109 \| \| C \| 2.939787 \| 2.287151 \| 0.802270 \| \| C \| 1.293441 \| 0.484735 \| -0.511871 \| \| C \| 2.985530 \| -2.962203 \| 2.036849 \| \| H \| 3.971020 \| -2.513417 \| 1.934246 \| \| C \| 1.776772 \| 3.078840 \| 0.958747 \| \| C \| 0.801627 \| -3.421406 \| 1.211423 \| \| C \| 0.459825 \| -4.142347 \| 2.351678 \| \| H \| -0.526599 \| -4.587520 \| 2.459334 \| \| C \| 2.669143 \| -3.674894 \| 3.197215 \| \| B \| 2.293552 \| -2.017910 \| -0.421202 \| \| C \| 3.958363 \| 3.258521 \| 2.754310 \| \| H \| 4.792732 \| 3.325395 \| 3.446515 \| \| C \| 2.818547 \| 4.034087 \| 2.950636 \| \| C \| 1.746098 \| 3.942400 \| 2.062452 \| \| H \| 0.859100 \| 4.545107 \| 2.240433 \| \| C \| 1.416671 \| -4.267140 \| 3.358296 \| \| H \| 1.183163 \| -4.818925 \| 4.263649 \| \| C \| -4.522227 \| -0.649228 \| 1.868864 \| \| H \| -4.572922 \| -1.723887 \| 2.025529 \| \| H \| -4.295399 \| 3.184366 \| 1.436911 \| \| H \| -5.794613 \| 2.227484 \| 3.138404 \| \| O \| 0.489306 \| -4.549614 \| -2.371626 \| \| C \| 0.854460 \| -3.698269 \| -1.602448 \| \| O \| 1.990673 \| -3.081988 \| -1.538560 \| \| H \| -5.637913 \| -4.764229 \| -0.538095 \| \| H \| -5.938752 \| -0.238322 \| 3.438191 \| \| H \| -0.945721 \| 7.377301 \| -0.078798 \| \| H \| 2.761610 \| 4.710516 \| 3.798611 \| \| H \| 6.934261 \| -2.763784 \| -1.033192 \| \| H \| 3.410413 \| -3.770596 \| 3.985909 \| \| O \| 1.727625 \| 3.331765 \| -1.902749 \| \| C \| 2.722900 \| 2.726145 \| -2.251176 \| \| O \| 3.597370 \| 2.647729 \| -3.058717 \| \| O \| -5.406301 \| 1.332432 \| -2.677682 \| \| C \| -4.720932 \| 0.419004 \| -2.469730 \| \| O \| -4.061806 \| -0.522858 \| -2.272162 \| |

**Table S4:** Geometries, energies (in Hartree) and Cartesian coordinates (in Å) of the adducts in vacuum. Level of theory : M06-2X/6-31+G*

| 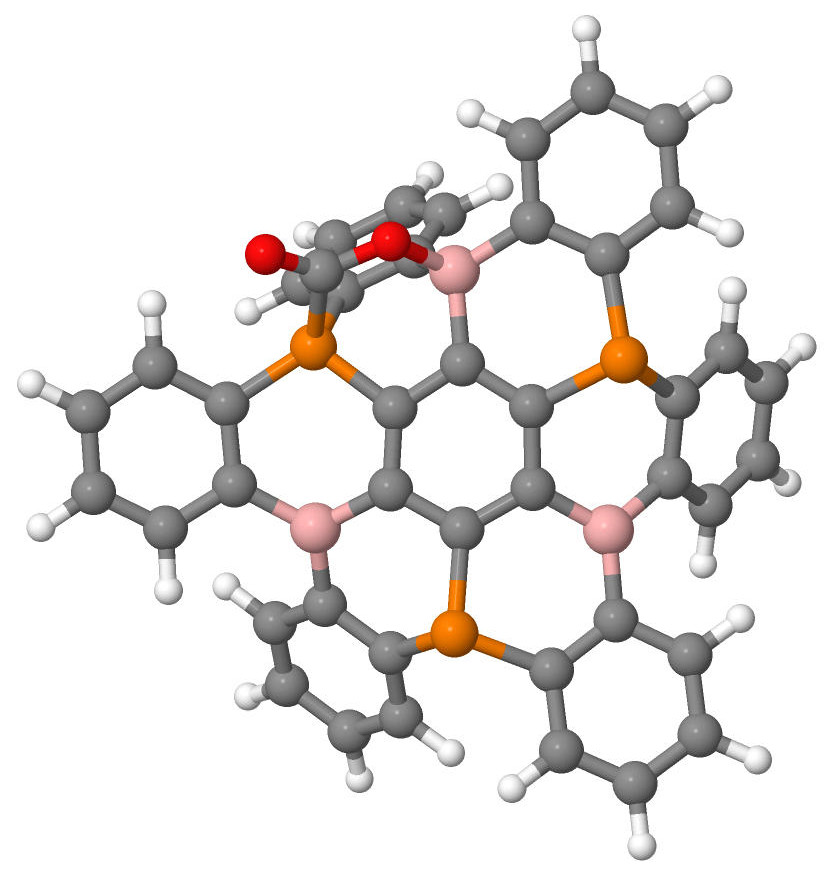 | 1CO2-B3P3-NG E=-2901.4185H FreqIm=0   \| C \| 4.952741 \| -0.363728 \| 2.473709 \| \| --- \| --- \| --- \| --- \| \| C \| 3.511658 \| -1.265550 \| 0.248327 \| \| C \| 3.538370 \| 0.124545 \| 0.535278 \| \| C \| 4.224672 \| -2.166688 \| 1.040259 \| \| C \| 4.939472 \| -1.718044 \| 2.150148 \| \| P \| 0.294024 \| 3.245517 \| -1.036887 \| \| P \| 2.594721 \| -1.793864 \| -1.260718 \| \| C \| 1.299090 \| 0.607613 \| -0.816855 \| \| C \| 2.980969 \| 2.681722 \| -0.302342 \| \| C \| 0.174724 \| 1.452085 \| -0.831188 \| \| C \| 3.083090 \| -4.491442 \| -1.178238 \| \| H \| 4.113686 \| -4.167028 \| -1.315165 \| \| C \| 2.061715 \| -3.534617 \| -1.044764 \| \| C \| 1.104909 \| -0.794746 \| -0.966667 \| \| C \| 4.314222 \| 3.119794 \| -0.169604 \| \| H \| 5.096986 \| 2.378318 \| -0.034450 \| \| C \| 0.714136 \| -3.941175 \| -0.965541 \| \| C \| 1.995492 \| 3.670736 \| -0.545921 \| \| C \| 2.356376 \| 5.023175 \| -0.633769 \| \| H \| 1.604414 \| 5.769583 \| -0.882489 \| \| C \| 4.663847 \| 4.463551 \| -0.231565 \| \| B \| 2.650688 \| 1.153069 \| -0.255900 \| \| C \| 2.796577 \| -5.851088 \| -1.172267 \| \| H \| 3.599962 \| -6.576527 \| -1.264711 \| \| C \| 1.469014 \| -6.270535 \| -1.081424 \| \| C \| 0.455789 \| -5.322285 \| -0.991495 \| \| H \| -0.580163 \| -5.654608 \| -0.943841 \| \| C \| 3.675196 \| 5.423035 \| -0.448409 \| \| H \| 3.935082 \| 6.476140 \| -0.507895 \| \| B \| -0.526622 \| -2.942817 \| -0.832144 \| \| C \| -0.168772 \| -1.364627 \| -0.967511 \| \| C \| -6.206152 \| 1.641828 \| -1.153307 \| \| C \| -4.142239 \| -0.164271 \| -0.813773 \| \| C \| -3.843750 \| 1.215329 \| -0.677897 \| \| C \| -4.919896 \| 2.097614 \| -0.864236 \| \| H \| -4.740088 \| 3.167515 \| -0.801702 \| \| C \| -5.426461 \| -0.635514 \| -1.094096 \| \| H \| -5.605146 \| -1.700892 \| -1.214603 \| \| C \| -6.465938 \| 0.277321 \| -1.257685 \| \| H \| -7.466566 \| -0.076574 \| -1.485034 \| \| P \| -2.789721 \| -1.324383 \| -0.717844 \| \| C \| -1.143453 \| 0.929540 \| -0.818748 \| \| C \| -2.123759 \| 3.103070 \| 0.362831 \| \| C \| -3.385432 \| -2.415601 \| 1.846588 \| \| H \| -4.286516 \| -1.809583 \| 1.906860 \| \| C \| -2.559792 \| -2.366536 \| 0.726969 \| \| C \| -1.234726 \| -0.462302 \| -0.875931 \| \| C \| -3.054214 \| 3.568287 \| 1.311101 \| \| H \| -3.997570 \| 3.041134 \| 1.429348 \| \| C \| -1.378585 \| -3.119530 \| 0.572276 \| \| C \| -0.877521 \| 3.780758 \| 0.276810 \| \| C \| -0.621443 \| 4.890313 \| 1.083954 \| \| H \| 0.334444 \| 5.402642 \| 1.024493 \| \| C \| -2.788362 \| 4.661161 \| 2.130276 \| \| B \| -2.384367 \| 1.776855 \| -0.423380 \| \| C \| -3.014151 \| -3.257468 \| 2.893579 \| \| H \| -3.630827 \| -3.320476 \| 3.784952 \| \| C \| -1.843488 \| -4.009771 \| 2.791232 \| \| C \| -1.038742 \| -3.941662 \| 1.650471 \| \| H \| -0.132114 \| -4.539742 \| 1.596239 \| \| C \| -1.568144 \| 5.322728 \| 2.011346 \| \| H \| -1.341541 \| 6.169869 \| 2.652889 \| \| C \| 4.259612 \| 0.537881 \| 1.670495 \| \| H \| 4.252421 \| 1.588531 \| 1.948632 \| \| H \| 4.193968 \| -3.230290 \| 0.822087 \| \| H \| 5.468623 \| -2.434514 \| 2.772704 \| \| H \| 5.702883 \| 4.762015 \| -0.127654 \| \| H \| 5.491982 \| -0.012987 \| 3.348719 \| \| H \| 1.227203 \| -7.329727 \| -1.098488 \| \| H \| -1.553465 \| -4.659529 \| 3.612510 \| \| H \| -7.008937 \| 2.358081 \| -1.302293 \| \| H \| -3.522004 \| 4.989257 \| 2.860414 \| \| C \| -2.676906 \| -2.632586 \| -2.092220 \| \| O \| -1.565562 \| -3.278152 \| -1.976263 \| \| O \| -3.573903 \| -2.778280 \| -2.884060 \| |
| --- | --- | --- | --- | --- | --- | --- | --- | --- | --- | --- | --- | --- | --- | --- | --- | --- | --- | --- | --- | --- | --- | --- | --- | --- | --- | --- | --- | --- | --- | --- | --- | --- | --- | --- | --- | --- | --- | --- | --- | --- | --- | --- | --- | --- | --- | --- | --- | --- | --- | --- | --- | --- | --- | --- | --- | --- | --- | --- | --- | --- | --- | --- | --- | --- | --- | --- | --- | --- | --- | --- | --- | --- | --- | --- | --- | --- | --- | --- | --- | --- | --- | --- | --- | --- | --- | --- | --- | --- | --- | --- | --- | --- | --- | --- | --- | --- | --- | --- | --- | --- | --- | --- | --- | --- | --- | --- | --- | --- | --- | --- | --- | --- | --- | --- | --- | --- | --- | --- | --- | --- | --- | --- | --- | --- | --- | --- | --- | --- | --- | --- | --- | --- | --- | --- | --- | --- | --- | --- | --- | --- | --- | --- | --- | --- | --- | --- | --- | --- | --- | --- | --- | --- | --- | --- | --- | --- | --- | --- | --- | --- | --- | --- | --- | --- | --- | --- | --- | --- | --- | --- | --- | --- | --- | --- | --- | --- | --- | --- | --- | --- | --- | --- | --- | --- | --- | --- | --- | --- | --- | --- | --- | --- | --- | --- | --- | --- | --- | --- | --- | --- | --- | --- | --- | --- | --- | --- | --- | --- | --- | --- | --- | --- | --- | --- | --- | --- | --- | --- | --- | --- | --- | --- | --- | --- | --- | --- | --- | --- | --- | --- | --- | --- | --- | --- | --- | --- | --- | --- | --- | --- | --- | --- | --- | --- | --- | --- | --- | --- | --- | --- | --- | --- | --- | --- | --- | --- | --- | --- | --- | --- | --- | --- | --- | --- | --- | --- | --- | --- | --- | --- | --- | --- | --- | --- | --- | --- | --- | --- | --- | --- | --- | --- | --- | --- | --- | --- | --- | --- | --- | --- | --- | --- | --- | --- | --- | --- | --- | --- | --- | --- | --- |
| 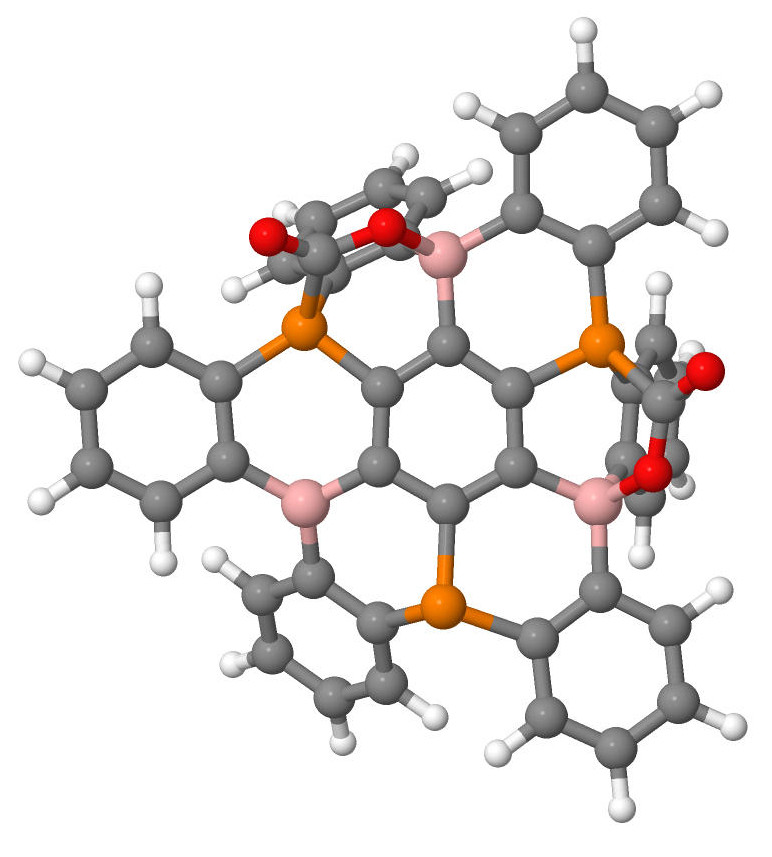 | 2CO2-B3P3-NG E=-3089.947836H FreqIm=0   \| C \| 1.191862 \| 5.532423 \| 1.990475 \| \| --- \| --- \| --- \| --- \| \| C \| 1.915692 \| 3.543629 \| 0.137518 \| \| C \| 0.539281 \| 3.874849 \| 0.309093 \| \| C \| 2.890476 \| 4.224524 \| 0.870750 \| \| C \| 2.531923 \| 5.202687 \| 1.796203 \| \| P \| -2.961706 \| 0.943266 \| -0.373385 \| \| P \| 2.367633 \| 2.341973 \| -1.188201 \| \| C \| -0.245527 \| 1.566041 \| -0.729859 \| \| C \| -2.090130 \| 3.549970 \| -0.505468 \| \| C \| -1.245672 \| 0.592223 \| -0.658290 \| \| C \| 5.099853 \| 2.308943 \| -0.900511 \| \| H \| 4.999620 \| 3.369813 \| -1.123948 \| \| C \| 3.951581 \| 1.505641 \| -0.786592 \| \| C \| 1.089201 \| 1.106092 \| -0.846237 \| \| C \| -2.341383 \| 4.913586 \| -0.724528 \| \| H \| -1.501368 \| 5.602078 \| -0.759882 \| \| C \| 4.075557 \| 0.108688 \| -0.614694 \| \| C \| -3.231599 \| 2.703124 \| -0.511564 \| \| C \| -4.528600 \| 3.184781 \| -0.699803 \| \| H \| -5.370947 \| 2.497813 \| -0.719574 \| \| C \| -3.631189 \| 5.406867 \| -0.923745 \| \| B \| -0.593918 \| 3.036645 \| -0.357619 \| \| C \| 6.372139 \| 1.764580 \| -0.775588 \| \| H \| 7.246935 \| 2.403814 \| -0.854613 \| \| C \| 6.512478 \| 0.389823 \| -0.586543 \| \| C \| 5.377718 \| -0.411547 \| -0.522454 \| \| H \| 5.493839 \| -1.487459 \| -0.401454 \| \| C \| -4.727749 \| 4.548814 \| -0.901011 \| \| H \| -5.730844 \| 4.932430 \| -1.058211 \| \| B \| 2.848978 \| -0.916609 \| -0.506247 \| \| C \| 1.388446 \| -0.257227 \| -0.753223 \| \| C \| -2.784200 \| -5.632731 \| -0.717834 \| \| C \| -0.638524 \| -3.903927 \| -0.500392 \| \| C \| -1.939176 \| -3.342130 \| -0.524040 \| \| C \| -2.995314 \| -4.258575 \| -0.635835 \| \| H \| -4.011615 \| -3.871820 \| -0.668994 \| \| C \| -0.409772 \| -5.285041 \| -0.590336 \| \| H \| 0.606930 \| -5.671404 \| -0.603623 \| \| C \| -1.489281 \| -6.152776 \| -0.693712 \| \| H \| -1.321855 \| -7.222549 \| -0.769155 \| \| P \| 0.793435 \| -2.832438 \| -0.421326 \| \| C \| -1.032708 \| -0.785909 \| -0.648391 \| \| C \| -2.966925 \| -1.308308 \| 1.020351 \| \| C \| 1.600380 \| -3.480454 \| 2.231947 \| \| H \| 0.826590 \| -4.243275 \| 2.282690 \| \| C \| 1.783231 \| -2.722972 \| 1.079014 \| \| C \| 0.303989 \| -1.140846 \| -0.677643 \| \| C \| -3.191969 \| -2.113303 \| 2.138412 \| \| H \| -2.906352 \| -3.162727 \| 2.104171 \| \| C \| 2.760132 \| -1.714601 \| 0.937854 \| \| C \| -3.359422 \| 0.043010 \| 1.139111 \| \| C \| -3.956891 \| 0.581856 \| 2.274001 \| \| H \| -4.242042 \| 1.630664 \| 2.312990 \| \| C \| -3.783457 \| -1.596342 \| 3.295493 \| \| B \| -2.290494 \| -1.769414 \| -0.406833 \| \| C \| 2.429167 \| -3.223874 \| 3.323722 \| \| H \| 2.313194 \| -3.794689 \| 4.240078 \| \| C \| 3.399614 \| -2.226354 \| 3.232536 \| \| C \| 3.561031 \| -1.483168 \| 2.059458 \| \| H \| 4.325604 \| -0.710929 \| 2.015071 \| \| C \| -4.168249 \| -0.258330 \| 3.367581 \| \| H \| -4.626398 \| 0.132417 \| 4.270887 \| \| C \| 0.217203 \| 4.872349 \| 1.250867 \| \| H \| -0.827955 \| 5.112035 \| 1.430449 \| \| H \| 3.938619 \| 3.965841 \| 0.754921 \| \| H \| 3.304969 \| 5.697248 \| 2.378132 \| \| O \| -4.645036 \| 0.211249 \| -2.395550 \| \| C \| -3.809228 \| -0.201931 \| -1.634408 \| \| O \| -3.360298 \| -1.411194 \| -1.493519 \| \| O \| 3.025083 \| -2.052481 \| -1.581776 \| \| C \| 2.186948 \| -3.033722 \| -1.700962 \| \| O \| 2.219256 \| -3.971821 \| -2.455535 \| \| H \| 7.501106 \| -0.054443 \| -0.510509 \| \| H \| 0.910729 \| 6.287859 \| 2.717956 \| \| H \| -3.778347 \| 6.468033 \| -1.102184 \| \| H \| -3.947501 \| -2.246450 \| 4.150479 \| \| H \| -3.633687 \| -6.304018 \| -0.808808 \| \| H \| 4.039839 \| -2.024018 \| 4.087019 \| |
| 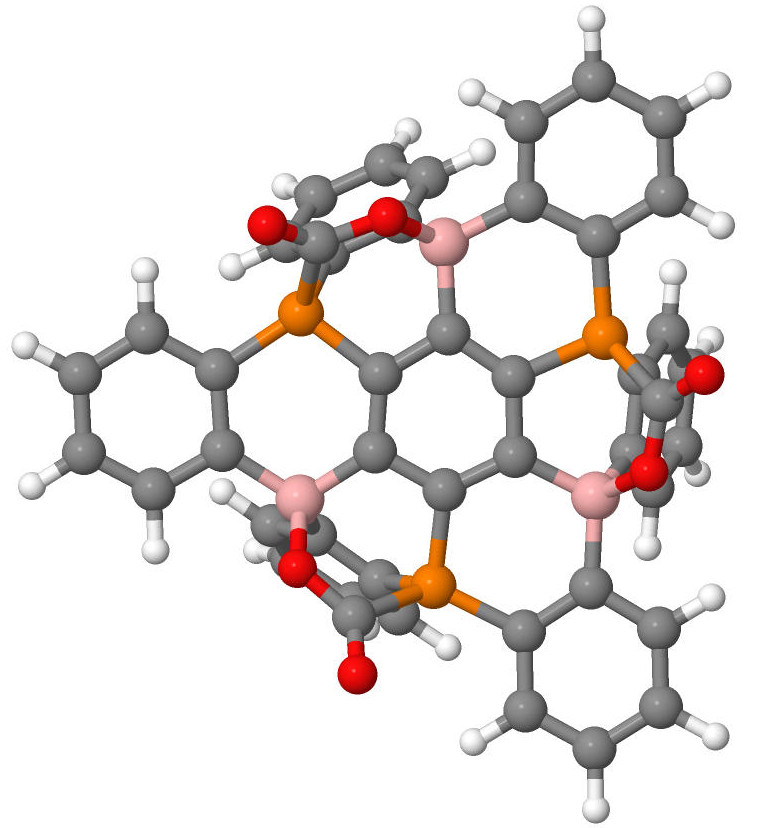 | 3CO2-B3P3-NG E=-3278.485084H FreqIm=0   \| C \| -1.751543 \| -3.910996 \| 3.188300 \| \| --- \| --- \| --- \| --- \| \| C \| -2.487098 \| -2.367639 \| 1.038563 \| \| C \| -1.288725 \| -3.111991 \| 0.934477 \| \| C \| -3.309782 \| -2.384226 \| 2.159890 \| \| C \| -2.930096 \| -3.170138 \| 3.248972 \| \| P \| 2.591205 \| -1.710993 \| -0.455465 \| \| P \| -2.777402 \| -1.388446 \| -0.455909 \| \| C \| -0.147677 \| -1.415181 \| -0.663882 \| \| C \| 0.883337 \| -3.911299 \| -0.586552 \| \| C \| 1.089578 \| -0.804620 \| -0.674677 \| \| C \| -5.423462 \| -0.704761 \| -0.642301 \| \| H \| -5.596236 \| -1.778871 \| -0.642392 \| \| C \| -4.117413 \| -0.200720 \| -0.551484 \| \| C \| -1.241546 \| -0.541152 \| -0.674959 \| \| C \| 0.711752 \| -5.297650 \| -0.715081 \| \| H \| -0.303538 \| -5.686097 \| -0.758625 \| \| C \| -3.828754 \| 1.190936 \| -0.587366 \| \| C \| 2.232764 \| -3.465185 \| -0.551281 \| \| C \| 3.322487 \| -4.343875 \| -0.643172 \| \| H \| 4.338954 \| -3.956091 \| -0.643973 \| \| C \| 1.784668 \| -6.181888 \| -0.801353 \| \| B \| -0.444344 \| -2.984728 \| -0.471691 \| \| C \| -6.492607 \| 0.173448 \| -0.762230 \| \| H \| -7.505391 \| -0.209255 \| -0.838848 \| \| C \| -6.245850 \| 1.546156 \| -0.800956 \| \| C \| -4.943461 \| 2.032883 \| -0.715664 \| \| H \| -4.772029 \| 3.106327 \| -0.759534 \| \| C \| 3.096750 \| -5.708943 \| -0.763432 \| \| H \| 3.934745 \| -6.394367 \| -0.840991 \| \| B \| -2.362561 \| 1.877358 \| -0.472534 \| \| C \| -1.151627 \| 0.835646 \| -0.664354 \| \| C \| 4.461671 \| 4.636441 \| -0.800349 \| \| C \| 1.884831 \| 3.666232 \| -0.551115 \| \| C \| 2.945850 \| 2.720557 \| -0.586598 \| \| C \| 4.232257 \| 3.265138 \| -0.714610 \| \| H \| 5.076380 \| 2.580166 \| -0.757946 \| \| C \| 2.101004 \| 5.049324 \| -0.642582 \| \| H \| 1.256911 \| 5.735687 \| -0.643239 \| \| C \| 3.396073 \| 5.536320 \| -0.762474 \| \| H \| 3.570848 \| 6.604768 \| -0.839579 \| \| P \| 0.186367 \| 3.099596 \| -0.455506 \| \| C \| 1.299636 \| 0.579690 \| -0.663914 \| \| C \| 3.339644 \| 0.440141 \| 0.934428 \| \| C \| -0.410201 \| 4.056801 \| 2.160935 \| \| H \| 0.559501 \| 4.548411 \| 2.193545 \| \| C \| -0.806974 \| 3.337100 \| 1.038903 \| \| C \| 0.152261 \| 1.345944 \| -0.674949 \| \| C \| 3.832646 \| 1.123955 \| 2.046805 \| \| H \| 3.888321 \| 2.210517 \| 2.021298 \| \| C \| -2.050977 \| 2.671895 \| 0.933881 \| \| C \| 3.293752 \| -0.969799 \| 1.038973 \| \| C \| 3.718780 \| -1.673769 \| 2.160656 \| \| H \| 3.659650 \| -2.759391 \| 2.192852 \| \| C \| 4.262584 \| 0.439239 \| 3.188364 \| \| B \| 2.807264 \| 1.107486 \| -0.471872 \| \| C \| -1.281115 \| 4.120374 \| 3.249686 \| \| H \| -0.995819 \| 4.671162 \| 4.140739 \| \| C \| -2.512253 \| 3.470749 \| 3.187979 \| \| C \| -2.889934 \| 2.756493 \| 2.046065 \| \| H \| -3.858700 \| 2.261362 \| 2.020195 \| \| C \| 4.209583 \| -0.951826 \| 3.249539 \| \| H \| 4.544150 \| -1.474663 \| 4.140298 \| \| C \| -0.943260 \| -3.880799 \| 2.046991 \| \| H \| -0.030050 \| -4.472202 \| 2.021805 \| \| H \| -4.220703 \| -1.790698 \| 2.191674 \| \| H \| -3.550529 \| -3.198772 \| 4.139468 \| \| O \| 4.370656 \| -1.492325 \| -2.529973 \| \| C \| 3.719312 \| -0.863814 \| -1.738356 \| \| O \| 3.663202 \| 0.429746 \| -1.586684 \| \| O \| -2.203583 \| 2.957656 \| -1.587053 \| \| C \| -1.111373 \| 3.653066 \| -1.738521 \| \| O \| -0.892926 \| 4.531899 \| -2.529645 \| \| O \| -1.459083 \| -3.387309 \| -1.586576 \| \| C \| -2.607319 \| -2.789119 \| -1.738785 \| \| O \| -3.476890 \| -3.039073 \| -2.530802 \| \| H \| 5.477693 \| 5.007002 \| -0.904560 \| \| H \| 4.643714 \| 0.998701 \| 4.038273 \| \| H \| 1.597660 \| -7.247036 \| -0.906040 \| \| H \| -1.457675 \| -4.520691 \| 4.038326 \| \| H \| -7.074707 \| 2.240811 \| -0.905415 \| \| H \| -3.187545 \| 3.520706 \| 4.037745 \| |
| 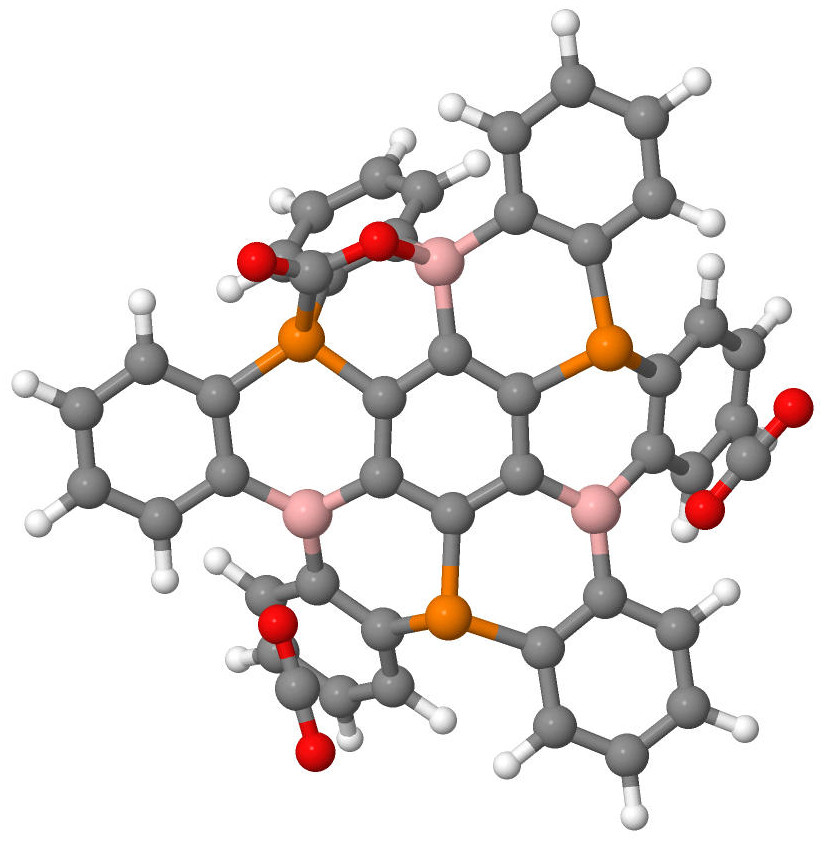 | 1CO2-B3P3-NG:2CO2 E=-3278.4676H FreqIm=0   \| C \| -5.131725 \| 1.807125 \| 2.570325 \| \| --- \| --- \| --- \| --- \| \| C \| -3.062262 \| 2.306774 \| 0.747958 \| \| C \| -3.627782 \| 1.004606 \| 0.813947 \| \| C \| -3.554992 \| 3.332539 \| 1.557036 \| \| C \| -4.578635 \| 3.081634 \| 2.469203 \| \| P \| -1.424155 \| -2.880762 \| -0.292344 \| \| P \| -1.798500 \| 2.619896 \| -0.552641 \| \| C \| -1.510773 \| -0.089912 \| -0.372715 \| \| C \| -3.818757 \| -1.529102 \| -0.265038 \| \| C \| -0.727347 \| -1.243065 \| -0.430779 \| \| C \| -1.283113 \| 5.288313 \| -0.139531 \| \| H \| -2.334759 \| 5.408950 \| -0.393462 \| \| C \| -0.727860 \| 4.002929 \| -0.056380 \| \| C \| -0.791591 \| 1.131403 \| -0.366333 \| \| C \| -5.208917 \| -1.498500 \| -0.456328 \| \| H \| -5.725500 \| -0.544190 \| -0.399692 \| \| C \| 0.656170 \| 3.831109 \| 0.188040 \| \| C \| -3.203295 \| -2.798831 \| -0.397515 \| \| C \| -3.922951 \| -3.962509 \| -0.679344 \| \| H \| -3.406238 \| -4.911532 \| -0.797347 \| \| C \| -5.939927 \| -2.649948 \| -0.745199 \| \| B \| -3.017837 \| -0.188231 \| 0.001525 \| \| C \| -0.494571 \| 6.416364 \| 0.055996 \| \| H \| -0.940459 \| 7.405368 \| 0.000726 \| \| C \| 0.875133 \| 6.270932 \| 0.277046 \| \| C \| 1.431644 \| 4.998758 \| 0.330157 \| \| H \| 2.505558 \| 4.899330 \| 0.463213 \| \| C \| -5.302990 \| -3.885066 \| -0.846923 \| \| H \| -5.872199 \| -4.780603 \| -1.075364 \| \| B \| 1.340882 \| 2.423120 \| 0.223698 \| \| C \| 0.613939 \| 1.160314 \| -0.343334 \| \| C \| 4.981231 \| -4.156355 \| -0.616197 \| \| C \| 3.766413 \| -1.638173 \| -0.557845 \| \| C \| 2.951775 \| -2.786985 \| -0.499881 \| \| C \| 3.598228 \| -4.034171 \| -0.535941 \| \| H \| 2.985904 \| -4.934091 \| -0.504642 \| \| C \| 5.161418 \| -1.759687 \| -0.682411 \| \| H \| 5.773538 \| -0.866901 \| -0.805078 \| \| C \| 5.771156 \| -3.008232 \| -0.687022 \| \| H \| 6.851351 \| -3.085480 \| -0.772460 \| \| P \| 3.113418 \| 0.059337 \| -0.755946 \| \| C \| 0.669253 \| -1.297498 \| -0.508146 \| \| C \| 0.782990 \| -3.439501 \| 1.012856 \| \| C \| 4.615126 \| 0.771434 \| 1.552537 \| \| H \| 5.249654 \| -0.082899 \| 1.334964 \| \| C \| 3.498975 \| 1.038269 \| 0.757427 \| \| C \| 1.321862 \| -0.064790 \| -0.491843 \| \| C \| 1.547239 \| -3.891306 \| 2.092512 \| \| H \| 2.630436 \| -3.805193 \| 2.048547 \| \| C \| 2.662235 \| 2.150997 \| 1.036741 \| \| C \| -0.612882 \| -3.573678 \| 1.153155 \| \| C \| -1.243366 \| -4.133274 \| 2.260863 \| \| H \| -2.327054 \| -4.210754 \| 2.310442 \| \| C \| 0.944545 \| -4.453178 \| 3.221482 \| \| B \| 1.357532 \| -2.763330 \| -0.380055 \| \| C \| 4.897356 \| 1.568546 \| 2.660803 \| \| H \| 5.755363 \| 1.334839 \| 3.285251 \| \| C \| 4.070137 \| 2.642867 \| 2.978721 \| \| C \| 2.971244 \| 2.923605 \| 2.171070 \| \| H \| 2.318032 \| 3.748549 \| 2.443145 \| \| C \| -0.442090 \| -4.580958 \| 3.309931 \| \| H \| -0.897433 \| -5.020646 \| 4.192065 \| \| C \| -4.657808 \| 0.789260 \| 1.747912 \| \| H \| -5.078780 \| -0.207775 \| 1.850442 \| \| H \| -3.118728 \| 4.326093 \| 1.508929 \| \| H \| -4.932266 \| 3.883254 \| 3.111666 \| \| O \| -1.125261 \| -4.488772 \| -2.471993 \| \| C \| -0.517064 \| -3.829310 \| -1.667283 \| \| O \| 0.755633 \| -3.655077 \| -1.537463 \| \| H \| -7.013089 \| -2.581106 \| -0.897780 \| \| H \| -5.921221 \| 1.606865 \| 3.288415 \| \| H \| 1.506847 \| 7.147275 \| 0.388712 \| \| H \| 4.275825 \| 3.254568 \| 3.852242 \| \| H \| 5.442582 \| -5.139821 \| -0.641670 \| \| H \| 1.565466 \| -4.797255 \| 4.044225 \| \| O \| 3.129839 \| 3.242114 \| -1.934052 \| \| C \| 4.234228 \| 2.879518 \| -1.853357 \| \| O \| 5.345742 \| 2.549219 \| -1.777262 \| \| O \| -4.822610 \| 3.268633 \| -2.245637 \| \| C \| -4.490803 \| 2.156011 \| -2.277500 \| \| O \| -4.188437 \| 1.030938 \| -2.317833 \| |

**Table S5 :** Comparison of the M06-2X/6-31+G* and M06-2X/6-311++G(3df,3dp) level of theories for the optimization (RMSD)

*RMSD between the B_3_P_3_-NG structures optimized at the M06-2X/6-31+G* and M06-2X/6-311++G** level of theory*

| **M06-2X/6-31+G*** | **M06-2X/6-311++G**** |
| --- | --- |
| C 3.104363 4.122366 2.362396  C 3.071379 2.250739 0.288977  C 1.935572 3.073770 0.485088  C 4.207135 2.394242 1.085961  C 4.221981 3.327475 2.122183  P -2.429986 2.058057 -1.119606  P 2.997067 1.074825 -1.122133  C 0.299944 1.398888 -0.794885  C -0.443566 3.997822 -0.508033  C -1.023057 0.948782 -0.829302  C 5.502226 -0.046739 -0.965004  H 5.835123 0.958592 -1.216958  C 4.136739 -0.300551 -0.771626  C 1.333104 0.411622 -0.829697  C -0.043128 5.349131 -0.472034  H 1.008247 5.580771 -0.323655  C 3.683706 -1.614775 -0.506143  C -1.808560 3.732663 -0.770629  C -2.711712 4.788106 -0.962683  H -3.749324 4.573340 -1.212095  C -0.944442 6.393069 -0.643771  B 0.608300 2.855337 -0.334216  C 6.436925 -1.072004 -0.873202  H 7.492469 -0.857079 -1.014057  C 6.008183 -2.379291 -0.641503  C 4.653519 -2.637384 -0.468377  H 4.328495 -3.663424 -0.317694  C -2.291328 6.110385 -0.872515  H -3.005755 6.916770 -1.012211  B 2.168503 -1.953717 -0.331243  C 1.061393 -0.959217 -0.794085  C -5.064015 -4.014507 -0.647009  C -2.328040 -3.432597 -0.772570  C -3.240185 -2.383471 -0.508079  C -4.610618 -2.712415 -0.472429  H -5.337099 -1.918015 -0.323467  C -2.790497 -4.741977 -0.967559  H -2.085625 -5.532593 -1.218693  C -4.145798 -5.039228 -0.878073  H -4.487079 -6.060795 -1.019975  P -0.566971 -3.133221 -1.121249  C -1.361362 -0.439670 -0.794431  C -3.629200 0.138381 0.487207  C -0.031599 -4.839113 1.088646  H -1.017216 -5.269032 0.933151  C 0.413012 -3.784441 0.291337  C -0.310039 -1.360390 -0.829626  C -4.446943 -0.281524 1.551123  H -4.536116 -1.343884 1.762448  C 1.693399 -3.211813 0.488695  C -3.483619 1.533697 0.292500  C -4.174089 2.445163 1.091240  H -4.052866 3.513863 0.937666  C -5.120013 0.625670 2.366090  B -2.776715 -0.901478 -0.333173  C 0.767874 -5.317777 2.126197  H 0.403337 -6.123788 2.757176  C 2.014566 -4.746818 2.367763  C 2.464111 -3.709242 1.554300  H 3.427813 -3.254358 1.767789  C -4.989048 1.991123 2.127640  H -5.503913 2.709314 2.759912  C 1.981236 3.991951 1.548971  H 1.105692 4.599841 1.761317  H 5.072231 1.755394 0.931243  H 5.102274 3.415570 2.753040  H -0.599169 7.422352 -0.612742  H 3.104555 4.835339 3.181603  H 6.726856 -3.192966 -0.609136  H 2.631006 -5.102842 3.187951  H -6.128051 -4.230234 -0.616410  H -5.737344 0.268918 3.185265 | C 3.114152 4.135337 2.341896  C 3.069629 2.252631 0.293650  C 1.937865 3.073100 0.490008  C 4.206501 2.405643 1.077444  C 4.226715 3.343981 2.100606  P -2.411610 2.053576 -1.088868  P 2.984308 1.061889 -1.088684  C 0.300561 1.394389 -0.764557  C -0.437615 3.987474 -0.498149  C -1.018333 0.947532 -0.799907  C 5.482585 -0.048075 -0.946424  H 5.812476 0.955299 -1.186627  C 4.122620 -0.302610 -0.753037  C 1.329861 0.408132 -0.799895  C -0.038285 5.333956 -0.475226  H 1.008468 5.565562 -0.332788  C 3.672244 -1.614632 -0.498438  C -1.799048 3.721563 -0.752841  C -2.699470 4.772087 -0.945993  H -3.733348 4.556135 -1.186308  C -0.936709 6.371463 -0.649912  B 0.610155 2.848738 -0.317490  C 6.412764 -1.069820 -0.868205  H 7.463150 -0.854805 -1.008502  C 5.986328 -2.374427 -0.650940  C 4.638645 -2.633739 -0.475998  H 4.315809 -3.656063 -0.333702  C -2.279727 6.088505 -0.867369  H -2.991129 6.890691 -1.007488  B 2.162214 -1.952767 -0.317697  C 1.057400 -0.957507 -0.764623  C -5.049364 -3.997176 -0.650500  C -2.323287 -3.419045 -0.752684  C -3.234379 -2.372896 -0.498544  C -4.600135 -2.700326 -0.476011  H -5.324123 -1.909573 -0.334003  C -2.782778 -4.724161 -0.945589  H -2.078710 -5.511596 -1.185410  C -4.132719 -5.018854 -0.867341  H -4.471690 -6.036087 -1.007203  P -0.572411 -3.115517 -1.088346  C -1.357797 -0.437016 -0.764652  C -3.630731 0.141584 0.489153  C -0.019680 -4.845364 1.078208  H -1.000020 -5.273819 0.923349  C 0.416214 -3.784597 0.293961  C -0.311364 -1.355789 -0.799789  C -4.457234 -0.275109 1.540114  H -4.547279 -1.332106 1.750952  C 1.692740 -3.214739 0.489950  C -3.486048 1.531995 0.293000  C -4.187300 2.439934 1.076674  H -4.068261 3.503137 0.921643  C -5.139455 0.628947 2.340612  B -2.772228 -0.896080 -0.318001  C 0.782976 -5.331785 2.101402  H 0.425071 -6.141974 2.722060  C 2.024629 -4.763953 2.342383  C 2.466673 -3.721614 1.541318  H 3.427077 -3.271034 1.752040  C -5.010396 1.988132 2.099479  H -5.533585 2.703245 2.719645  C 1.990186 3.996890 1.541246  H 1.119851 4.603425 1.752103  H 5.067734 1.770991 0.922196  H 5.107553 3.439288 2.720898  H -0.593417 7.396313 -0.629061  H 3.119228 4.851270 3.151670  H 6.702227 -3.184159 -0.630474  H 2.642104 -5.126020 3.152289  H -6.108556 -4.212321 -0.629935  H -5.762246 0.275278 3.150163 |
| RMSD=$\sqrt{\sum\left( r_{6-31+G*}-r_{6-311++G**} \right)^{2}}$=**0.023** | |

*RMSD between the 1CO_2_-(B_3_P_3_-NG) structures optimized at the M06-2X/6-31+G* and M06-2X/6-311++G** level of theory*

| **M06-2X/6-31+G*** | **M06-2X/6-311++G**** |
| --- | --- |
| C 0.814047 -5.196960 2.277789  C -0.480485 -3.803698 0.222227  C 0.914511 -3.617828 0.408934  C -1.190417 -4.697009 1.025710  C -0.546520 -5.388204 2.051245  P 3.343177 0.175284 -1.156897  P -1.266826 -2.904641 -1.181508  C 0.925186 -1.259112 -0.820836  C 3.275129 -2.603826 -0.566124  C 1.575847 -0.012447 -0.818894  C -3.836838 -3.832373 -0.943625  H -3.364328 -4.786797 -1.171504  C -3.047420 -2.676814 -0.809307  C -0.497621 -1.289540 -0.858989  C 3.929215 -3.852468 -0.553784  H 3.335110 -4.752393 -0.419819  C -3.655870 -1.420485 -0.611922  C 4.071862 -1.457730 -0.811740  C 5.453151 -1.586684 -1.017795  H 6.047957 -0.710079 -1.267199  C 5.302231 -3.973171 -0.732714  B 1.722020 -2.531637 -0.391017  C -5.219899 -3.773376 -0.823535  H -5.813096 -4.678537 -0.918599  C -5.836915 -2.539509 -0.615022  C -5.058110 -1.391003 -0.523990  H -5.546377 -0.427718 -0.384055  C 6.071533 -2.830252 -0.949446  H 7.144449 -2.910346 -1.099606  B -2.860544 -0.042032 -0.465908  C -1.261392 -0.128392 -0.734827  C 0.720154 6.321154 -0.725980  C -0.701099 3.973660 -0.399906  C 0.714821 3.898381 -0.381009  C 1.396653 5.113156 -0.556135  H 2.483070 5.108016 -0.580664  C -1.391785 5.176643 -0.560100  H -2.478197 5.184587 -0.593663  C -0.672235 6.359064 -0.717169  H -1.198039 7.299057 -0.851802  P -1.619487 2.446072 -0.313843  C 0.852624 1.199562 -0.681842  C 2.929429 2.456803 0.407921  C -2.583587 2.713715 2.354643  H -2.126034 3.697288 2.432564  C -2.493068 1.969762 1.181623  C -0.536048 1.064431 -0.635419  C 3.314593 3.397668 1.381478  H 2.654999 4.233953 1.599094  C -3.058144 0.691349 1.001256  C 3.787980 1.345108 0.191607  C 4.984969 1.230630 0.900326  H 5.636663 0.376357 0.741419  C 4.497684 3.270171 2.102683  B 1.520197 2.539084 -0.264524  C -3.269301 2.152765 3.430706  H -3.358451 2.701092 4.363548  C -3.831201 0.881989 3.303095  C -3.726958 0.162893 2.109100  H -4.175335 -0.825170 2.036265  C 5.333601 2.183259 1.856481  H 6.254467 2.063851 2.420679  C 1.526712 -4.322540 1.461713  H 2.581589 -4.158439 1.665630  H -2.259293 -4.829187 0.884918  H -1.117548 -6.060883 2.685544  H 5.769764 -4.953332 -0.719637  H 1.314898 -5.718441 3.088128  H -6.919047 -2.474031 -0.541837  H -4.358831 0.444908 4.146518  H 1.285146 7.237421 -0.870611  H 4.761879 4.006632 2.855547  C -2.998416 2.195663 -1.597930  O -3.447322 0.988872 -1.511749  O -3.347870 3.098581 -2.315885 | C 0.811024 -5.204121 2.255608  C -0.480851 -3.799286 0.220779  C 0.909446 -3.614913 0.408376  C -1.185987 -4.697772 1.013202  C -0.543521 -5.393732 2.027929  P 3.329360 0.167334 -1.125042  P -1.267606 -2.887847 -1.159422  C 0.921206 -1.255843 -0.800678  C 3.262817 -2.599425 -0.557906  C 1.571499 -0.014202 -0.800884  C -3.825330 -3.816045 -0.927553  H -3.353237 -4.766497 -1.146841  C -3.039774 -2.663882 -0.798809  C -0.497378 -1.284872 -0.844105  C 3.914015 -3.843693 -0.557238  H 3.322235 -4.740086 -0.431771  C -3.647110 -1.411607 -0.610077  C 4.057546 -1.455490 -0.792035  C 5.434142 -1.582753 -0.993440  H 6.026633 -0.707926 -1.230890  C 5.281224 -3.961804 -0.733222  B 1.714687 -2.526794 -0.380844  C -5.202259 -3.757094 -0.810865  H -5.792169 -4.658726 -0.901160  C -5.817831 -2.527505 -0.611569  C -5.044106 -1.382581 -0.525687  H -5.531688 -0.423873 -0.391745  C 6.048526 -2.821506 -0.934647  H 7.117009 -2.900511 -1.080564  B -2.853802 -0.035147 -0.468506  C -1.256346 -0.125400 -0.727168  C 0.711510 6.296825 -0.727941  C -0.700001 3.957498 -0.395638  C 0.711173 3.885787 -0.376771  C 1.388137 5.096185 -0.555623  H 2.469414 5.092967 -0.578573  C -1.389323 5.154312 -0.558936  H -2.470695 5.158601 -0.593961  C -0.674973 6.332207 -0.718960  H -1.199753 7.266804 -0.855256  P -1.611057 2.436899 -0.310573  C 0.851253 1.195397 -0.669265  C 2.921436 2.452902 0.411887  C -2.570277 2.705913 2.346457  H -2.112674 3.683669 2.424671  C -2.482252 1.967739 1.176096  C -0.533165 1.063070 -0.628009  C 3.308518 3.395805 1.374711  H 2.651767 4.228414 1.588879  C -3.048179 0.694584 0.997326  C 3.779057 1.346062 0.197398  C 4.975530 1.239841 0.897503  H 5.626157 0.391425 0.739528  C 4.490186 3.275389 2.087446  B 1.515579 2.531423 -0.256513  C -3.254174 2.147837 3.417379  H -3.341212 2.692356 4.346742  C -3.816096 0.883859 3.289355  C -3.715284 0.168384 2.099654  H -4.164392 -0.813661 2.026256  C 5.324279 2.194235 1.842697  H 6.244305 2.081113 2.399894  C 1.519657 -4.326208 1.450384  H 2.569857 -4.164981 1.652800  H -2.249174 -4.830388 0.871833  H -1.110959 -6.069783 2.653207  H 5.747000 -4.937272 -0.728638  H 1.309506 -5.729325 3.058111  H -6.895080 -2.463491 -0.540536  H -4.341643 0.449485 4.129070  H 1.271655 7.209873 -0.874764  H 4.755027 4.012066 2.832485  C -2.983377 2.195351 -1.591633  O -3.433269 0.993956 -1.507618  O -3.329003 3.092275 -2.305767 |
| RMSD=$\sqrt{\sum\left( r_{6-31+G*}-r_{6-311++G**} \right)^{2}}$=**0.027** | |

**Figure S1 :** Molecular Electrostatic Potential surface of the 1CO_2_-(B3P3-NG) calculated on the 0.001a.u. electron density isosurface. Level of Theory: M06-2X/6-311++G(3df,3pd). Cyan points indicate the minima of the MEP, and the black points the corresponding maxima. The values of the MEP are given in kJ.mol^-1^ , the color range used is [-79.0; +66.0] kj.mol^-1^.


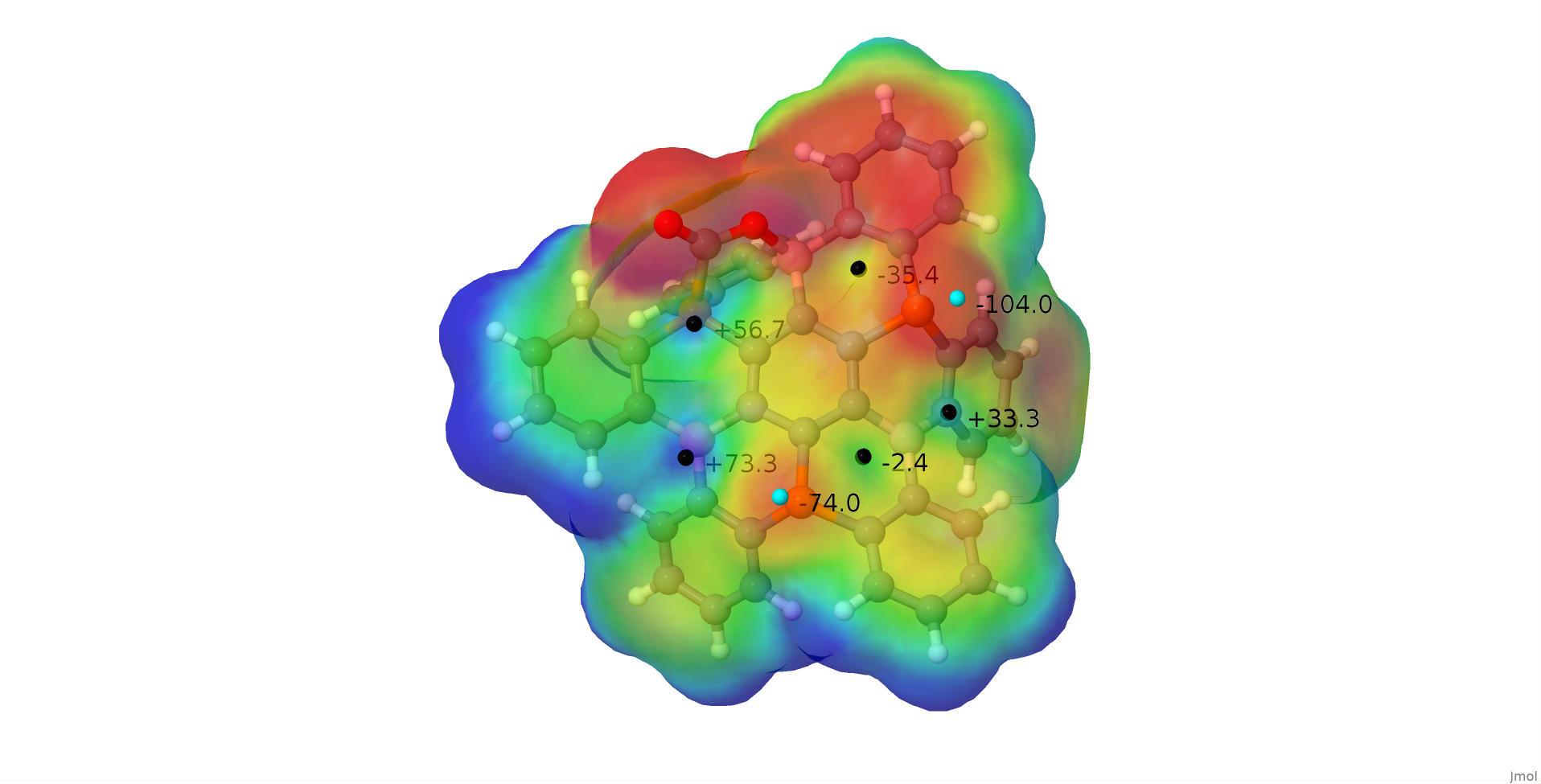


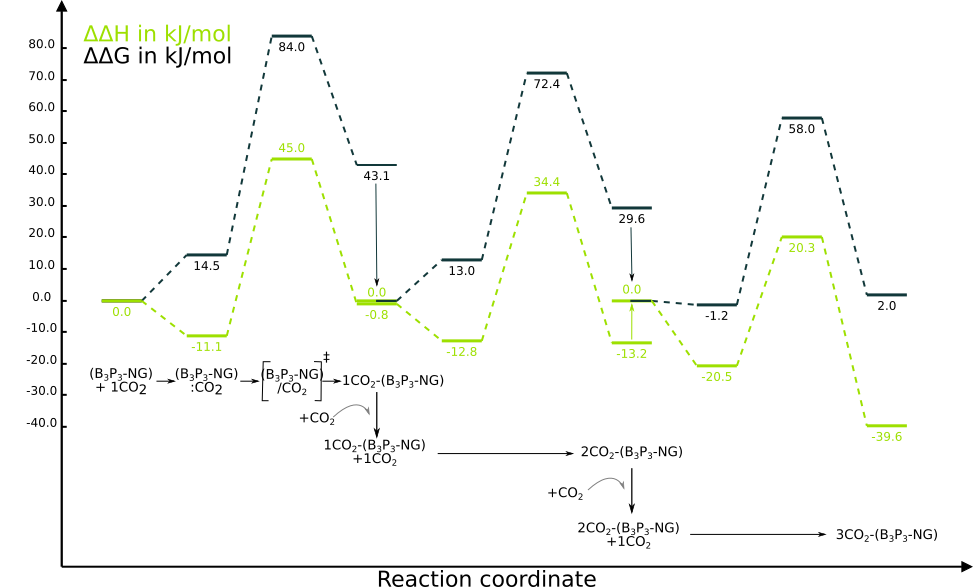


**Figure S2.** Enthalpies (green) and free energies (black) of the successive CO_2_ captures at 298K, calculated using the (M06‐2X/6‐311++G(3df,3dp)//M06‐2X/6‐31+G*) electronic energy and the M06‐2X/6‐31+G* thermal and entropic corrections. In each CO_2_ capture, the enthalpy and free energy of the entrance channel has been defined as 0.0 kJ mol^-1^.
